# Challenges and opportunities for strain verification by whole-genome sequencing

**DOI:** 10.1038/s41598-020-62364-6

**Published:** 2020-04-03

**Authors:** Jenna E. Gallegos, Sergei Hayrynen, Neil R. Adames, Jean Peccoud

**Affiliations:** 1 Colorado State University, Colorado, USA; 2 Genevia Technologies, Helsinki, Finland; 3 GenoFAB, Inc, Fort Collins, USA

**Keywords:** Genome assembly algorithms, Comparative genomics

## Abstract

Laboratory strains, cell lines, and other genetic materials change hands frequently in the life sciences. Despite evidence that such materials are subject to mix-ups, contamination, and accumulation of secondary mutations, verification of strains and samples is not an established part of many experimental workflows. With the plummeting cost of next generation technologies, it is conceivable that whole genome sequencing (WGS) could be applied to routine strain and sample verification in the future. To demonstrate the need for strain validation by WGS, we sequenced haploid yeast segregants derived from a popular commercial mutant collection and identified several unexpected mutations. We determined that available bioinformatics tools may be ill-suited for verification and highlight the importance of finishing reference genomes for commonly used laboratory strains.

## Introduction

The frequent transfer of genetic materials between life science organizations introduces opportunities for quality control issues. Genetic mutations accumulate naturally over time, and human errors in labeling and sample preparation are unavoidable. Anecdotally, it is not uncommon for researchers to complain of samples exhibiting unexpected behaviors, only to later discover that the genetic material they’re working with is not as expected.

Laboratory strains, cell lines, and mutant collections exhibit considerable nucleotide variation and background mutations even among lines thought to be isogenic^1–4^. Despite a growing awareness, cell-line contamination and misidentification are persistent problems, particularly in mammalian cell research^5–9^. Comparably, much less attention has been paid to the potential for similar issues in non-mammalian samples. Yet even commonly used plasmids have been shown to vary dramatically from their published sequence^10^. The problem of plasmid verification has been addressed through the development of a web-based application for assembly of Sanger sequencing reads and alignment of the assembled plasmids with a reference^11^. Strain verification by a similar method will be orders of magnitude more challenging.

The methods currently used to verify samples/strains are biased towards a particular goal. For instance, diagnostic techniques such as PCR, targeted sequencing, or restriction enzyme-based methods are often used to identify whether or not a marker gene or known sequence variant is present, or for analysis of variable repeat regions such as in 16 s rRNA profiling^12,13^. These approaches are limited to particular regions of the genome or are insufficiently sensitive for capturing many types of sequence variations^2,14,15^.

In addition to wasted time and reagents, undetected genetic variation can lead to severe consequences including delays in publishing or patenting and misplaced conclusions that result in product recalls or retractions^16^. Given reports that the life sciences are facing a reproducibility crisis^17^, it is more important than ever for researchers to verify the samples and strains they work with.

As the cost and turnaround time of next generation sequencing continues to decrease, sample and strain verification by whole genome sequencing (WGS) is becoming a more feasible approach^13^. Many tools have been developed for assembling sequenced genomes and detecting variants by aligning sequencing reads to a reference genome^18^, but these tools have been largely developed and validated using human sequencing data. The same tools may not perform well when analyzing microbial sequencing data due to differing ploidy, genome size, and mutation rates^19^. The applicability of assembly and, especially, variant calling tools to microbial sample and strain verification has not been thoroughly explored.

In order to identify the practical obstacles that must be overcome to ultimately implement WGS as a regular part of genetics workflows, we used haploid yeast strains with an unexpected phenotype derived from a mutant collection as a test case.

## Results

### Yeast strain sequencing

As part of a series of yeast cell cycle experiments, we crossed two mutant lines from a knockout collection^20^ to produce *cln3∆ mbp1∆* double mutants in *S. cerevisiae*. *mbp1* and *cln3* knockouts are each individually known to result in an increased critical cell size at the start of S phase^21–23^. When the *cln3∆::kanMX* and *mpb1∆::natMX* mutant lines were crossed, half of the double mutant progeny had a wild-type G1 cell size. When we examined the *cln3* mutant strain, it too had a wild-type-like cell size. Three of the *cln3∆ mbp1∆* double mutant segregants were sequenced using an Illumina MiSeq sequencer: one segregant, 1691, exhibited the unexpected wild-type-like phenotype, the others, 1693 and 1694 (which exhibited the mutant phenotype) were used for comparison.

**Figure 1:**
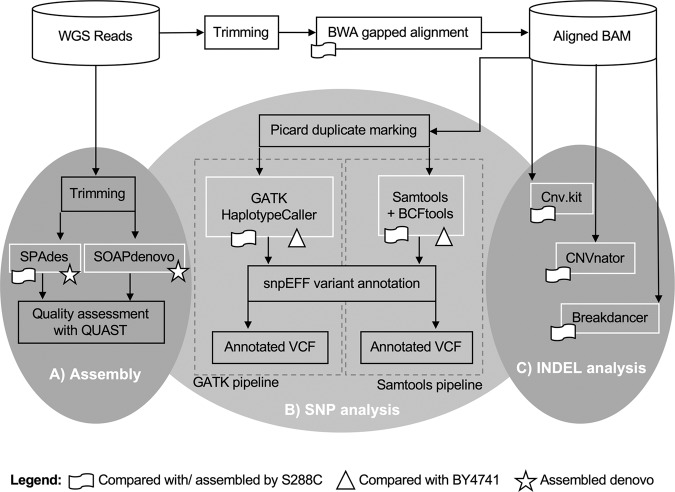
Data analysis pipeline. Software tools compared for each type of analysis are outlined in white. Icons are used to denote whether the analysis was conducted against the S288C reference genome (flag), the BY4741 draft genome (triangle), and or *de novo* (star). (**A)** Reads were assembled using SPAdes (S288C reference-based and *de novo* assembly) and SOAPdenovo (*de novo* only). (**B)** SNPs were analyzed using GATK and Samtools against the S288C reference strain and the BY4741 genetic background. (**C)** INDELs and other structural variants were analyzed against the S288C reference using cnv.kit, CNVnator, and Breakdancer. Software versions and parameters used are detailed in Supplementary Information.

We conducted three different sequence analyses (Fig. 1): (A) Reads were assembled both *de novo* and against the S288C reference genome. (B) Variant finding tools were used to call single nucleotide polymorphisms (SNPs) that varied between strains 1691 and 1693. (C) Copy number variant tools were used to confirm the presence of strain-specific deletions and marker gene insertions (INDELs) and check for additional structural variations.

Because variant calling via genome alignment and variant calling via mapped reads can result in different types of errors^19^, we tested both approaches. For each stage of the analysis, at least two different popular bioinformatic tools were tested. We selected tools partly based on Pabinger, *et al*.’s survey of 205 tools^18^. We tested additional tools to those described herein, but only results from the best performing tools were included in the manuscript. Known and unknown variants were confirmed visually (see Supplementary Figs. 1, 2 for examples) by aligning trimmed reads to the S288C reference genome using Integrated Genomics Viewer (IGV) software (http://www.broadinstitute.org/igv).

### Assembling a genome for verification

An ideal approach to sample verification by WGS would be to sequence the sample, assemble the genome, and then compare the assembled genome to the exact reference genome (the genetic background plus any known variations). Unfortunately, there is not a finished reference genome available for the genetic background used in our analysis (BY4741), despite the fact that it is a commonly used laboratory strain. We thus conducted reference-based assemblies using the closely related S288C genome.

Analyzing a genome assembled *de novo* has a number of additional advantages. For instance, reads that do not align to the reference, such as those for a marker gene insertion or transgene, might be trimmed from the analysis. As such, we repeated the assemblies *de novo*.

The quality of each assembly was compared using the QUAST quality assessment tool for genome assemblies^24^ (Table 1 and Supplementary Table 2). In all metrics, the reference-based assembly exceeded the *de novo* assemblies, and SPAdes^25^ out-performed SOAPdenovo2^26^. Although comparable to the previously published BY4741 draft genome^27^, the quality metrics of all assemblies varied markedly from the S288C reference in number of contigs and N50 values.

**Table 1:** Assembly Quality Metrics.

Method	Strain	# contigs	Total length	N50	# misassembled contigs	Genome fraction (%)	# mismatches per 100 kbp	# indels per 100 kbp
SPAdes (*de novo*)	1691	216	11700934	168266	10	95.994	6.95	1.64
SPAdes (*de novo*)	1693	204	11701059	188980	7	96.026	7.94	1.60
SPAdes (w/ref)	1691	98	11790726	356416	11	96.811	8.57	1.63
SPAdes (w/ref)	1693	101	11774218	332829	7	96.658	10.11	1.73
SOAPdenovo2	1691	283	11660175	156766	8	94.251	3.34	17.67
SOAPdenovo2	1693	284	11642628	179827	10	94.457	3.17	12.25
Reference	S288C	17	12157105	924431				

Comparison of assembly metrics from different approaches.

Cost is a major barrier to using WGS for sample verification and cost directly relates to coverage. In this study, strains were sequenced on a MiSeq for a total of ~2 million, 250 bp paired-end reads, corresponding to a predicted 80x coverage of the 12 Mb yeast genome. Coverage metrics produced with Picard tools show that ~95% of genome was covered with at least 30 reads in all three samples (http://broadinstitute.github.io/picard/).

**Figure 2:**
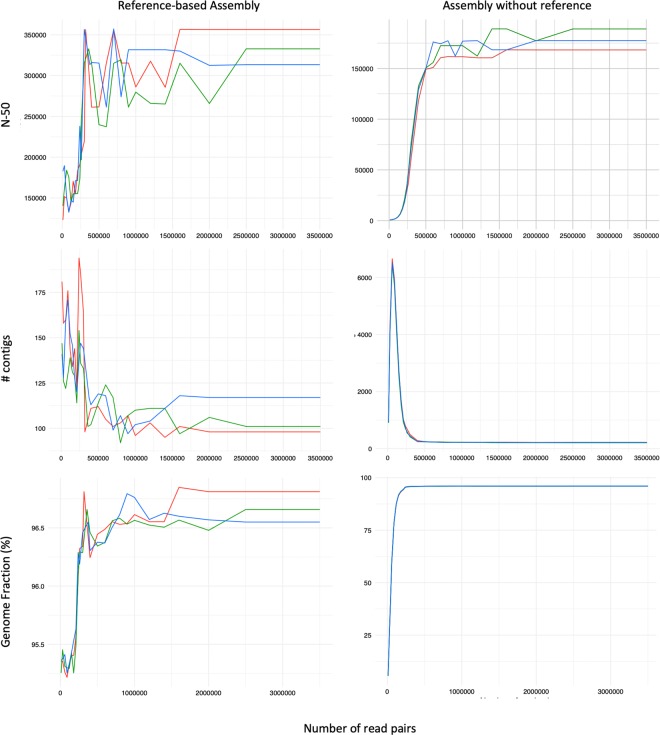
Assembly subsampling analysis. Comparison of the effect of sequencing depth on various metrics (from top to bottom: N50, total number of contigs, and genome fraction) for assemblies against the S288C reference (left) and *de novo* (right). In each case, the X-axis is number of read pairs. The red, green, and blue lines correspond to reads from 1691, 1693, and 1694 respectively.

To determine the minimal cost for which a comparable assembly might have been achieved, we repeated the SPAdes assemblies simulating varying levels of coverage by randomly subsampling the read library. For the majority of the quality assembly metrics compared for both reference-based and *de novo* assembly (Fig. 2 and Supplementary Tables 3 and 4 respectively), the assembly quality began plateauing at around 500,000 read pairs.

Thus, for a haploid yeast genome, a predicted coverage of 10x-30x is sufficient for a draft genome assembly and investing in NGS coverage beyond 10x may not yield notably better assemblies. To generate a more complete genome, it may thus be more prudent to invest in long read sequencing such as PacBio and Oxford Nanopore technologies in order to conduct hybrid assemblies^28–31^, as opposed to increasing short read depth.

A meaningful direct comparison of our assembled genomes with the reference would require a more complete assembly than we were able to accomplish using short reads alone. As such, we conducted the remainder of our analysis by aligning trimmed reads to the reference genome.

### Variant calling from WGS data

The selection of tools for variant calling can drastically influences the results^18,32^. We called variants using^33^ and Samtools^34^ and attempted to confirm each variant visually by inspecting the reads in IGV (see Supplementary Fig. 1 for an example). To simplify the analysis, we focused on only those variants that were discordant between 1691 and 1693.

As has been previously observed^32^, Samtools identified substantially fewer SNPs than GATK (Supplementary Tables 5 vs 6, respectively). This is likely due to the fact that GATK HaplotypeCaller does local reassembly in regions with genomic variation^35^.

Table 2 highlights all of the SNPs that could be confirmed by aligning the reads to the S288C reference genome in IGV. All of these were identified by both GATK and Samtools. The nine variants called by Samtools that were not also called by GATK (unshaded rows in Supplementary Table 5) could be confirmed for both strain 1691 and 1693 by visual inspection of the aligned reads and thus were not truly discordant. The 58 variants identified by GATK but not Samtools (unshaded and lightly shaded rows in Supplementary Table 6) could all either be confirmed for both strains (not discordant) or for neither (possibly not true SNPs). Only two of the variants that were called by both GATK and Samtools could not be confirmed by inspecting the reads in IGV. The combination of GATK and Samtools, as has previously been proposed^32^, was thus a valuable approach for filtering out noise in this case.

**Table 2:** Consensus variants.

Gene	Function/Notes	Variant	differs from ref	Impact
*GDC11* (YER025W)	eIF2 subunit (translation)	G > A	1691	missense
*PPX1* (YHR201C)	hydrolyzes inorganic phosphate	C > T	1693	missense
*TOR2* (YKL203C)	cell cycle	T > G	1693	missense
*DYN1* (YKR054C)	cell cycle	G > T	1691	missense
*SLA2* (YNL243W)	cell cycle	C > T	1691	missense
*CDC1* (YDR182W)	cell cycle	G > T	1693	missense
*SCH9* (YHR205W)	protein kinase	C > T	1693	synonymous
Intergenic	Near Met17 deletion	T > A	1691	intergenic

Eight variants identified by both GATK and Samtools (using reference S288C). These are all the variants called by either tool that could be confirmed visually by aligning the reads to the reference in IGV (see Supplementary Fig. 1 for an example).

Analysis with both tools was repeated using the draft genome for the genetic background strain of the parent, BY4741 (Supplementary Tables 7 and 8). This resulted in a substantially longer list of additional discordant SNPs, none of which could be validated by visual inspection of the reads in IGV.

The analysis using Samtools was also repeated using contigs generated by the SPAdes *de novo* assembly (Supplementary Table 9). This also resulted in a longer list of SNPs, but the quality of the calls could not be assessed, because the tool is designed to be used on dozens of reads, not a single contig. This common variant-finding tool is thus not well suited for use with draft genomes or assembled contigs, both of which could facilitate sample and strain verification.

Of the confirmed SNPs, half occurred in cell cycle related genes (Table 2). None of these are likely to account for the unexpected phenotype observed for 1691. The two variants that were specific to 1691 were located in genes *DYN1* and *SLA2*, both of which are important for cytoskeletal functions^36,37^. *dyn1* or *sla2* loss-of-function slows the cell cycle and would not relieve the cell cycle delay in G1 caused by the *cln3* mutation^36,37^. However, any of these SNPs could potentially impact the interpretation of cell-cycle experiments.

### INDEL analysis

The segregants sequenced were expected to differ from the S288C reference strain at several auxotrophic marker deletions^38^, at their mating type genes (the reference strain is *MAT*α mating type while 1691 and 1693 are *MAT***a**), and by marker gene insertions at *CLN3* and *MBP1* (shaded cells in Table 3). To confirm the presence or absence of these structural changes in 1691 and 1693 we performed copy-number variant analyses using three different tools: Breakdancer^39^, CNVkit^40^, and CNVnator^41^.

**Table 3:** INDEL identification.

TOOL USED:		Breakdancer		CNVkit		CNVnator: 100 bp		CNVnator: 20 bp		Manual	
Gene	Function	1691	1693	1691	1693	1691	1693	1691	1693	1691	1693
*CLN3* (YAL040C)	cell cycle						x	x	x	x	x
*MBP1* (YDL056W)	cell cycle						x	x	x	x	x
*MATALPHA1*/*MATALPHA2* (YCR039C/YCR040W)	mating factors	x	x							x	x
*HIS3* (YOR202W)	auxotrophic marker							x		x	x
*LEU2* (YCL018W)	auxotrophic marker	x	x			x	x	x	x	x	x
*LYS2* (YBR115C)	auxotrophic marker	x	x		x	x	x	x	x	x	x
*URA3* (YEL021W)	auxotrophic marker	x	x				x	x	x	x	x
*MET17* (YLR303W)	auxotrophic marker	x				x		x		x	
*FLO9* (YAL063C)	flocculation	x	x					x	x	x	x
*ENA2*/*ENA5*/*ENA1* (YDR038C/YDR039C/YDR040C)	ATPase pumps				x			x	x	x	x
*ASP3* (YLR157C)	Cell-wall L-asparaginase							x		x	x
***WHI5*** **(YOR083W)**	**cell cycle**							**x**		**x**	
*DDI2* (YFL061W)	DNA damage repair								x	x	x
*SNO3* (YFL060C)	unknown								x	x	x
		Expected INDELS	Unexpected INDELS								

Identifies which of the INDEL analysis tools succeeded in identifying various expected (dark grey shading) and unexpected (light grey shading) INDELS. For CNVnator, the analysis was conducted twice: once with a bin size of 100 bp (recommended for 30x coverage) and once with a bin size of 20 bp. Only those INDELS which could be confirmed manually by aligning the reads to the reference genome (see Supplementary Fig. 2 for an example) were included. INDELS associated with variable repeat regions such as transposons, telomeres, and ribosomal RNA genes were also excluded. The only unexpected INDEL that differed between 1691 and 1693 occurred in the open reading frame of WHI5 (bolded).

Table 3 lists all of the INDELs that could be confirmed visually by aligning the reads to the S288C reference genome in IGV (see Supplementary Fig. 2 for an example, duplications would be difficult to confirm in this manner). CNVkit found only one of the eight known INDELs in the two segregants but was one of the few tools to identify an event in the ATPase pump genes *ENA1*, *ENA2*, and *ENA5*. Without exploring the parameters to identify more known variants, Breakdancer and CNVnator performed comparably. It was only when we reduced the CNVnator search window down to just 20 bp that we were able to identify most of the known INDELs, even though most of the deletions are hundreds of bp in length (Table 3). These parameters also resulted in by far the longest list of variant calls (Supplementary Table 12 versus Supplementary Tables 10, 11, 13), most of which occurred in known repetitive regions (lightly shaded rows in Supplementary Table 12).

**Figure 3:**
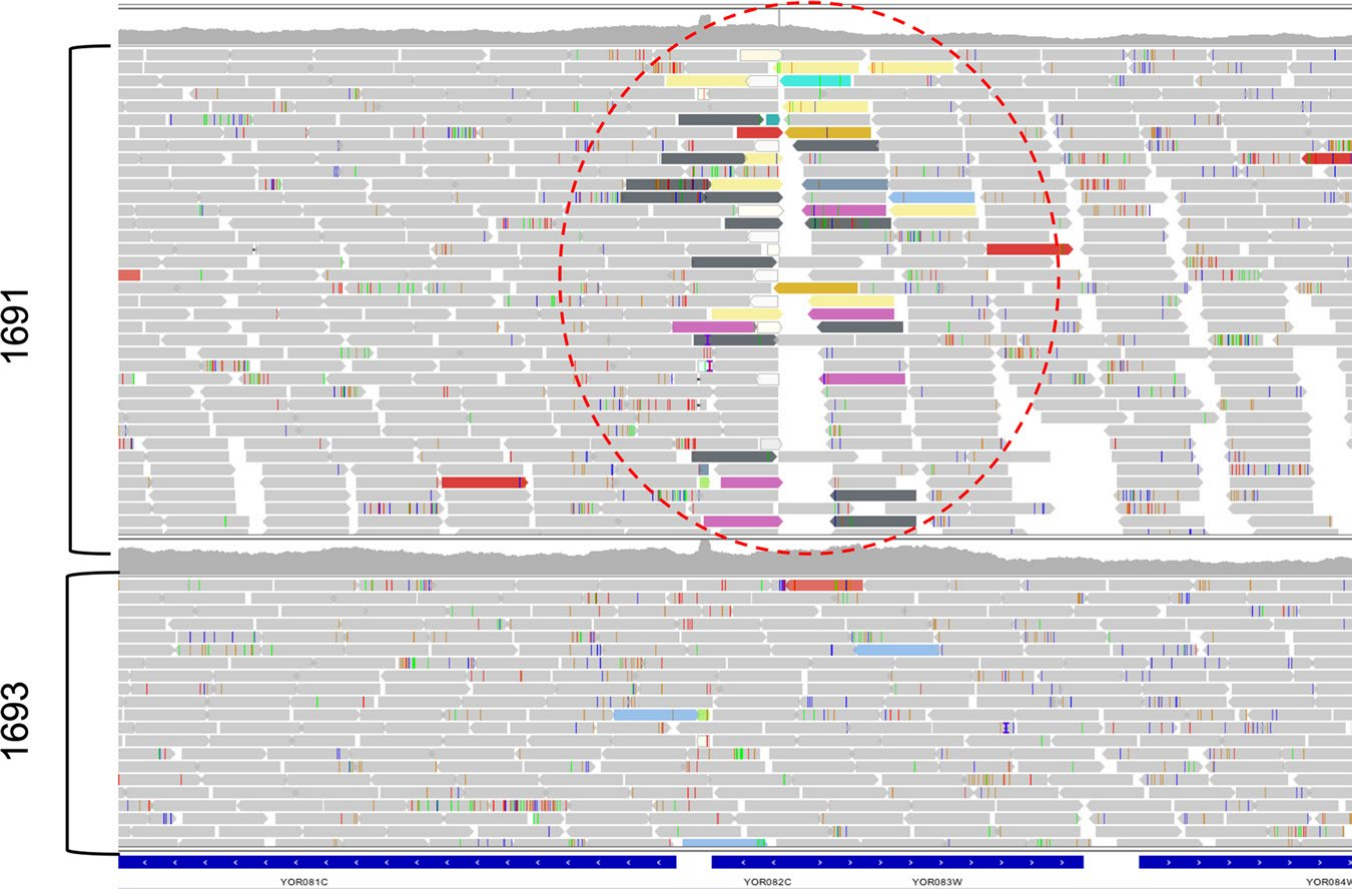
Reads aligned at the WHI5 locus. Each elongated block arrow is a different read. Reads that are colored (not grey) indicate that the mate pair matches a different location in the genome. For each of the colored reads highlighted in the red dashed circle, the mate pair matches a transposon (TY elements).

Only one of the unexpected INDELs, which CNVnator identified as a 1660 bp deletion in *WHI5*, differed between 1691 and 1693. Deletion of *WHI5* has been previously shown to partially suppress the large cell phenotype seen in *cln3* mutants^42–44^. It is, therefore, very likely that the unexpected wild-type-like phenotype observed for 1691 is due to suppression by a mutation in *WHI5*. A visual inspection of the aligned reads at *WHI5* (Fig. 3) suggested that there is a transposon insertion interrupting the *WHI5* reading frame in 1691. The fact that half of the progeny in the cross exhibited the same cell size phenotype as 1691 suggests that the transposon insertion was already present in the *cln3∆::kanMX* mutant parent obtained from the knock-out collection.

## Discussion

Using WGS, we identified eight unexpected SNPs and one unexpected INDEL that differed between segregants derived from a commercial mutant collection. Because this mutant collection is popular in studies of the budding yeast cell cycle, it is pertinent that four of the SNPs identified occurred in cell cycle related genes. This test case demonstrates the value of verifying strains and cell lines from mutant collections by WGS. While this approach was successful in identifying the mutation that was likely the cause for an unexpected phenotype, there may be more changes to the genome that were missed. Clearly, unexpected mutations in common laboratory cell lines cannot be ignored, but the technology needed to get a clear vision of the magnitude of the problem is underdeveloped.

The variant-finding tools used in this analysis were not ideally suited to verification workflows. Most data analysis pipelines, including those described here, rely on ad-hoc or heuristic decision points that require an advanced understanding of the software tools used for analysis^19^. Analyzing the results required manually validating the calls by visualizing the reads, as well as looking up the function of each individual gene – processes that are tedious, time consuming, and potentially error-prone. Additionally, the SNPs/INDELs called differed dramatically depending on the tools and parameters used. None of the tools and parameters tested successfully identified all of the known INDELs (Table 3). It was only when we adjusted the parameters to find the known INDELS, that we identified a large transposon insertion in an important gene. In conclusion, commonly used software tools could not reliably return expected outcomes, were individually too narrow in focus, and collectively too sensitive to parameters to be integrated into a consistent pipeline for verification by WGS.

Before WGS can be used for routine sample and strain validation, genome finishing also needs to be streamlined and made more affordable, such that reference genomes are available for all commonly used laboratory strains. The shortcomings of using short read sequencing in genome assembly have been well reported^45^. In the described use case, the use of short reads significantly hampered our ability to resolve repetitive regions of the genome. This is evident in the fact that most contigs were flanked by transposons. Read alignment across repetitive regions was also ambiguous (for example, Supplementary Fig. 2), complicating the variant analysis; many of variants called were located within transposable elements and telomeres, and near genes encoding tRNAs and ribosomal RNAs (lightly shaded rows in Supplementary Tables 10–13). PacBio sequencing would likely have provided an improved resolution, but it remains prohibitively expensive for verification purposes. And while Oxford Nanopore sequencers are affordable, the reagents and flow cells are costly, and the associated software and algorithms are even less accessible to life science researchers without bioinformatics expertise.

In light of the findings presented in this paper, we would like to suggest a call to action for the development of tools and approaches specifically focused on verification by WGS, in order to ultimately implement WGS as a regular part of genetics workflows, such that all genetic materials are verified by WGS prior to experimentation to improve experimental reproducibility. For instance, it is important that variant finding tools be developed, trained, and validated with microbial sequencing data specifically^19^.

The main shortcoming in the described workflow was its inability to resolve repetitive regions such as transposons. Tools for finding transposons specifically have been developed, but are not routinely employed^46^. Pipelines incorporating tools with different strengths should be used to overcome the false positive and false negatives associated with a particular approach. Variability and repetitive sequences (such as at telomeres, transposons, and ribosomal RNA genes), on the one hand, complicate analysis by WGS, but, on the other hand, emphasize the importance of frequently verifying strains, because the genome is a living dynamic structure, not a rigid set of permanent instructions.

## Methods

### Generating and phenotyping yeast mutants

The mutant collection from which the parental strains used in this study were obtained was generated using background strains BY4741 and BY4742, which differ only in their mating type and the auxotrophic markers *MET15* and *LYS2*^38^. Both were derived from *Saccharomyces cerevisiae* strain FY2 which is a direct descendent of S288C. Both BY4741 and BY4742 are known to differ from S288C by the deletion of four auxotrophic markers. According to a recently prepared draft genome, BY4741 additionally differs from S288C by fewer than 5 SNPs per 100,000 bp^27^.

One of the haploid parents obtained from the collection has the *CLN3* ORF replaced with a marker for G418 resistance (*MAT***a** *cln3∆::kanMX MBP1*). The other has the *MBP1* ORF replaced with a marker for nourseothricin resistance by marker switching of the commercial deletion strain (*MAT*α *CLN3 mbp1∆::natMX*)^47^. These strains were crossed to yield the haploid progeny we analyzed by whole-genome sequencing. The list of strains used in this study is reported in Table 4.

**Table 4:** List of strains used in this study.

Strain ID	Genotype	Source
964 (YSC1021-551214)	*MAT***a** *cln3∆::kanMX his3∆1 leu2∆0 met15∆0 ura3∆0*	^20^
975 (switched YSC1021-550669)	*MAT*α *mbp1∆::natMX his3∆1 leu2∆0 lys2∆0 ura3∆0*	This Study
1691	*MAT***a** *cln3∆::kanMX mbp1∆::natMX his3∆1 leu2∆0 met15∆0 lys2∆0 ura3∆0*	This Study
1693	*MAT***a** *cln3∆::kanMX mbp1∆::natMX his3∆1 leu2∆0 lys2∆0 ura3∆0*	This Study
1694	*MAT***a** *cln3∆::kanMX mbp1∆::natMX his3∆1 leu2∆0 ura3∆0*	This Study

### Sequencing

Each of the strains was sequenced on a Miseq for a total of ~2 million, 250 bp paired-end reads, corresponding to a predicted 80x coverage of the 12 Mb yeast genome. Coverage metrics produced with Picard tools show that ~95% of genome was covered with at least 30 reads in all three samples (http://broadinstitute.github.io/picard/). In addition, the percentage of aligned reads versus total reads was in the range of 97–98% for all three samples.

### Analysis

The following software tools were used in the described analysis: FastQC (https://www.bioinformatics.babraham.ac.uk/projects/fastqc/), Trimgalore! (https://www.bioinformatics.babraham.ac.uk/projects/trim_galore/), cutadapt (https://cutadapt.readthedocs.io/en/stable/), bwa (http://bio-bwa.sourceforge.net), Picard (https://broadinstitute.github.io/picard/), ENSEMBL (http://ensembl.org), CNVnator (https://github.com/abyzovlab/CNVnator), Breakdancer (http://breakdancer.sourceforge.net/), cnv.kit (https://cnvkit.readthedocs.io/en/stable/), Samtools (http://www.htslib.org/doc/samtools.html), BCFtools (https://samtools.github.io/bcftools/bcftools.html), Genome Analysis Toolkit (GATK) (https://software.broadinstitute.org/gatk/), snpEFF (http://snpeff.sourceforge.net/), SOAPdenovo2 (http://soap.genomics.org.cn/soapdenovo.html), SPAdes (http://cab.spbu.ru/software/spades/), and QUAST (http://bioinf.spbau.ru/quast), BLAST (http://doi.org/10.1186/1471-2105-10-421). Default parameters were used unless otherwise noted.

FastQC was used to calculate and visualize sequence quality metrics before and after trimming with Trimgalore!. Samples were aligned to Saccharomyces cerevisiae genome assembly R64-1-1 (Ensembl release 92), corresponding to strain S288C (baker’s yeast), using BWA (v. 0.7.15) with default parameters. Alignment quality of the resulting bam files was assessed using Picard (v.2.9).

CNVnator (v0.3.3) was used for structural variant calling with a bin size of 20 and 100. In addition, copy number variation was assessed with CNVkit (v0.9.3) and Breakdancer (v. 1.3.6).

CNVkit was run with automatic binning and with p-value threshold of 0.000005 for accepting segments and their breakpoints. Copy numbers were called with log2 ratio thresholds of −1.0000000, 0.5849625, 1.3219281, 1.8073549, and 2.1699250 for copy numbers 0 to 4, with thresholds being the upper limits of log2 coverage ratio for each copy number. Thresholds were calculated by adding 0.5 to the integer copy number value for rounding, dividing by ploidy (1), and log2 transforming the result.

Breakdancer was run with default parameters and configuration files generated using script bam2cfg.pl, included in its distribution.

Variant calling was run both with the GATK pipeline and a pipeline consisting of Samtools (v1.8) and Bcftools (v.1.8).

The GATK pipeline was built on GATK version v4.beta.5, except for function CombineGVCFs, which was run with GATK version v3.8, as there was no working version of this function in GATK 4 at the time of setting up the analyses. GATK was run with default parameters and using GATK HaplotypeCaller for calling variants with *—sample_ploidy* set to 1.

Variant calling with Samtools and Bcftools was run with *—ploidy* set to 1 and using multi-allelic calling mode (Bcftools flag *-m*).

Variants called with GATK and Bcftools were annotated using snpEFF (v.4.3 T), a software for variant annotation and predicting effects. Samples aligned to R64-1-1 were annotated using a database for strain S288C provided by snpEFF authors. Samples aligned to BY4741 were annotated using snpEFF database custom built on annotation files downloaded from Saccharomyces Genome Database^48^.

*De novo* assemblies were annotated by BLASTing assembled contigs against the reference genome of strain S288C. In addition, variant calling was performed using our *de novo* assembled genome as a reference. Called variants were annotated by BLASTing their flanking sequences against the reference genome of S288C to find corresponding gene annotations. Function *blastn* of BLAST toolbox (v2.7.1+) was run with following parameters: -*outfmt 6 -max_target_seqs. 1 -max_hsps 1 -num_threads 10 -strand plus*.

*De novo* assemblies were performed with SOAPdenovo2 (v. 2.04) and SPAdes (v. 3.9.0), with SPAdes used for downstream analyses and assembly by subsampling. Reference-based *de novo* assembly, with S288C chromosome sequences used as trusted contigs for i.a. gap closure and repeat resolution, with and without subsampling was also performed with SPAdes (v. 3.9.0).

Qualities of all assemblies were assessed using QUAST, with the reference genome of strain S288C used for benchmarking.

## Supplementary information

Supplementary Information.

## Data Availability

All data needed to repeat the analysis described in this manuscript as well as descriptions of the software tools and parameters used is available in the GitHub repository peccoud/strain-verification.
