# Supplementary material for: Challenges and opportunities for strain verification by whole-genome sequencing
Source: Sci Rep. 2020 Apr 3;10:5873. doi: 10.1038/s41598-020-62364-6 (PMC7125075; doi:10.1038/s41598-020-62364-6)
Supplement: Supplementary file 1 — Supplementary Information. [file 41598_2020_62364_MOESM1_ESM.pdf]

Challenges and opportunities for strain verification by whole-genome sequencing

Supplementary Figures and Data

Jenna E Gallegos[1], Sergei Hayrynen[2], Neil Adames[1], Jean Peccoud[1,3*]

[1.] Colorado State University
[2.] Genevia Technologies
[3.] GenoFAB, Inc.
[*] Corresponding author

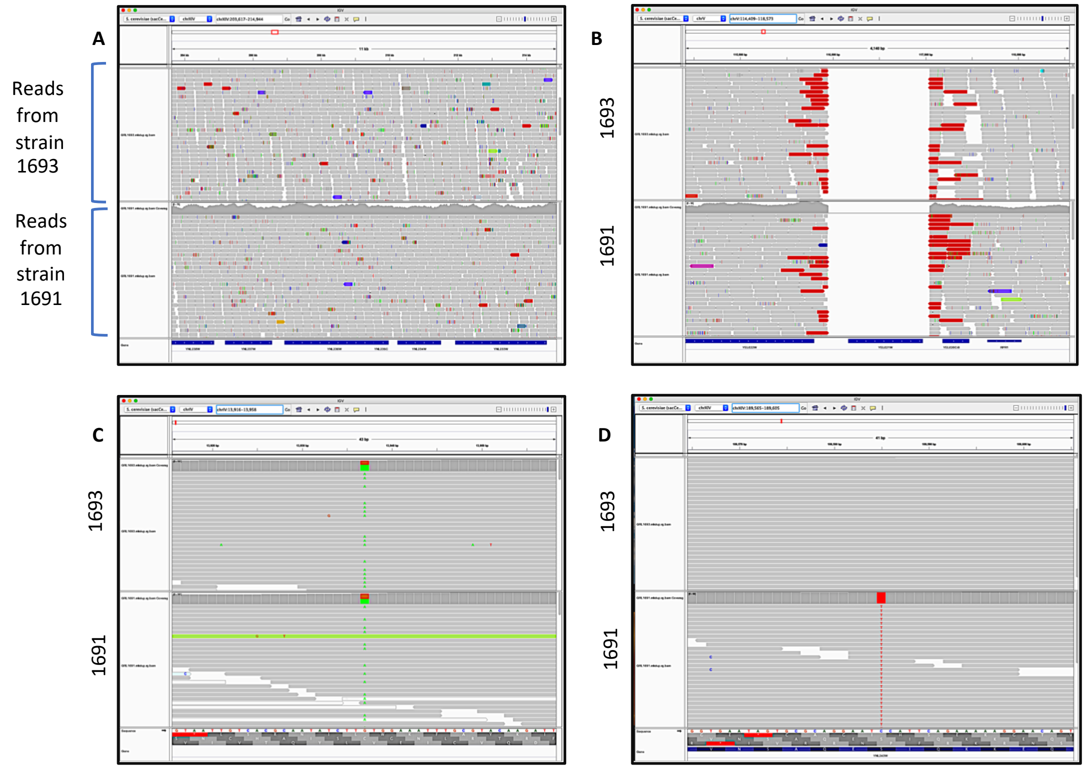

Supplementary Figure 1. Example IGV screenshots. A) Normal alignment where the majority of the reads match the reference genome. B) Deletion C) Non-discordant SNP (present in reads from both 1691 and 1693). D) Discordant SNP (present in 1691 only). Grey bars indicate a matched alignment. Colored bars indicate that the mate pair matches another region of the genome. White bars indicate that the mate pair matches multiple regions of the genome. Colored lines indicate a SNP in the read.

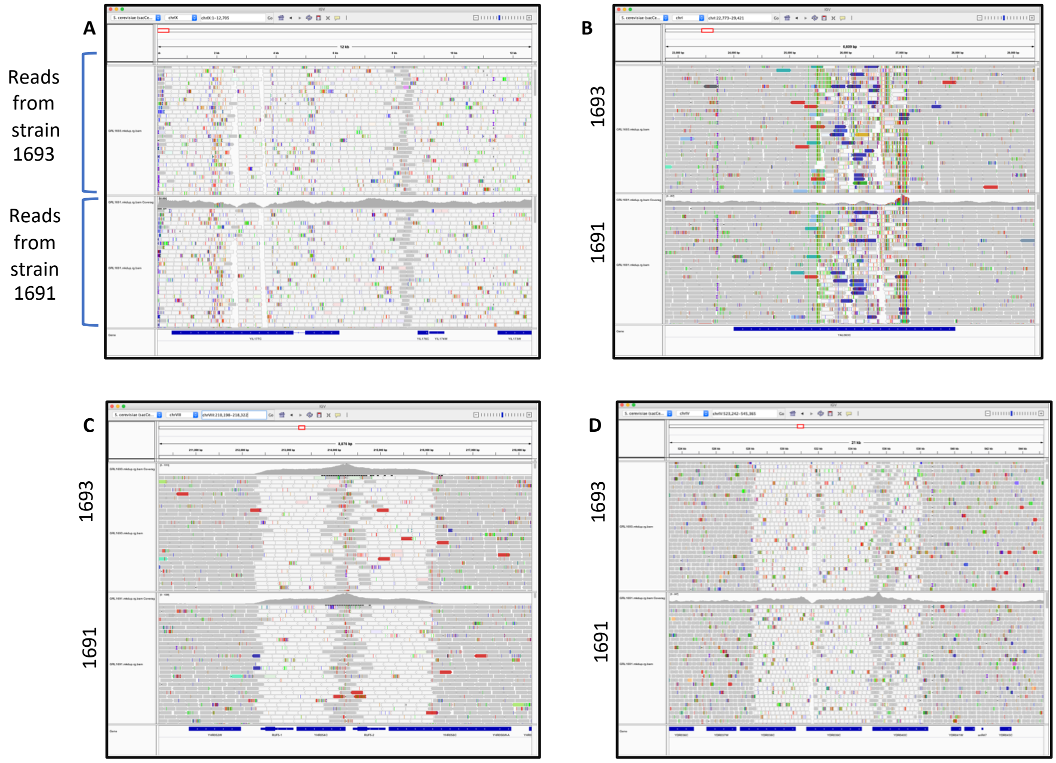

Supplementary Figure 2. Example IGV screenshots at positions for which a structural variant was called by one or more of the CNV-finding tools. A) Telomere, B) Transposon, C) ribosomal RNA genes, D) unexpected structural variant. Grey bars indicate a matched alignment. Colored bars indicate that the mate pair matches another region of the genome. White bars indicate that the mate pair matches multiple regions of the genome. Colored lines indicate a SNP in the read.

Supplementary Table 1: Read Alignment Quality Metrics

Library	1691	1693	1694
CATEGORY	FIRST_OF_PAIR	FIRST_OF_PAIR	FIRST_OF_PAIR
TOTAL_READS	1809588	2105594	2127090
PF_READS	1809588	2105594	2127090
PCT_PF_READS	1	1	1
PF_NOISE_READS	0	0	0
PF_READS_ALIGNED	1789810	2062497	2096632
PCT_PF_READS_ALIGNED	0.98907	0.979532	0.985681
PF_ALIGNED_BASES	440082553	507794906	515044878
PF_HQ_ALIGNED_READS	1613811	1891572	1890738
PF_HQ_ALIGNED_BASES	397272706	466108280	465047908
PF_HQ_ALIGNED_Q20_BASES	382132654	448663996	447045117
PF_HQ_MEDIAN_MISMATCHES	0	0	0
PF_MISMATCH_RATE	0.002786	0.002807	0.003026
PF_HQ_ERROR_RATE	0.00273	0.002754	0.002965
PF_INDEL_RATE	0.000066	0.000064	0.000066
MEAN_READ_LENGTH	247.193158	247.410225	246.869764
READS_ALIGNED_IN_PAIRS	1769206	2037963	2065868
PCT_READS_ALIGNED_IN_PAIRS	0.988488	0.985327	0.985327
BAD_CYCLES	0	0	0
STRAND_BALANCE	0.500084	0.501316	0.500933
PCT_CHIMERAS	0.012503	0.008604	0.013095
PCT_ADAPTER	0.00007	0.000059	0.000051

Supplementary Table 2: Assembly metrics generated by QUAST.

	SPAdes						SOAPdenovo2		
Method	w/out ref			w/ ref			SOAPdenovo2		
Sample	1691	1693	1694	1691	1693	1694	1691	1693	1694
# contigs (>= 0 bp)	609	573	652	395	369	478	1714	1720	1853
# contigs (>= 1000 bp)	162	158	157	81	82	88	210	197	194
# contigs (>= 5000 bp)	109	101	101	65	68	73	114	109	111
# contigs (>= 10000 bp)	100	93	94	63	67	69	103	96	96
# contigs (>= 25000 bp)	86	77	80	58	61	60	87	81	79
# contigs (>= 50000 bp)	67	62	66	49	51	53	69	65	62
Total length (>= 0 bp)	11786208	11782596	11812579	11854059	11835283	11851133	11875194	11864959	11894585
Total length (>= 1000 bp)	11662495	11668214	11669841	11780107	11762442	11748430	11610547	11583184	11595004
Total length (>= 5000 bp)	11556713	11555484	11552077	11748249	11731823	11711253	11422187	11413516	11431730
Total length (>= 10000 bp)	11493176	11501635	11503714	11735246	11726310	11684834	11352487	11328934	11335570
Total length (>= 25000 bp)	11233635	11237257	11249578	11643857	11636863	11525169	11077229	11063858	11059529
Total length (>= 50000 bp)	10534688	10681523	10719345	11346208	11273804	11277876	10417960	10462340	10431188
# contigs	216	204	217	98	101	117	283	284	286
Largest contig	515082	552891	552579	707891	666561	666918	555842	555360	557703
Total length	11700934	11701059	11712421	11790726	11774218	11767469	11660175	11642628	11658908
Reference length	12157105	12157105	12157105	12157105	12157105	12157105	12157105	12157105	12157105
GC (%)	38.09	38.09	38.10	38.10	38.10	38.10	38.07	38.10	38.09
Reference GC (%)	38.15	38.15	38.15	38.15	38.15	38.15	38.15	38.15	38.15
N50	168266	188980	177479	356416	332829	313412	156766	179827	182909
NG50	159840	181742	176940	356416	279390	261450	151193	176254	177551
N75	95888	103340	102845	159749	155699	151954	95467	98165	103264
NG75	88934	97820	95604	152072	147383	142745	89027	91701	95470
L50	21	18	20	12	13	14	23	21	19
LG50	22	19	21	12	14	15	25	22	21
L75	44	39	42	24	26	28	47	43	41
LG75	48	43	45	26	28	30	51	47	45
# misassemblies	10	7	8	11	7	6	10	12	11
# misassembled contigs	10	7	8	11	7	6	8	10	7
Misassembled contigs length	1326701	1098654	1346051	1523523	1593156	1422580	1518239	1250942	1173710
# local misassemblies	15	15	15	12	9	10	139	154	179
# unaligned mis. contigs	0	0	0	0	0	0	11	9	15
# unaligned contigs	7 + 0 part	6 + 0 part	15 + 1 part	7 + 0 part	6 + 0 part	15 + 1 part	7 + 30 part	4 + 17 part	5 + 23 part
Unaligned length	15089	14240	23033	15089	14240	23033	85376	49219	69053
Genome fraction (%)	95.994	96.026	96.014	96.811	96.658	96.55	94.251	94.457	94.405
Duplication ratio	1.001	1.001	1.001	1.001	1.001	1.001	1.01	1.01	1.01
# N's per 100 kbp	0.30	1.09	0.47	0.00	0.54	0.00	1477.35	1250.29	1332.77
# mismatches per 100 kbp	6.95	7.94	7.58	8.57	10.11	8.28	3.34	3.17	2.50
# indels per 100 kbp	1.64	1.60	1.80	1.63	1.73	1.62	17.67	12.25	11.53
Largest alignment	515082	552760	552579	707891	666459	666819	554237	553332	553953
Total aligned length	11680223	11682390	11683547	11772862	11754537	11740664	11519639	11525166	11519379
NA50	155132	181111	177479	326499	279390	313412	151105	171897	177215
NGA50	151977	177322	174318	279454	265699	261450	147050	169460	175719
NA75	93593	102845	95727	152072	150952	147386	92273	93825	102686
NGA75	88598	96035	93593	134042	122851	125395	87977	91150	92313
LA50	22	19	20	12	14	14	25	22	21
LGA50	23	20	22	13	15	15	26	23	22
LA75	46	41	44	25	27	29	50	46	43
LGA75	50	44	47	27	30	31	54	50	47

Supplementary Table 3. Quality metrics for subassemblies by reference. Assemblies were repeated using random susamplings of reads to artificially simulate a decrease in sequencing depth. Assembly was performed using SPAdes against the S288C reference and metrics were calculated by Quast.

sample	reads	# N's per 100 kbp	# contigs	# contigs (>=1000 bp)	# contigs (>=5000 bp)	# contigs (>=25000 bp)	# contigs (>=50000 bp)	Largest contig	# indels per 100 kbp	# misassembled contigs	# misassemblies	# mismatches per 100 kbp	# unaligned contigs	Genome fraction (%)	L50	LA50	LG50	LGA50	N50	NA50	NG50	NGA50	Total length	Total length (>=1000 bp)	Total length (>=10000 bp)	Total length (>=50000 bp)
1691	10000	0	181	172	143	109	77	414717	2.46	14	18	34.76	5 + 1 part	95.355	29	33	31	35	123010	114237	118247	112469	11610511	11604282	11441842	9944968
	30000	0	158	153	128	91	71	513280	3.42	16	18	71.42	5 + 2 part	95.355	24	27	26	29	151569	136305	144428	133732	11613846	11610603	11437229	10367513
	60000	0	160	154	135	103	77	414762	4.4	18	23	82.96	6 + 1 part	95.26	26	30	28	32	150252	118242	126842	115975	11614519	11610400	11480639	10167403
	90000	0	176	171	144	109	78	437853	3.5	19	22	68.41	2 + 1 part	95.216	29	32	32	35	132662	115974	118247	110122	11599133	11595757	11446780	9911236
	120000	0	143	140	121	98	73	451555	4.31	21	27	58.2	3 + 2 part	95.332	24	29	26	32	144047	123673	140081	117671	11616977	11615140	11497374	10300818
	150000	0	134	133	115	85	66	583019	3.84	23	29	41.65	3 + 3 part	95.401	21	26	23	28	170535	144844	162924	135058	11626588	11625929	11515723	10449723
	180000	0	144	142	127	92	67	607528	3.18	18	21	30.91	3 + 2 part	95.406	22	26	24	28	155130	127199	148906	124488	11626664	11625421	11494342	10207655
	210000	0	122	121	105	84	65	607530	3.62	20	25	30.83	3 + 1 part	95.564	20	25	21	27	183986	143991	176753	134377	11637689	11636993	11540193	10640391
	240000	0.74	194	150	110	81	63	666956	2.11	8	9	14.53	3 + 1 part	96.08	19	20	20	22	191172	167037	190158	157711	11724151	11693532	11533040	10592716
	260000	0	186	131	107	83	62	666957	2.38	5	5	14.72	3 + 0 part	96.322	17	18	18	19	203669	192335	193660	189658	11749437	11715417	11604558	10567983
	300000	0.09	164	109	89	69	57	743400	2.09	5	5	11.89	3 + 1 part	96.341	14	14	15	15	219212	219028	219028	204362	11750245	11716658	11619700	10960019
	318795	0	98	81	65	58	49	707891	1.63	11	11	8.57	7 + 0 part	96.811	12	12	12	13	356416	326499	356416	279454	11790726	11780107	11735246	11346208
	400000	2.02	111	93	79	62	53	666974	1.9	7	7	8.7	3 + 2 part	96.245	14	14	14	15	261295	261295	261295	237090	11734126	11721326	11677255	11083735
	500000	2.02	112	95	79	62	53	666973	1.77	6	6	9.45	3 + 1 part	96.445	13	14	14	14	261678	260651	260651	260651	11750473	11739395	11660041	11146031
	600000	0	105	86	71	58	50	666905	1.81	8	9	8.35	6 + 1 part	96.488	13	13	13	14	315403	315403	315403	266278	11757279	11744732	11689586	11199370
	700000	0	101	79	68	57	51	666986	1.71	9	9	8.9	6 + 1 part	96.553	12	13	13	14	356231	315416	315416	261294	11761476	11748016	11711402	11340083
	800000	1.89	103	84	70	56	49	666630	1.71	10	11	8.17	6 + 1 part	96.53	13	13	13	14	315416	315416	315416	282532	11758687	11746399	11699357	11236933
	900000	0	107	84	67	57	51	552578	1.76	10	11	10.71	6 + 1 part	96.538	13	13	13	14	315416	315416	315416	312474	11758759	11744404	11696314	11352308
	1000000	1.89	96	80	67	58	52	666631	1.8	7	7	8.64	6 + 1 part	96.614	13	14	14	15	286028	284556	284556	261293	11765863	11755565	11721630	11356816
	1200000	0	103	83	72	62	53	666818	1.59	10	10	8.52	7 + 1 part	96.553	13	13	13	14	317696	317696	317696	315382	11758403	11745691	11707360	11261654
	1400000	0	95	81	68	61	53	666817	1.84	7	7	8.47	7 + 0 part	96.553	13	14	14	14	285688	279454	279454	279454	11759040	11750741	11709072	11352276
	1600000	0	101	82	65	58	50	707881	1.58	11	11	7.49	7 + 0 part	96.848	12	12	13	13	356620	326499	312595	312595	11794980	11782838	11738856	11374380
	2000000	0	98	81	65	58	49	707891	1.63	11	11	8.57	7 + 0 part	96.811	12	12	12	13	356416	326499	356416	279454	11790726	11780107	11735246	11346208
	2500000	0	98	81	65	58	49	707891	1.63	11	11	8.57	7 + 0 part	96.811	12	12	12	13	356416	326499	356416	279454	11790726	11780107	11735246	11346208
	3000000	0	98	81	65	58	49	707891	1.63	11	11	8.57	7 + 0 part	96.811	12	12	12	13	356416	326499	356416	279454	11790726	11780107	11735246	11346208
	3500000	0	98	81	65	58	49	707891	1.63	11	11	8.57	7 + 0 part	96.811	12	12	12	13	356416	326499	356416	279454	11790726	11780107	11735246	11346208
1693	10000	0	147	138	122	93	71	512281	3.74	20	24	49.15	9 + 2 part	95.254	23	26	25	29	140239	125508	132940	117620	11598257	11592376	11487232	10335469
	30000	0	126	122	109	84	69	505503	4.57	19	25	83.5	5 + 2 part	95.453	21	24	22	26	161657	144664	160396	134009	11623751	11621610	11544303	10677820
	60000	0	122	121	104	82	63	580181	4.44	19	24	91.23	3 + 1 part	95.31	19	21	20	23	184052	155037	182607	150252	11614836	11614331	11510722	10610327
	90000	0	130	129	114	85	68	495837	4.27	19	25	75.67	2 + 1 part	95.298	20	24	21	26	175543	144325	174855	135102	11606934	11606429	11494918	10625229
	120000	0	139	136	121	99	72	451542	3.9	27	33	61.53	4 + 2 part	95.293	24	30	26	32	144845	118339	137144	117865	11621776	11619826	11516043	10365974
	150000	0	131	125	112	92	70	755428	3.07	23	33	44.67	3 + 1 part	95.428	21	26	22	28	155133	140238	153289	125393	11630351	11626645	11532748	10599013
	180000	0	129	126	110	90	71	497518	3.2	22	30	32.83	5 + 1 part	95.254	22	28	24	30	155326	134717	151482	125667	11601431	11599491	11489301	10677126
	210000	0	114	111	98	84	70	1027956	3.18	22	33	26.78	4 + 1 part	95.485	19	26	20	28	155391	141102	153293	134183	11625640	11623492	11572256	10915605
	240000	0	154	129	106	86	68	557523	2.05	11	11	17.83	4 + 1 part	96.111	19	21	21	22	173590	155677	162143	155132	11711496	11694772	11573510	10776603
	260000	0	136	99	80	70	60	666692	2.17	7	7	14.16	6 + 2 part	96.293	15	15	15	16	244870	244870	244870	239164	11739553	11714982	11644747	11157434
	300000	0	133	99	81	63	53	666634	2.07	10	12	12.04	5 + 2 part	96.287	13	14	14	14	315415	263156	263156	263156	11736384	11713760	11612564	11094187
	360019	0.54	101	82	68	61	51	666561	1.73	7	7	10.11	6 + 0 part	96.658	13	14	14	15	332829	279390	279390	265699	11774218	11762442	11726310	11273804
	400000	0	102	81	68	56	50	666284	1.91	7	7	11.18	6 + 1 part	96.46	14	14	14	15	314974	314974	314974	260326	11748060	11734262	11674075	11324814
	500000	0	114	92	77	67	58	666475	1.68	5	5	7.26	6 + 2 part	96.344	15	15	16	16	239814	239814	220452	219562	11734659	11719702	11657184	11231156
	600000	0	124	100	83	70	57	666706	1.55	5	5	7.23	6 + 1 part	96.375	15	16	16	17	237320	212429	224082	204132	11740465	11723923	11668479	11007490
	700000	0	117	91	74	66	53	666863	1.78	8	9	7.33	6 + 1 part	96.561	13	14	14	15	315224	261352	279621	259773	11763183	11745304	11693850	11097745
	800000	0	92	73	65	57	49	666893	1.68	6	6	7.91	5 + 1 part	96.583	13	13	14	14	319353	319029	315224	315224	11762970	11750830	11725852	11306259
	900000	0	107	89	75	69	54	666872	1.76	8	8	7.87	6 + 0 part	96.532	14	14	15	15	261400	261400	261106	261106	11758542	11746532	11711515	11086209
	1000000	0	110	88	72	65	51	561640	1.65	8	8	8.32	5 + 2 part	96.565	14	14	14	15	279792	265699	279792	261289	11762018	11747897	11711014	11084325
	1200000	0	111	92	76	66	51	666869	1.6	8	8	7.87	6 + 0 part	96.524	14	14	15	15	265973	261289	261289	260323	11757371	11745242	11704860	10993491
	1400000	0	111	90	76	65	53	666878	1.69	4	4	8.95	7 + 0 part	96.506	14	14	15	15	265170	265170	261291	261291	11755960	11742744	11707469	11071418
	1600000	0	97	78	67	60	51	666560	1.68	6	6	9.31	5 + 1 part	96.567	13	13	14	14	315226	315226	279859	279859	11760723	11748592	11719603	11289421
	2000000	0.54	106	85	72	64	53	666103	1.65	6	6	9.43	6 + 0 part	96.479	14	14	15	15	265853	265699	261294	261294	11751313	11738299	11703195	11185031
	2500000	0.54	101	82	68	61	51	666561	1.73	7	7	10.11	6 + 0 part	96.658	13	14	14	15	332829	279390	279390	265699	11774218	11762442	11726310	11273804
	3000000	0.54	101	82	68	61	51	666561	1.73	7	7	10.11	6 + 0 part	96.658	13	14	14	15	332829	279390	279390	265699	11774218	11762442	11726310	11273804
	3500000	0.54	101	82	68	61	51	666561	1.73	7	7	10.11	6 + 0 part	96.658	13	14	14	15	332829	279390	279390	265699	11774218	11762442	11726310	11273804
1694	10000	0	141	137	115	84	62	513394	3.13	15	18	35.69	6 + 1 part	95.363	19	21	21	23	182768	165207	175695	153244	11608068	11605194	11485217	10314614
	30000	0	128	125	108	78	63	546691	4.16	20	28	64.27	4 + 2 part	95.373	18	25	19	26	189613	144830	179224	143853	11619943	11618328	11488610	10687582
	60000	0	159	150	131	99	72	539100	3.69	21	29	74.21	6 + 0 part	95.412	23	29	24	31	150251	118348	143513	112962	11621495	11616252	11477314	10159584
	90000	0	171	163	137	104	72	426269	3.51	19	24	62.85	6 + 2 part	95.254	26	31	28	33	132750	122842	131191	118249	11604752	11599979	11444534	9951664
	120000	0	152	147	130	105	73	420201	4.28	23	28	52.95	4 + 3 part	95.332	28	32	29	34	147571	122953	143989	115971	11614741	11611682	11526847	10034493
	150000	0	146	139	121	94	68	821628	3.76	21	30	39.81	3 + 4 part	95.428	24	30	25	32	144845	132660	143990	125514	11631944	11627301	11501839	10285061
	180000	0	128	126	109	85	68	611363	3.38	26	36	32.98	3 + 2 part	95.532	20	28	22	30	171310	126322	162250	124574	11637162	11635928	11538120	10676180
	210000	0	120	117	104	83	65	700271	3.55	23	34	31.64	4 + 2 part	95.631	17	25	19	27	171416	133032	162916	126471	11653996	11652042	11572790	10678069
	240000	0	137	110	92	70	58	666584	1.95	12	14	14.42	4 + 1 part	96.289	13	16	14	17	237981	187151	223877	181745	11736636	11719086	11636059	10926445
	260000	0.23	147	122	93	75	60	666623	1.83	9	9	12.49	5 + 1 part	96.188	16	17	17	19	196695	185917	189660	176273	11722272	11706776	11604474	10805077
	300000	0	144	93	79	65	51	707289	1.89	8	9	11.12	5 + 2 part	96.46	12	13	13	13	356506	261924	262045	261924	11758916	11726497	11662143	11022998
	375385	0	117	88	73	60	53	666918	1.62	6	6	8.28	15 + 1 part	96.55	14	14	15	15	313412	313412	261450	261450	11767469	11748430	11684834	11277876
	400000	0	113	89	73	62	52	783617	2.01	7	7	9.31	4 + 1 part	96.305	12	14	13	15	316097	260320	260320	257070	11731285	11715507	11653698	11135012
	500000	0	119	91	77	62	55	547905	1.67	3	4	8.76	6 + 1 part	96.377	13	14	14	14	315370	285793	286602	285793	11746208	11727129	11668394	11244418
	600000	0.23	118	91	77	62	55	743001	1.61	7	7	9.22	8 + 1 part	96.37	13	14	14	15	261409	260325	260325	239785	11741087	11723196	11655502	11242261
	700000	0.74	99	81	69	59	54	546109	1.59	11	11	9.32	8 + 1 part	96.506	13	14	14	14	357274	285878	286075	285878	11757384	11745221	11708738	11396577
	800000	0	107	89	74	62	55	536085	1.65	11	11	9.48	8 + 1 part	96.614	14	15	15	16	274011	261464	261464	260325	11770449	11759219	11706138	11301418
	900000	0	97	75	65	55	52	666721	1.7	10	10	11.09	7 + 1 part	96.794	13	14	14	14	331660	319115	319439	319115	11791511	11776739	11738918	11487469
	1000000	0	102	77	65	56	51	666704	1.51	12	12	10.54	8 + 2 part	96.762	13	14	14	14	331660	319127	319936	319127	11791625	11775141	11722438	11448526
	1200000	0.88	104	78	63	56	52	743057	1.78	8	8	9.8	11 + 2 part	96.572	12	13	13	14	331660	279248	279387	261852	11767745	11750839	11703058	11482563
	1400000	0	111	78	66	56	51	666703	1.69	11	11	9.6	10 + 2 part	96.626	13	14	14	14	331660	312482	312621	312482	11774099	11752425	11712872	11404187
	1600000	0	118	87	71	57	52	666726	1.67	8	9	8.54	12 + 1 part	96.6	13	14	14	14	330074	312505	312644	312505	11772736	11752460	11690269	11341651
	2000000	0	117	89	73	59	52	666888	1.52	6	6	8.39	14 + 1 part	96.569	14	14	14	14	312535	312535	312535	312535	11769018	11751079	11690459	11266793
	2500000	0	117	88	73	60	53	666918	1.62	6	6	8.28	15 + 1 part	96.55	14	14	15	15	313412	313412	261450	261450	11767469	11748430	11684834	11277876
	3000000	0	117	88	73	60	53	666918	1.62	6	6	8.28	15 + 1 part	96.55	14	14	15	15	313412	313412	261450	261450	11767469	11748430	11684834	11277876
	3500000	0	117	88	73	60	53	666918	1.62	6	6	8.28	15 + 1 part	96.55	14	14	15	15	313412	313412	261450	261450	11767469	11748430	11684834	11277876

Supplementary Table 4. Quality metrics for subassemblies without reference. Assemblies were repeated using random susamplings of reads to artificially simulate a decrease in sequencing depth. Assembly was performed *de novo* using SPAdes and metrics were calculated by Quast.

sample	reads	# N's per 100 kbp	# contigs	# contigs (>=1000 bp)	# contigs (>=5000 bp)	# contigs (>=25000 bp)	# contigs (>=50000 bp)	Largest contig	# indels per 100 kbp	# misassembled contigs	# misassemblies	# mismatches per 100 kbp	# unaligned contigs	Genome fraction (%)	L50	LA50	LG50	LGA50	N50	NA50	NG50	NGA50	Total length	Total length (>=1000 bp)	Total length (>=10000 bp)	Total length (>=50000 bp)
	10000	0	918	78	7	0	0	9262	10.08	36	36	223.1	9 + 0 part	5.63	320	341			684	667		-	698996	157430	0	0
	30000	1.41	4320	775	7	1	1	80320	8.06	111	116	212.36	22 + 2 part	28.977	1495	1542			802	787		-	3556960	1138974	92488	80320
	60000	2.69	6661	2827	21	1	1	85902	6.86	122	123	184.72	16 + 8 part	60.645	1935	1987	4441	4546	1220	1194	730	716	7432400	4700722	121863	85902
	90000	4.86	5953	3696	137	2	1	85902	6.9	125	129	146.53	11 + 4 part	78.988	1460	1504	2153	2217	2017	1967	1581	1538	9671067	8025551	227238	85902
	120000	5.79	4275	3245	456	1	1	85903	6.55	99	103	113.65	6 + 4 part	87.597	917	948	1138	1176	3469	3386	3045	2953	10712036	9953916	832326	85903
	150000	5.81	2727	2325	749	11	0	46238	6.71	76	77	89.66	4 + 3 part	91.506	538	558	620	642	6279	6079	5742	5592	11180707	10884360	2972572	0
	180000	4.13	1719	1567	786	44	4	85903	5.82	48	50	67.92	5 + 1 part	93.204	322	335	359	373	10875	10507	10331	9700	11381878	11272509	6218665	287420
	210000	3.31	1034	983	622	114	16	109764	5.48	46	47	51.15	4 + 0 part	94.131	183	191	201	210	18706	18093	17864	17163	11489193	11451132	8895043	1062124
	240000	11.16	782	692	490	162	35	97506	3.14	21	21	30.27	3 + 1 part	95.394	130	132	139	141	29035	28448	28092	27789	11651486	11588842	10280064	2353786
	260000	15.81	695	572	411	168	51	198779	2.96	24	26	24.3	5 + 2 part	95.564	99	103	106	110	36009	35517	35208	34938	11685540	11604039	10641672	3826858
	300000	14.25	496	391	275	156	80	253025	2.96	18	19	17.69	5 + 2 part	95.716	62	63	66	67	61594	60030	60174	57258	11701277	11632360	11127668	6907131
	400000	2.64	280	226	156	111	80	356308	2.33	9	10	12.69	4 + 0 part	95.834	33	35	35	37	118460	112961	112961	109336	11691487	11654203	11428200	9739303
1691	500000	3.36	244	190	129	95	73	445152	2.14	7	8	11.26	4 + 0 part	95.908	25	26	27	28	149182	133862	133862	132459	11696837	11659319	11427219	10303599
	600000	2	231	172	121	89	70	515060	1.97	9	10	8.92	5 + 2 part	95.954	24	25	25	27	151017	145472	150578	132732	11702346	11661444	11454118	10434496
	700000	0.43	233	169	110	84	69	552455	1.86	10	11	7.18	5 + 2 part	95.971	22	23	23	25	160541	149182	155132	145367	11703141	11658429	11450842	10611287
	800000	0.56	223	163	112	84	67	550066	2.28	13	13	8.76	5 + 2 part	95.991	22	24	23	25	161749	149182	160089	148690	11703822	11661597	11458825	10553927
	900000	0.3	221	162	114	85	67	550064	1.97	13	13	8.45	5 + 2 part	95.996	22	24	23	26	161549	150254	160089	133077	11702002	11660437	11466770	10468001
	1000000	0.38	219	164	111	83	66	541247	1.93	11	11	8.72	5 + 2 part	96.031	22	23	23	25	161617	150254	160089	145367	11706041	11667682	11471305	10516627
	1200000	0.47	213	152	104	83	67	541247	1.83	13	13	8.43	6 + 2 part	95.966	21	22	22	24	160486	149184	155242	145033	11697217	11653977	11497448	10631114
	1400000	0.3	217	160	104	83	69	552579	1.68	11	11	7.06	7 + 0 part	95.994	21	21	22	23	160485	155418	155418	149184	11700593	11660658	11486659	10720463
	1600000	0.3	222	164	106	84	67	515081	1.7	13	13	5.95	7 + 0 part	96.01	21	21	22	23	168354	167932	159840	150253	11704019	11663095	11496685	10604280
	2000000	0.3	216	162	107	86	67	515082	1.67	11	11	6.92	7 + 0 part	95.993	21	22	22	23	168266	155132	159840	151977	11700815	11662376	11501971	10543483
	2500000	0.3	216	162	107	86	67	515082	1.67	11	11	6.92	7 + 0 part	95.993	21	22	22	23	168266	155132	159840	151977	11700815	11662376	11501971	10543483
	3000000	0.3	216	162	107	86	67	515082	1.67	11	11	6.92	7 + 0 part	95.993	21	22	22	23	168266	155132	159840	151977	11700815	11662376	11501971	10543483
	3500000	0.3	216	162	107	86	67	515082	1.67	11	11	6.92	7 + 0 part	95.993	21	22	22	23	168266	155132	159840	151977	11700815	11662376	11501971	10543483
	10000	0	901	61	5	0	0	18676	13.67	31	36	233.17	12 + 2 part	5.535	304	327			682	656		-	691306	151242	18676	0
	30000	3.52	4073	782	7	1	1	85569	10.44	89	94	227.94	19 + 7 part	27.643	1397	1447			826	802		-	3405864	1147831	85569	85569
	60000	9.18	6480	2845	26	1	1	85902	8.99	119	124	197.6	17 + 10 part	60.402	1869	1925	4329	4447	1253	1220	748	728	7403942	4789360	129991	85902
	90000	10.08	5716	3651	150	1	1	85902	8.41	112	119	159.7	8 + 7 part	78.623	1383	1428	2064	2131	2121	2072	1632	1583	9620193	8113156	231754	85902
	120000	10.26	4032	3097	508	3	1	85902	7.35	76	78	116.95	11 + 5 part	87.679	863	890	1065	1098	3773	3682	3330	3230	10717043	10031528	987327	85902
	150000	10.16	2544	2195	770	12	1	85902	6.63	61	64	87.97	6 + 1 part	91.741	492	512	565	588	6745	6503	6194	6026	11205553	10951083	3444361	85902
	180000	7.8	1544	1418	752	49	2	85902	5.89	49	51	63.81	8 + 1 part	93.398	282	296	314	330	12158	11611	11148	10706	11405758	11314492	6728208	147278
	210000	5.39	923	870	610	123	14	97037	4.91	41	44	45.95	4 + 2 part	94.216	169	177	185	193	21246	20594	20492	19311	11494736	11456495	9091378	959679
	240000	4.21	687	606	444	150	53	141563	3.78	17	17	32.79	4 + 4 part	95.424	103	106	111	114	33953	33392	31864	30765	11650521	11592198	10446738	3786853
	260000	3.25	603	503	363	156	70	148513	3.66	17	17	25.87	5 + 3 part	95.623	81	82	86	87	46480	45952	44087	43585	11682918	11612324	10714169	5354699
	300000	2.06	410	324	240	141	85	265916	2.71	16	18	16.65	6 + 2 part	95.764	50	54	53	57	77276	70669	71262	68090	11693098	11632240	11195540	8033220
	400000	6.39	249	192	137	101	76	451903	2.29	12	13	10.74	5 + 2 part	95.86	27	29	29	31	132745	126470	132502	124512	11688093	11647780	11428168	10154443
1693	500000	0.56	236	177	122	93	74	451902	2.17	7	8	9.05	5 + 1 part	95.902	26	26	27	28	150487	148689	148689	132857	11692739	11650663	11464975	10459646
	600000	0.38	228	171	122	90	68	515066	1.8	7	8	9.31	5 + 2 part	95.916	23	24	25	26	155951	150488	151168	133075	11692707	11653697	11472014	10281363
	700000	0.73	228	170	113	87	63	552781	1.78	6	6	7.08	6 + 1 part	95.911	20	21	22	22	172450	162108	155950	155880	11691500	11651075	11464927	10284973
	800000	0.48	227	169	111	85	65	552781	1.94	8	8	7.93	6 + 1 part	95.995	20	21	22	23	172450	162108	156743	155880	11701019	11660955	11486274	10422348
	900000	0.9	224	170	110	84	67	552777	2.02	7	7	8.33	6 + 0 part	96.037	21	21	22	22	172449	168866	162236	162109	11706223	11668151	11479182	10566636
	1000000	0.38	223	168	109	84	66	552888	1.94	9	9	8.72	5 + 2 part	96.009	22	22	23	24	172450	162109	162236	150577	11703605	11665150	11473408	10530679
	1200000	0.47	218	167	108	84	66	515675	1.76	6	6	8.32	6 + 0 part	95.994	21	22	22	23	162235	159993	160094	156557	11699859	11663755	11488964	10526687
	1400000	0.56	208	159	104	78	63	552963	1.86	7	7	8.35	7 + 0 part	96.005	18	19	20	20	189039	181192	177322	177322	11700455	11665894	11498935	10613309
	1600000	0.56	206	155	102	78	63	552961	1.88	9	9	8.3	7 + 0 part	95.977	18	19	20	20	189041	181192	177322	177322	11696462	11660207	11504689	10657802
	2000000	1.01	207	161	103	79	63	527849	1.53	8	8	7.69	6 + 0 part	96.008	19	19	20	21	177322	177322	172450	162200	11699350	11666871	11486052	10646223
	2500000	1.09	204	158	101	77	62	552891	1.6	7	7	7.94	6 + 0 part	96.026	18	19	19	20	188980	181111	181742	177322	11701059	11668214	11501635	10681523
	3000000	1.09	204	158	101	77	62	552891	1.6	7	7	7.94	6 + 0 part	96.026	18	19	19	20	188980	181111	181742	177322	11701059	11668214	11501635	10681523
	3500000	1.09	204	158	101	77	62	552891	1.6	7	7	7.94	6 + 0 part	96.026	18	19	19	20	188980	181111	181742	177322	11701059	11668214	11501635	10681523
	10000	2.77	938	70	8	0	0	10219	14.79	34	38	238.96	12 + 2 part	5.783	322	348			688	669		-	722653	159968	10219	0
	30000	3.2	4149	740	10	2	0	36411	8.58	96	98	198.25	22 + 3 part	27.99	1426	1469			812	799		-	3441137	1106328	86590	0
	60000	6.11	6518	2820	26	2	1	60084	8.71	151	157	185.5	19 + 11 part	60.029	1888	1950	4414	4546	1236	1210	730	711	7360398	4715571	120352	60084
	90000	8.41	5876	3653	129	2	1	85902	8.5	147	153	147.82	17 + 11 part	78.661	1421	1483	2124	2209	2041	1966	1599	1546	9631930	8006988	232359	85902
	120000	9.62	4278	3243	458	3	1	81458	7.72	86	91	108.95	12 + 5 part	87.559	915	949	1136	1176	3508	3399	3068	2985	10709040	9952226	813377	81458
	150000	9.01	2730	2329	742	8	1	85902	6.28	64	66	75.15	5 + 1 part	91.779	538	558	618	642	6185	5997	5601	5451	11208576	10915711	3016675	85902
	180000	5.43	1675	1514	801	37	2	85902	5.36	55	59	56.53	4 + 1 part	93.454	319	333	354	369	11212	10814	10600	10087	11413062	11297204	6377493	139373
	210000	4.86	1011	951	654	106	14	119505	4.58	40	43	41.11	6 + 0 part	94.353	193	202	211	220	18068	17873	17418	17137	11516431	11472640	9008380	915284
	240000	3.6	728	641	468	156	51	152556	3.7	26	26	27.94	5 + 1 part	95.558	109	115	117	124	30670	29877	29732	28678	11674357	11614189	10268126	3601895
	260000	1.88	549	478	366	163	71	227697	2.88	23	23	21.95	6 + 2 part	95.71	78	82	83	87	45010	41957	42138	40704	11681288	11630940	10898745	5543360
	300000	1.71	450	368	254	149	89	230685	2.94	13	16	16.1	5 + 3 part	95.849	55	55	58	59	71575	70010	69401	66080	11705264	11648286	11170828	7951294
	400000	8.01	244	188	134	99	74	539859	2.56	11	12	9.86	3 + 4 part	95.864	26	27	28	29	129232	124075	124216	122331	11692019	11651986	11455092	10146619
1694	500000	0.73	245	179	126	96	70	540161	1.95	10	11	8.41	5 + 2 part	95.955	24	26	25	27	150160	126783	133313	124577	11710045	11664802	11475888	10206556
	600000	0.66	232	170	117	87	64	515278	1.73	12	13	8.36	7 + 2 part	95.992	21	23	23	24	176155	155257	162238	150636	11710452	11667067	11481145	10298219
	700000	0.47	219	166	111	85	65	514933	1.83	10	11	9.11	7 + 2 part	96.028	21	23	22	24	174330	157680	163424	151593	11709813	11672075	11497547	10435364
	800000	0.38	214	162	109	84	66	514989	1.7	10	11	8.56	7 + 2 part	96.015	20	22	22	23	177323	162111	174331	157680	11704740	11668408	11500057	10534700
	900000	0.81	220	165	112	86	68	553149	1.67	11	11	7.83	8 + 0 part	96.015	21	22	23	24	162237	155244	154501	135426	11707840	11669131	11504737	10499507
	1000000	0.47	216	162	107	84	67	533223	1.65	11	12	8.66	7 + 3 part	96.042	21	22	22	23	176940	162110	166790	155244	11713507	11676048	11507897	10583060
	1200000	0.55	220	161	104	80	64	552580	1.66	11	11	7.73	11 + 2 part	96.043	20	20	21	22	177433	177433	176940	162111	11712409	11670926	11498306	10602185
	1400000	0.38	223	164	108	83	67	552580	1.62	9	9	7.77	11 + 1 part	96.047	21	22	22	23	168378	162111	162238	155261	11712862	11671229	11508145	10584339
	1600000	0.47	217	160	106	83	67	552579	1.84	8	8	7.7	12 + 1 part	96.002	21	22	22	23	168391	162360	162487	156641	11708767	11668762	11511717	10602520
	2000000	0.56	214	159	103	81	65	552579	1.64	10	10	6.79	14 + 1 part	96.008	20	21	21	22	177479	168285	176940	162360	11709716	11670665	11504771	10628385
	2500000	0.47	217	157	101	80	66	552579	1.8	8	8	7.58	15 + 1 part	96.014	20	20	21	22	177479	177479	176940	174318	11712421	11669841	11503714	10719345
	3000000	0.47	217	157	101	80	66	552579	1.8	8	8	7.58	15 + 1 part	96.014	20	20	21	22	177479	177479	176940	174318	11712421	11669841	11503714	10719345
	3500000	0.47	217	157	101	80	66	552579	1.8	8	8	7.58	15 + 1 part	96.014	20	20	21	22	177479	177479	176940	174318	11712421	11669841	11503714	10719345

Supplementary Table 5: Samtools variant analysis against the S288C reference genome. Columns 1 and 2 detail the position of the variant. Columns 3 and 4 note the reference allele (Genotype 0) and the alternative allele (Genotype 1). Columns 5-7 and 8-10 denote the called genotype and the likelihood for each of the two genotypes for strains 1691 and 1693 respectively. Likelihoods were calculated from the reported Phread-scaled Genotype Likelihoods, with the maximum likelihood being 1. Columns 11-13 report the location of the variant and its effect. For the sake of brevity, those variants which are shared between 1691 and 1693 are not shown. The most likely discordant variants (those which have a likelihood of 1 for one genotype and <0.00 for the other) are shaded. Each of these was also called by GATK (see supplemenary table 6). Clear discordand SNPs that could be confirmed by aligning the reads to IGV are shaded dark grey (these all had a high likelihood).

				1691			1693					
Chrom	POS	Genotype 0	Genotype 1	Genoype (G)	Likelihood G=0	Likelihood G=1	Genotype (G)	Likelihood G=0	Likelihood G=1	Annotation	Gene	Gene ID
I	26391	A	G	0	0.37	0.17	1	0.14	0.38	synonymous_variant	FLO9	YAL063C
I	26940	A	G	0	0.05	0.03	1	0.01	0.02	synonymous_variant	FLO9	YAL063C
I	26974	A	G	0	0.04	0.03	1	0.01	0.04	missense_variant	FLO9	YAL063C
I	204783	C	G	0	1.00	0.85	1	0.35	1.00	missense_variant	FLO1	YAR050W
I	204796	A	G	0	1.00	0.85	1	0.26	1.00	missense_variant	FLO1	YAR050W
IV	827861	G	T	0	1.00	0.00	1	0.00	1.00	missense_variant	CDC1	YDR182W
IV	1308011	T	C	0	0.78	0.48	1	0.10	1.00	missense_variant	HKR1	YDR420W
V	205872	G	A	1	0.00	1.00	0	1.00	0.00	missense_variant	GCD11	YER025W
V	362749	A	T	0	1.00	0.00	1	0.00	1.00	upstream_gene_variant	PRS2	YER099C
VI	4717	C	G	0	1.00	0.00	1	0.01	0.17	upstream_gene_variant	YFL066C	YFL066C
VIII	499958	C	T	0	1.00	0.00	1	0.00	1.00	missense_variant	PPX1	YHR201C
VIII	511255	C	T	0	1.00	0.00	1	0.00	1.00	synonymous_variant	SCH9	YHR205W
VIII	514829	CTATATATATATATATATATATATATATATA	CTATATATATATATATATATATATATATATATA	1	0.00	0.03	0	0.11	0.00	upstream_gene_variant	BAT1	YHR208W
XI	59567	T	G	0	1.00	0.00	1	0.00	1.00	missense_variant	TOR2	YKL203C
XI	541138	G	T	1	0.00	1.00	0	1.00	0.00	missense_variant	DYN1	YKR054C
XII	734703	T	A	1	0.00	1.00	0	1.00	0.00	upstream_gene_variant	YLR302C	YLR302C
XIV	189585	C	T	1	0.00	1.00	0	1.00	0.00	missense_variant	SLA2	YNL243W
XIV	415070	T	TTC	0	1.00	0.00	1	0.02	0.40	upstream_gene_variant	YNL115C	YNL115C
XVI	667520	T	G	0	1.00	0.00	1	0.00	1.00	upstream_gene_variant	ATG11	YPR049C

Supplementary Table 6: GATK variant calling against reference S288C. Columns 1 and 2 detail the position of the variant. Columns 3 and 4 note the reference allele (Genotype 0) and the alternative alleles (Genotypes 1 and 2). Colums 5-6 and 7-8 report the genotype called and the confidence associated with the call for strain 1691 and 1693 respectivly (confidence is out of 100). Blank rows indicate that a genotype was not called for that strain. Columns 9-11 report the location of the variant and its effect. For the sake of brevity, those variants which are shared between 1691 and 1693 are not shown. Discordant variants with high confidence (99 for both calls) are shaded in light grey. Variants that were also called by Samtools (see supplementary table 5) are shaded in dark grey (each of these was also high confidence).

				strain 1691		strain 1693				
CHROM	POS	Genotype 0	Genotype 1, 2	Genotype	Confidence	Genotype	Confidence	Annotation	Gene	Gene ID
I	65780	A	AACCGATGAATTCGAGCTCGTTTTCGACA CTGGATGGCGGCGTTAGTATCGAATCGAC AGCAGTATAGCGACCAGCATTCACATACG ATTGACGCATGATATTACTTTCTGCGCACT TAACTTCGCATCTGGGCAGATGATGTCGA G	0	45	1	99	frameshift_variant&stop_gained	CLN3	YAL040C
I	206176	CG	C	0	99	1	99	frameshift_variant	FLO1	YAR050W
I	206179	A	AC			1	99	frameshift_variant	FLO1	YAR050W
III	148614	T	TTGTTGGAATAAAAATCAACTATCATCTAC TAACTAGTATTTA	1	99			stop_gained&conservative_inframe_insertion	SRD1	YCR018C
III	148617	T	TTACTAGTATATTATCATATACGGTGTTAG AAGATGACGCAAATGATGAGAAATAG	1	99			frameshift_variant&stop_gained	SRD1	YCR018C
IV	1525361	GGCTTCTCTCT	G	0	99	1	99	frameshift_variant	YDR544C	YDR544C
II	68282	G	GTTATTATTATTATTATTATTATTATTATTA	0	99	1	99	disruptive_inframe_insertion	CDC27	YBL084C
IV	827861	G	T	0	99	1	99	missense_variant	CDC1	YDR182W
IV	1160444	C	T			1	64	missense_variant	HXT6	YDR343C
IV	1525313	T	G			1	99	missense_variant	YDR544C	YDR544C
IV	1525335	C	G			1	99	missense_variant	YDR544C	YDR544C
IV	1525340	C	A			1	99	missense_variant	YDR544C	YDR544C
IV	1525345	C	G			1	99	missense_variant	YDR544C	YDR544C
IV	1525351	C	G			1	99	missense_variant	YDR544C	YDR544C
IV	1525357	C	G			1	99	missense_variant	YDR544C	YDR544C
IX	515	A	ATAG	1	90	0	99	conservative_inframe_insertion	YIL177C	YIL177C
IX	521	A	T	1	90	0	99	missense_variant	YIL177C	YIL177C
V	205872	G	A	1	99	0	99	missense_variant	GCD11	YER025W
VIII	1846	A	AGTAGTAGCACTAGTCCTGACGTTGGTGC TGGCAGTGGTAGTAGCATTAGTGCTGGAG TTGGTACTTTCAGTGGTAGTCGCACTAGT GTTGGAGTTGGTACTTTCAGTGGTAGTCG CACTAGTCCTGACGTTGATGCTGGCAGTG			1	99	conservative_inframe_insertion	YHL050C	YHL050C
VIII	289314	T	G	1	60	0	99	missense_variant	AHT1	YHR093W
VIII	499958	C	T	0	99	1	99	missense_variant	PPX1	YHR201C
XI	59567	T	G	0	99	1	99	missense_variant	TOR2	YKL203C
XI	541138	G	T	1	99	0	99	missense_variant	DYN1	YKR054C
XIV	189585	C	T	1	99	0	99	missense_variant	SLA2	YNL243W
XV	708819	G	C	1	99	0	99	missense_variant	YOR192C-B	YOR192C-B
I	206126	T	C			1	99	synonymous_variant	FLO1	YAR050W
I	206129	A	C			1	99	synonymous_variant	FLO1	YAR050W
I	206132	G	T			1	99	synonymous_variant	FLO1	YAR050W
I	206144	C	T			1	99	synonymous_variant	FLO1	YAR050W
IV	1160352	C	T			1	99	synonymous_variant	HXT6	YDR343C
VIII	511255	C	T	0	99	1	99	synonymous_variant	SCH9	YHR205W
XII	944060	G	A			1	99	synonymous_variant	YLR410W-B	YLR410W-B
XII	944099	A	C			1	84	synonymous_variant	YLR410W-B	YLR410W-B
XV	708878	T	C	1	99	0	99	synonymous_variant	YOR192C-B	YOR192C-B
I	101281	C	CA			1	99	upstream_gene_variant	DRS2	YAL026C
I	223127	C	CTTTTTTTTTTTTTTTTTTTT , CTTTTTTTTTTTTTTTTTTT	2	99	1	99	upstream_gene_variant	PHO11	YAR071W
II	9022	G	GA	0	99	1	99	upstream_gene_variant	YBL111C	YBL111C
II	165902	C	CT			1	59	upstream_gene_variant	PET9	YBL030C
III	84780	A	T			1	99	upstream_gene_variant	YCL022C	YCL022C
IV	13937	G	A	0	52	1	99	upstream_gene_variant	SOR2	YDL246C
IV	126537	C	CTTTTTTTTTTTT	1	99			upstream_gene_variant	UFD2	YDL190C
IV	678013	C	CTT , CTTTT	1	99	2	64	upstream_gene_variant	YDR109C	YDR109C
IV	892698	G	GTTTTTTT , GTTTT	2	99	1	99	upstream_gene_variant	AHA1	YDR214W
IX	163245	AT	A	1	73	0	99	upstream_gene_variant	SEC24	YIL109C
IX	187822	GA	G	1	78	0	99	upstream_gene_variant	BMT5	YIL096C
IX	241032	G	GA	0	99	1	99	upstream_gene_variant	YIL067C	YIL067C
IX	339225	C	CT	0	99	1	87	upstream_gene_variant	EST3	YIL009C-A
IX	439814	AGTGTGGTGTGTGGG TGTGG	A	1	99	0	99	upstream_gene_variant	YIR042C	YIR042C
V	362749	A	T	0	99	1	99	upstream_gene_variant	PRS2	YER099C
VII	555981	C	CAAAA	1	99	0	99	upstream_gene_variant	TIM21	YGR033C
VIII	340058	TTA	T	1	70	0	99	upstream_gene_variant	YHR112C	YHR112C
VIII	514829	C	CTA	1	99	0	99	upstream_gene_variant	BAT1	YHR208W
VIII	549762	C	CTTTTTTTT , CTTTTTTTTTTTTTTTTTTT	2	99	1	99	upstream_gene_variant	YHR214C-B	YHR214C-B
XI	666614	TGTGTGGGTGTGGTG TGGGTGTGGTGTGGG TGTGGGTGTG	T	0	99	1	99	downstream_gene_variant	GEX2	YKR106W
XI	666740	T	G	0	99	1	90	downstream_gene_variant	GEX2	YKR106W
XI	666742	G	T	0	99	1	90	downstream_gene_variant	GEX2	YKR106W
XII	48855	C	CAAAAAAAAAAAAA	1	99	0	99	upstream_gene_variant	YBT1	YLL048C
XII	468217	A	G			1	38	upstream_gene_variant	RDN25-2	RDN25-2
XII	585920	GA	G	1	99	0	99	upstream_gene_variant	UTP13	YLR222C
XII	704138	TA	T	1	61	0	99	upstream_gene_variant	YSH1	YLR277C
XII	734703	T	A	1	99	0	99	upstream_gene_variant	YLR302C	YLR302C
XIII	5924	A	C			1	52	upstream_gene_variant	YML133C	YML133C
XIII	483743	GA	G			1	50	upstream_gene_variant	YKU80	YMR106C
XIV	154923	GA	G	0	99	1	99	upstream_gene_variant	ORC5	YNL261W
XIV	413448	C	CT			1	96	upstream_gene_variant	YNL115C	YNL115C
XIV	415054	C	CTT			1	99	upstream_gene_variant	YNL115C	YNL115C
XIV	415071	C	T			1	99	upstream_gene_variant	YNL115C	YNL115C
XIV	415088	C	T			1	99	upstream_gene_variant	YNL115C	YNL115C
XIV	415096	C	T			1	99	upstream_gene_variant	YNL115C	YNL115C
XIV	479459	AT	A	1	61	0	47	upstream_gene_variant	SWS2	YNL081C
XVI	667520	T	G	0	99	1	99	upstream_gene_variant	ATG11	YPR049C

Supplementary Table 7: Samtools variant analysis against the BY4741 draft genome. Columns 1 and 2 detail the position of the variant. Columns 3 and 4 note the reference allele (Genotype 0) and the alternative allele (Genotype 1). Columns 5-7 and 8-10 denote the called genotype and the likelihood for each of the two genotypes for strains 1691 and 1693 respectively. Likelihoods were calculated from the reported Phread-scaled Genotype Likelihoods, with the maximum likelihood being 1. Columns 11-13 report the location of the variant and its effect. For the sake of brevity, those variants which are shared between 1691 and 1693 and 62 variants called in intergenic regions are not shown. The most likely discordant variants (those which have a likelihood of 1 for one genotype and <0.00 for the other) are shaded light grey. Those variants which were also identified by GATK (supplemental table 8) are shaded in dark grey (these were also high likelihood).

				1691			1693					
Scaffold (Genbank ID)	POS	Genotype 0	Genotype 1	Genoype (G)	Likelihood G=0	Likelihood G=1	Genotype (G)	Likelihood G=0	Likelihood G=1	Annotation	Gene	Gene ID
gi_696447945_gb_JRIS01000005.1_	22253	G	T	0	1.00	0.00	1	0.00	1.00	missense_variant	CDC1	YDR182W
gi_696447920_gb_JRIS01000013.1_	87004	C	T	1	0.00	1.00	0	1.00	0.00	missense_variant	SLA2	YNL243W
gi_696447894_gb_JRIS01000021.1_	99	G	A	0	1.00	0.91	1	0.19	1.00	upstream_gene_variant		UNDEF10
gi_696447894_gb_JRIS01000021.1_	6781	T	C	1	0.00	1.00	0	1.00	0.00	synonymous_variant		UNDEF12
gi_696447894_gb_JRIS01000021.1_	10355	CTATATATATATATATATATATATATATA	CTATATATATATATATATATATATATATATA	1	0.00	0.02	0	0.11	0.00	upstream_gene_variant		UNDEF18
gi_696447858_gb_JRIS01000030.1_	83846	T	A	0	1.00	0.00	1	0.00	1.00	downstream_gene_variant	STT4	YLR305C
gi_696447821_gb_JRIS01000042.1_	106687	G	T	1	0.00	1.00	0	1.00	0.00	upstream_gene_variant	ATG11	YPR049C
gi_696447795_gb_JRIS01000048.1_	48923	CTATATATATATATATATATATATATATATA	CTATATATATATATATATATATATATATA	1	0.01	0.02	0	0.17	0.00	upstream_gene_variant	TOM71	YHR117W
gi_696447774_gb_JRIS01000053.1_	29830	A	G	0	1.00	0.76	1	0.17	1.00	upstream_gene_variant	PHO87	YCR037C
gi_696447735_gb_JRIS01000063.1_	32496	TTATTATTNNNNNNNNNNNNNNNNNNNNNNNNNNNTATTATT	TTATTATT	1	0.00	0.02	0	1.00	0.00	frameshift_variant		UNDEF61
gi_696447735_gb_JRIS01000063.1_	32502	TTNNNNNNNNNNNNNNNNNNNNNNNNNNT	TTATTATTATTATTATTATNNNNNNNNNNNNNNNNNNNNNNNNNT	1	0.00	0.10	0	0.56	0.00	frameshift_variant&stop_gained		UNDEF61
gi_696447735_gb_JRIS01000063.1_	130139	CTC	CTCTTTTTTTTTTTTTTTTTTTTTTTTTTC	0	0.76	0.04	1	0.01	0.09	upstream_gene_variant	PET9	YBL030C
gi_696447727_gb_JRIS01000065.1_	83619	ATAATAATAATAATAATAATAATAATAATNNNNNNNNNNNNNNNNNNNNNNNNNNNNNNNNNNNNNNNNNNNNTAATAATAATAATAATAATAATAATAAT	ATAATAATAATAATAATAATAATAATAAT	1	0.00	0.01	0	0.00	0.00	upstream_gene_variant	FKH1	YIL131C
gi_696447727_gb_JRIS01000065.1_	83622	ATAATAATAATAATAATAATAATAATNNNNNNNNNNNNNNNNNNNNNNNNNNNNNNNNNNNNNNNNNNNNTAATAATAATAATAATAATAATAAT	ATAATAATAATAATAATAATAATAAT	1	0.00	0.01	0	0.00	0.00	upstream_gene_variant	FKH1	YIL131C
gi_696447727_gb_JRIS01000065.1_	83625	ATAATAATAATAATAATAATAATNNNNNNNNNNNNNNNNNNNNNNNNNNNNNNNNNNNNNNNNNNNNNTAATAATAATAATAATAATAAT	ATAATAATAATAATAATAATAAT	1	0.00	0.01	0	0.00	0.00	upstream_gene_variant	FKH1	YIL131C
gi_696447727_gb_JRIS01000065.1_	116337	ACCCCCCCCC	ACCCCCCCCCCCCCC	0	0.06	0.04	1	0.01	0.03	upstream_gene_variant	RPI1	YIL119C
gi_696447695_gb_JRIS01000074.1_	1172	C	T	0	0.02	0.01	1	0.00	0.01	missense_variant&splice_region_variant	YNL019C	YNL019C
gi_696447659_gb_JRIS01000084.1_	56773	T	G	0	1.00	0.00	1	0.00	1.00	missense_variant	TOR2	YKL203C
gi_696447656_gb_JRIS01000085.1_	20647	C	T	0	0.32	0.20	1	0.04	0.32	synonymous_variant	CDC4	YFL009W
gi_696447656_gb_JRIS01000085.1_	20650	G	A	0	0.35	0.15	1	0.04	0.33	synonymous_variant	CDC4	YFL009W
gi_696447656_gb_JRIS01000085.1_	20656	T	C	0	0.37	0.10	1	0.04	0.15	synonymous_variant	CDC4	YFL009W
gi_696447656_gb_JRIS01000085.1_	20658	G	A	0	0.33	0.10	1	0.04	0.13	synonymous_variant	CDC4	YFL009W
gi_696447656_gb_JRIS01000085.1_	20691	A	G	0	0.12	0.06	1	0.03	0.12	synonymous_variant	CDC4	YFL009W
gi_696447656_gb_JRIS01000085.1_	20715	T	C	0	0.12	0.04	1	0.71	0.10	missense_variant	CDC4	YFL009W
gi_696447656_gb_JRIS01000085.1_	20717	G	A	0	0.17	0.04	1	0.04	0.10	missense_variant	CDC4	YFL009W
gi_696447656_gb_JRIS01000085.1_	20751	T	A	0	0.08	0.04	1	0.02	0.11	missense_variant	CDC4	YFL009W
gi_696447656_gb_JRIS01000085.1_	20752	C	T	0	0.07	0.03	1	0.02	0.11	synonymous_variant	CDC4	YFL009W
gi_696447656_gb_JRIS01000085.1_	20756	G	A	0	0.07	0.04	1	0.02	0.11	missense_variant	CDC4	YFL009W
gi_696447646_gb_JRIS01000088.1_	46918	G	A	1	0.32	1.00	0	1.00	0.91	upstream_gene_variant	CBF2	YGR140W
gi_696447632_gb_JRIS01000092.1_	1848	A	G	1	0.40	1.00	0	1.00	0.91	downstream_gene_variant	YNR066C	YNR066C
gi_696447626_gb_JRIS01000094.1_	17777	TTTNNNNNNNNNNNNNNNNNNN	T	1	0.10	0.54	0	1.00	0.17	upstream_gene_variant	RPA34	YJL148W
gi_696447615_gb_JRIS01000097.1_	24314	TCCCCC	TCCCCCCCCCCCC	1	0.05	0.13	0	0.93	0.15	upstream_gene_variant	DMC1	YER179W
gi_696447601_gb_JRIS01000101.1_	48	T	C	0	1.00	0.00	1	0.04	0.25	upstream_gene_variant		UNDEF199
gi_696447592_gb_JRIS01000103.1_	68377	T	A	0	1.00	0.00	1	0.00	1.00	upstream_gene_variant	RTT105	YER104W
gi_696447592_gb_JRIS01000103.1_	224570	C	T	1	0.00	1.00	0	1.00	0.00	missense_variant	GCD11	YER025W
gi_696447536_gb_JRIS01000118.1_	15018	C	A	1	0.00	1.00	0	1.00	0.00	missense_variant	DYN1	YKR054C
gi_696447526_gb_JRIS01000121.1_	77	G	A	0	0.02	0.01	1	0.00	0.03	upstream_gene_variant	YOL166C	YOL166C
gi_696447526_gb_JRIS01000121.1_	95	G	A	0	0.02	0.01	1	0.00	0.01	upstream_gene_variant	YOL166C	YOL166C
gi_696447464_gb_JRIS01000138.1_	81304	T	TAATTTGAAAAAAAAAAAAAAAAAAAAAAAAAACTAG	0	0.81	0.17	1	0.00	0.05	upstream_gene_variant	RRG7	YOR305W
gi_696447394_gb_JRIS01000158.1_	454036	C	G	0	0.19	0.05	1	0.13	0.35	upstream_gene_variant	ITR2	YOL103W
gi_696447304_gb_JRIS01000182.1_	177	A	G	1	0.25	0.65	0	1.00	0.06	synonymous_variant	SSB1	YDL229W
gi_696447304_gb_JRIS01000182.1_	192	G	A	1	0.22	0.69	0	1.00	0.08	synonymous_variant	SSB1	YDL229W
gi_696447304_gb_JRIS01000182.1_	209	A	G	1	0.23	0.66	0	0.85	0.07	synonymous_variant	SSB1	YDL229W
gi_696447304_gb_JRIS01000182.1_	234	G	A	1	0.11	0.25	0	0.28	0.06	synonymous_variant	SSB1	YDL229W
gi_696447304_gb_JRIS01000182.1_	321	A	G	1	0.05	0.11	0	0.14	0.04	synonymous_variant	SSB1	YDL229W
gi_696447304_gb_JRIS01000182.1_	489	G	A	0	0.02	0.02	1	0.02	0.05	synonymous_variant	SSB1	YDL229W
gi_696447304_gb_JRIS01000182.1_	579	A	T	1	0.00	0.01	0	0.00	0.00	synonymous_variant	SSB1	YDL229W
gi_696447300_gb_JRIS01000183.1_	5253	A	G	1	0.00	1.00	0	1.00	0.00	missense_variant	PPX1	YHR201C
gi_696447292_gb_JRIS01000185.1_	3281	G	A	0	1.00	0.91	1	0.04	1.00	upstream_gene_variant	YPS5	YGL259W
gi_696447292_gb_JRIS01000185.1_	3292	C	T	0	1.00	0.91	1	0.08	1.00	upstream_gene_variant	YPS5	YGL259W
gi_696447257_gb_JRIS01000194.1_	24723	A	G	0	1.00	0.93	1	0.21	1.00	synonymous_variant	YNR065C	YNR065C
gi_696447249_gb_JRIS01000196.1_	187624	G	GAAAAAAAAAAAAAAAAAAAAAAAAAAA	0	0.07	0.03	1	0.00	0.06	upstream_gene_variant	SPC3	YLR066W
gi_696447197_gb_JRIS01000210.1_	103	T	G	0	1.00	0.03	1	0.05	0.16	upstream_gene_variant	YHL034C	YHL034C
gi_696447193_gb_JRIS01000211.1_	8034	AAANNNNNNNNNNNNNNNNNNNNNNN	A	0	1.00	0.26	1	0.14	1.00	upstream_gene_variant	VMA1	YDL185W
gi_696447136_gb_JRIS01000226.1_	7	C	T	0	0.87	0.08	1	0.07	1.00	upstream_gene_variant	ARN2	YHL047C
gi_696446798_gb_JRIS01000321.1_	4918	C	A	0	0.02	0.01	1	0.02	0.07	upstream_gene_variant	PHO12	YHR215W
gi_696446726_gb_JRIS01000339.1_	84	C	G	0	0.05	0.04	1	0.09	0.30	upstream_gene_variant	YMR326C	YMR326C
gi_696446714_gb_JRIS01000342.1_	401	T	C	0	0.13	0.06	1	0.04	0.11	upstream_gene_variant	MAL12	YGR292W
gi_696446714_gb_JRIS01000342.1_	406	C	T	0	0.12	0.06	1	0.04	0.11	upstream_gene_variant	MAL12	YGR292W

Supplementary Table 8: GATK variant calling against the BY4741 draft genome. Columns 1 and 2 detail the position of the variant. Columns 3 and 4 note the reference allele (Genotype 0) and the alternative alleles (Genotypes 1 and 2). Colums 5-6 and 7-8 report the genotype called and the confidence associated with the call for strain 1691 and 1693 respectivly (confidence is out of 100). Blank rows indicate that a genotype was not called for that strain. Columns 9-11 report the location of the variant and its effect. For the sake of brevity, those variants which are shared between 1691 and 1693 and 226 variants called in intergenic regions are not shown. Discordant variants with high confidence (99 for both calls) are shaded in light grey. Variants that were also called by Samtools (see supplementary table 7) are shaded in dark grey (each of these was also high confidence).

<table>
<tr><th></th><th></th><th></th><th></th><th colspan="2">1691</th><th colspan="2">1693</th><th></th><th></th><th></th></tr>
<tr><th>Scaffold Genbank ID</th><th>POS</th><th>Genotype 0</th><th>Genotype 1, 2</th><th>Genotype</th><th>Confidence</th><th>Genotype</th><th>Confidence</th><th>Annotation</th><th>Gene</th><th>Gene ID</th></tr>
<tr><td>gi|696447945|gb|JRIS01000005.1|</td><td>22253</td><td>G</td><td>T</td><td>0</td><td>99</td><td>1</td><td>99</td><td>missense_variant</td><td>CDC1</td><td>YDR182W</td></tr>
<tr><td>gi|696447920|gb|JRIS01000013.1|</td><td>36866</td><td>T</td><td>A</td><td>0</td><td>99</td><td>1</td><td>99</td><td>synonymous_variant</td><td>LYP1</td><td>YNL268W</td></tr>
<tr><td>gi|696447920|gb|JRIS01000013.1|</td><td>36872</td><td>T</td><td>C</td><td>0</td><td>99</td><td>1</td><td>99</td><td>synonymous_variant</td><td>LYP1</td><td>YNL268W</td></tr>
<tr><td>gi|696447920|gb|JRIS01000013.1|</td><td>36889</td><td>A</td><td>G</td><td></td><td></td><td>1</td><td>99</td><td>missense_variant</td><td>LYP1</td><td>YNL268W</td></tr>
<tr><td>gi|696447920|gb|JRIS01000013.1|</td><td>36893</td><td>G</td><td>A</td><td></td><td></td><td>1</td><td>99</td><td>synonymous_variant</td><td>LYP1</td><td>YNL268W</td></tr>
<tr><td>gi|696447920|gb|JRIS01000013.1|</td><td>87004</td><td>C</td><td>T</td><td>1</td><td>99</td><td>0</td><td>99</td><td>missense_variant</td><td>SLA2</td><td>YNL243W</td></tr>
<tr><td>gi|696447894|gb|JRIS01000021.1|</td><td>99</td><td>G</td><td>A</td><td></td><td></td><td>1</td><td>86</td><td>upstream_gene_variant</td><td></td><td>UNDEF10</td></tr>
<tr><td>gi|696447894|gb|JRIS01000021.1|</td><td>162</td><td>T</td><td>C</td><td></td><td></td><td>1</td><td>99</td><td>upstream_gene_variant</td><td></td><td>UNDEF10</td></tr>
<tr><td>gi|696447894|gb|JRIS01000021.1|</td><td>6781</td><td>T</td><td>C</td><td>1</td><td>99</td><td>0</td><td>99</td><td>synonymous_variant</td><td></td><td>UNDEF12</td></tr>
<tr><td>gi|696447894|gb|JRIS01000021.1|</td><td>10355</td><td>C</td><td>CTA</td><td>1</td><td>99</td><td>0</td><td>99</td><td>upstream_gene_variant</td><td></td><td>UNDEF18</td></tr>
<tr><td>gi|696447881|gb|JRIS01000023.1|</td><td>10511</td><td>A</td><td>G</td><td></td><td></td><td>1</td><td>99</td><td>synonymous_variant</td><td>PAU5</td><td>YFL020C</td></tr>
<tr><td>gi|696447881|gb|JRIS01000023.1|</td><td>10517</td><td>T</td><td>A</td><td></td><td></td><td>1</td><td>99</td><td>synonymous_variant</td><td>PAU5</td><td>YFL020C</td></tr>
<tr><td>gi|696447881|gb|JRIS01000023.1|</td><td>10522</td><td>T</td><td>C</td><td></td><td></td><td>1</td><td>99</td><td>missense_variant</td><td>PAU5</td><td>YFL020C</td></tr>
<tr><td>gi|696447881|gb|JRIS01000023.1|</td><td>10532</td><td>A</td><td>G</td><td></td><td></td><td>1</td><td>99</td><td>synonymous_variant</td><td>PAU5</td><td>YFL020C</td></tr>
<tr><td>gi|696447881|gb|JRIS01000023.1|</td><td>10535</td><td>A</td><td>G</td><td></td><td></td><td>1</td><td>99</td><td>synonymous_variant</td><td>PAU5</td><td>YFL020C</td></tr>
<tr><td>gi|696447881|gb|JRIS01000023.1|</td><td>10550</td><td>G</td><td>A</td><td>1</td><td>99</td><td>0</td><td>99</td><td>synonymous_variant</td><td>PAU5</td><td>YFL020C</td></tr>
<tr><td>gi|696447881|gb|JRIS01000023.1|</td><td>10553</td><td>A</td><td>G</td><td></td><td></td><td>1</td><td>99</td><td>synonymous_variant</td><td>PAU5</td><td>YFL020C</td></tr>
<tr><td>gi|696447881|gb|JRIS01000023.1|</td><td>10558</td><td>G</td><td>A</td><td>1</td><td>99</td><td>0</td><td>99</td><td>stop_gained</td><td>PAU5</td><td>YFL020C</td></tr>
<tr><td>gi|696447881|gb|JRIS01000023.1|</td><td>10562</td><td>G</td><td>C,A</td><td>2</td><td>99</td><td>1</td><td>99</td><td>synonymous_variant</td><td>PAU5</td><td>YFL020C</td></tr>
<tr><td>gi|696447881|gb|JRIS01000023.1|</td><td>10563</td><td>G</td><td>A</td><td></td><td></td><td>1</td><td>99</td><td>missense_variant</td><td>PAU5</td><td>YFL020C</td></tr>
<tr><td>gi|696447881|gb|JRIS01000023.1|</td><td>10564</td><td>A</td><td>T</td><td></td><td></td><td>1</td><td>99</td><td>missense_variant</td><td>PAU5</td><td>YFL020C</td></tr>
<tr><td>gi|696447881|gb|JRIS01000023.1|</td><td>10573</td><td>C</td><td>G</td><td></td><td></td><td>1</td><td>99</td><td>missense_variant</td><td>PAU5</td><td>YFL020C</td></tr>
<tr><td>gi|696447881|gb|JRIS01000023.1|</td><td>10574</td><td>A</td><td>G</td><td></td><td></td><td>1</td><td>99</td><td>synonymous_variant</td><td>PAU5</td><td>YFL020C</td></tr>
<tr><td>gi|696447881|gb|JRIS01000023.1|</td><td>10580</td><td>A</td><td>G</td><td></td><td></td><td>1</td><td>99</td><td>synonymous_variant</td><td>PAU5</td><td>YFL020C</td></tr>
<tr><td>gi|696447881|gb|JRIS01000023.1|</td><td>10595</td><td>G</td><td>A</td><td></td><td></td><td>1</td><td>99</td><td>synonymous_variant</td><td>PAU5</td><td>YFL020C</td></tr>
<tr><td>gi|696447881|gb|JRIS01000023.1|</td><td>10598</td><td>A</td><td>G</td><td></td><td></td><td>1</td><td>99</td><td>synonymous_variant</td><td>PAU5</td><td>YFL020C</td></tr>
<tr><td>gi|696447881|gb|JRIS01000023.1|</td><td>10601</td><td>A</td><td>G</td><td></td><td></td><td>1</td><td>99</td><td>synonymous_variant</td><td>PAU5</td><td>YFL020C</td></tr>
<tr><td>gi|696447881|gb|JRIS01000023.1|</td><td>10604</td><td>G</td><td>A</td><td>1</td><td>99</td><td>0</td><td>99</td><td>synonymous_variant</td><td>PAU5</td><td>YFL020C</td></tr>
<tr><td>gi|696447875|gb|JRIS01000025.1|</td><td>49526</td><td>C</td><td>T</td><td></td><td></td><td>1</td><td>99</td><td>missense_variant</td><td>RPL2B</td><td>YIL018W</td></tr>
<tr><td>gi|696447875|gb|JRIS01000025.1|</td><td>49532</td><td>T</td><td>C</td><td></td><td></td><td>1</td><td>99</td><td>missense_variant</td><td>RPL2B</td><td>YIL018W</td></tr>
<tr><td>gi|696447875|gb|JRIS01000025.1|</td><td>49544</td><td>A</td><td>G</td><td></td><td></td><td>1</td><td>99</td><td>missense_variant</td><td>RPL2B</td><td>YIL018W</td></tr>
<tr><td>gi|696447858|gb|JRIS01000030.1|</td><td>83846</td><td>T</td><td>A</td><td>0</td><td>99</td><td>1</td><td>99</td><td>downstream_gene_variant</td><td>STT4</td><td>YLR305C</td></tr>
<tr><td>gi|696447858|gb|JRIS01000030.1|</td><td>83863</td><td>G</td><td>GACGAACAAAGACACTGTTGGAATAAAAATCCACTAT
TGTCTATCAACTAATACTTATACTATCAATATATTATAA
TAGACGGCGTTAGATGATGACATAAGTTATGAGAAG
ATGTCATCGAGGTTA</td><td>0</td><td>99</td><td>1</td><td>99</td><td>downstream_gene_variant</td><td>STT4</td><td>YLR305C</td></tr>
<tr><td>gi|696447847|gb|JRIS01000034.1|</td><td>898</td><td>A</td><td>T</td><td></td><td></td><td>1</td><td>99</td><td>upstream_gene_variant</td><td>YAL065C</td><td>YAL065C</td></tr>
<tr><td>gi|696447821|gb|JRIS01000042.1|</td><td>106687</td><td>G</td><td>T</td><td>1</td><td>99</td><td>0</td><td>99</td><td>upstream_gene_variant</td><td>ATG11</td><td>YPR049C</td></tr>
<tr><td>gi|696447821|gb|JRIS01000042.1|</td><td>140522</td><td>C</td><td>T</td><td></td><td></td><td>1</td><td>99</td><td>synonymous_variant</td><td>TEF1</td><td>YPR080W</td></tr>
<tr><td>gi|696447799|gb|JRIS01000047.1|</td><td>950</td><td>C</td><td>T</td><td></td><td></td><td>1</td><td>90</td><td>synonymous_variant</td><td>HSP82</td><td>YPL240C</td></tr>
<tr><td>gi|696447799|gb|JRIS01000047.1|</td><td>971</td><td>C</td><td>T</td><td>0</td><td>43</td><td>1</td><td>90</td><td>synonymous_variant</td><td>HSP82</td><td>YPL240C</td></tr>
<tr><td>gi|696447795|gb|JRIS01000048.1|</td><td>99699</td><td>A</td><td>C</td><td>1</td><td>99</td><td>0</td><td>99</td><td>missense_variant</td><td>AHT1</td><td>YHR093W</td></tr>
<tr><td>gi|696447795|gb|JRIS01000048.1|</td><td>158738</td><td>G</td><td>A</td><td></td><td></td><td>1</td><td>99</td><td>missense_variant</td><td></td><td>UNDEF44</td></tr>
<tr><td>gi|696447795|gb|JRIS01000048.1|</td><td>158740</td><td>A</td><td>G</td><td></td><td></td><td>1</td><td>99</td><td>synonymous_variant</td><td></td><td>UNDEF44</td></tr>
<tr><td>gi|696447795|gb|JRIS01000048.1|</td><td>158785</td><td>T</td><td>A</td><td>1</td><td>99</td><td>0</td><td>99</td><td>synonymous_variant</td><td></td><td>UNDEF44</td></tr>
<tr><td>gi|696447795|gb|JRIS01000048.1|</td><td>158786</td><td>G</td><td>A</td><td>1</td><td>99</td><td>0</td><td>99</td><td>missense_variant</td><td></td><td>UNDEF44</td></tr>
<tr><td>gi|696447795|gb|JRIS01000048.1|</td><td>158789</td><td>T</td><td>C</td><td>1</td><td>99</td><td>0</td><td>99</td><td>missense_variant</td><td></td><td>UNDEF44</td></tr>
<tr><td>gi|696447795|gb|JRIS01000048.1|</td><td>158800</td><td>C</td><td>T</td><td>1</td><td>45</td><td>0</td><td>99</td><td>synonymous_variant</td><td></td><td>UNDEF44</td></tr>
<tr><td>gi|696447795|gb|JRIS01000048.1|</td><td>158805</td><td>T</td><td>C</td><td>1</td><td>45</td><td>0</td><td>99</td><td>missense_variant</td><td></td><td>UNDEF44</td></tr>
<tr><td>gi|696447795|gb|JRIS01000048.1|</td><td>158806</td><td>A</td><td>G</td><td>1</td><td>90</td><td>0</td><td>99</td><td>synonymous_variant</td><td></td><td>UNDEF44</td></tr>
<tr><td>gi|696447795|gb|JRIS01000048.1|</td><td>158812</td><td>C</td><td>T</td><td>1</td><td>99</td><td>0</td><td>99</td><td>synonymous_variant</td><td></td><td>UNDEF44</td></tr>
<tr><td>gi|696447795|gb|JRIS01000048.1|</td><td>158813</td><td>T</td><td>G</td><td>1</td><td>99</td><td>0</td><td>99</td><td>missense_variant</td><td></td><td>UNDEF44</td></tr>
<tr><td>gi|696447795|gb|JRIS01000048.1|</td><td>158816</td><td>T</td><td>A</td><td>1</td><td>99</td><td>0</td><td>99</td><td>missense_variant</td><td></td><td>UNDEF44</td></tr>
<tr><td>gi|696447795|gb|JRIS01000048.1|</td><td>158820</td><td>C</td><td>T</td><td>1</td><td>99</td><td>0</td><td>99</td><td>missense_variant</td><td></td><td>UNDEF44</td></tr>
<tr><td>gi|696447795|gb|JRIS01000048.1|</td><td>158821</td><td>G</td><td>A</td><td>1</td><td>99</td><td>0</td><td>99</td><td>synonymous_variant</td><td></td><td>UNDEF44</td></tr>
<tr><td>gi|696447795|gb|JRIS01000048.1|</td><td>158824</td><td>C</td><td>G</td><td>1</td><td>99</td><td>0</td><td>99</td><td>synonymous_variant</td><td></td><td>UNDEF44</td></tr>
<tr><td>gi|696447795|gb|JRIS01000048.1|</td><td>158831</td><td>C</td><td>T</td><td>1</td><td>99</td><td>0</td><td>99</td><td>missense_variant</td><td></td><td>UNDEF44</td></tr>
<tr><td>gi|696447795|gb|JRIS01000048.1|</td><td>158836</td><td>G</td><td>C</td><td>1</td><td>99</td><td>0</td><td>99</td><td>synonymous_variant</td><td></td><td>UNDEF44</td></tr>
<tr><td>gi|696447795|gb|JRIS01000048.1|</td><td>158840</td><td>T</td><td>A</td><td>1</td><td>99</td><td>0</td><td>99</td><td>missense_variant</td><td></td><td>UNDEF44</td></tr>
<tr><td>gi|696447795|gb|JRIS01000048.1|</td><td>158841</td><td>T</td><td>G</td><td>1</td><td>99</td><td>0</td><td>99</td><td>missense_variant</td><td></td><td>UNDEF44</td></tr>
<tr><td>gi|696447795|gb|JRIS01000048.1|</td><td>158843</td><td>A</td><td>G</td><td>1</td><td>99</td><td>0</td><td>99</td><td>missense_variant</td><td></td><td>UNDEF44</td></tr>
<tr><td>gi|696447795|gb|JRIS01000048.1|</td><td>158846</td><td>C</td><td>G</td><td>1</td><td>99</td><td>0</td><td>99</td><td>missense_variant</td><td></td><td>UNDEF44</td></tr>
<tr><td>gi|696447795|gb|JRIS01000048.1|</td><td>158857</td><td>C</td><td>T</td><td>1</td><td>99</td><td>0</td><td>99</td><td>synonymous_variant</td><td></td><td>UNDEF44</td></tr>
<tr><td>gi|696447795|gb|JRIS01000048.1|</td><td>158884</td><td>T</td><td>C</td><td>1</td><td>99</td><td>0</td><td>99</td><td>synonymous_variant</td><td></td><td>UNDEF44</td></tr>
<tr><td>gi|696447795|gb|JRIS01000048.1|</td><td>158893</td><td>G</td><td>A</td><td>1</td><td>99</td><td>0</td><td>99</td><td>synonymous_variant</td><td></td><td>UNDEF44</td></tr>
<tr><td>gi|696447795|gb|JRIS01000048.1|</td><td>158941</td><td>A</td><td>G</td><td>0</td><td>88</td><td>1</td><td>99</td><td>synonymous_variant</td><td></td><td>UNDEF44</td></tr>
<tr><td>gi|696447795|gb|JRIS01000048.1|</td><td>159298</td><td>T</td><td>C</td><td></td><td></td><td>1</td><td>88</td><td>upstream_gene_variant</td><td>DYS1</td><td>YHR068W</td></tr>
<tr><td>gi|696447790|gb|JRIS01000049.1|</td><td>6924</td><td>C</td><td>T</td><td></td><td></td><td>1</td><td>67</td><td>upstream_gene_variant</td><td>CDC53</td><td>YDL132W</td></tr>
<tr><td>gi|696447774|gb|JRIS01000053.1|</td><td>29830</td><td>A</td><td>G</td><td></td><td></td><td>1</td><td>99</td><td>upstream_gene_variant</td><td>PHO87</td><td>YCR037C</td></tr>
<tr><td>gi|696447770|gb|JRIS01000054.1|</td><td>589</td><td>G</td><td>A</td><td></td><td></td><td>1</td><td>86</td><td>missense_variant</td><td></td><td>UNDEF50</td></tr>
<tr><td>gi|696447770|gb|JRIS01000054.1|</td><td>631</td><td>T</td><td>G</td><td></td><td></td><td>1</td><td>80</td><td>missense_variant</td><td></td><td>UNDEF50</td></tr>
<tr><td>gi|696447766|gb|JRIS01000055.1|</td><td>31006</td><td>A</td><td>G</td><td></td><td></td><td>1</td><td>99</td><td>upstream_gene_variant</td><td>GPI2</td><td>YPL076W</td></tr>
<tr><td>gi|696447766|gb|JRIS01000055.1|</td><td>31015</td><td>A</td><td>G</td><td></td><td></td><td>1</td><td>99</td><td>upstream_gene_variant</td><td>GPI2</td><td>YPL076W</td></tr>
<tr><td>gi|696447766|gb|JRIS01000055.1|</td><td>31018</td><td>C</td><td>T</td><td></td><td></td><td>1</td><td>99</td><td>upstream_gene_variant</td><td>GPI2</td><td>YPL076W</td></tr>
<tr><td>gi|696447766|gb|JRIS01000055.1|</td><td>31030</td><td>C</td><td>T</td><td></td><td></td><td>1</td><td>99</td><td>upstream_gene_variant</td><td>GPI2</td><td>YPL076W</td></tr>
<tr><td>gi|696447766|gb|JRIS01000055.1|</td><td>31042</td><td>G</td><td>A</td><td></td><td></td><td>1</td><td>99</td><td>upstream_gene_variant</td><td>GPI2</td><td>YPL076W</td></tr>
<tr><td>gi|696447766|gb|JRIS01000055.1|</td><td>31051</td><td>C</td><td>T</td><td></td><td></td><td>1</td><td>99</td><td>upstream_gene_variant</td><td>GPI2</td><td>YPL076W</td></tr>
<tr><td>gi|696447766|gb|JRIS01000055.1|</td><td>31054</td><td>G</td><td>A</td><td></td><td></td><td>1</td><td>99</td><td>upstream_gene_variant</td><td>GPI2</td><td>YPL076W</td></tr>
<tr><td>gi|696447766|gb|JRIS01000055.1|</td><td>31060</td><td>A</td><td>G</td><td></td><td></td><td>1</td><td>99</td><td>upstream_gene_variant</td><td>GPI2</td><td>YPL076W</td></tr>
<tr><td>gi|696447766|gb|JRIS01000055.1|</td><td>31069</td><td>C</td><td>T</td><td></td><td></td><td>1</td><td>99</td><td>upstream_gene_variant</td><td>GPI2</td><td>YPL076W</td></tr>
<tr><td>gi|696447766|gb|JRIS01000055.1|</td><td>31071</td><td>A</td><td>G</td><td></td><td></td><td>1</td><td>99</td><td>upstream_gene_variant</td><td>GPI2</td><td>YPL076W</td></tr>
<tr><td>gi|696447766|gb|JRIS01000055.1|</td><td>31075</td><td>T</td><td>C</td><td></td><td></td><td>1</td><td>99</td><td>upstream_gene_variant</td><td>GPI2</td><td>YPL076W</td></tr>
<tr><td>gi|696447766|gb|JRIS01000055.1|</td><td>31078</td><td>G</td><td>A</td><td></td><td></td><td>1</td><td>99</td><td>upstream_gene_variant</td><td>GPI2</td><td>YPL076W</td></tr>
<tr><td>gi|696447766|gb|JRIS01000055.1|</td><td>31084</td><td>G</td><td>A</td><td></td><td></td><td>1</td><td>99</td><td>upstream_gene_variant</td><td>GPI2</td><td>YPL076W</td></tr>
<tr><td>gi|696447766|gb|JRIS01000055.1|</td><td>31126</td><td>A</td><td>T</td><td></td><td></td><td>1</td><td>99</td><td>upstream_gene_variant</td><td>GPI2</td><td>YPL076W</td></tr>
<tr><td>gi|696447766|gb|JRIS01000055.1|</td><td>31129</td><td>T</td><td>C</td><td></td><td></td><td>1</td><td>99</td><td>upstream_gene_variant</td><td>GPI2</td><td>YPL076W</td></tr>
<tr><td>gi|696447766|gb|JRIS01000055.1|</td><td>31141</td><td>G</td><td>T</td><td></td><td></td><td>1</td><td>99</td><td>upstream_gene_variant</td><td>GPI2</td><td>YPL076W</td></tr>
<tr><td>gi|696447766|gb|JRIS01000055.1|</td><td>31156</td><td>T</td><td>C</td><td></td><td></td><td>1</td><td>99</td><td>upstream_gene_variant</td><td>GPI2</td><td>YPL076W</td></tr>
<tr><td>gi|696447766|gb|JRIS01000055.1|</td><td>31162</td><td>C</td><td>T</td><td></td><td></td><td>1</td><td>99</td><td>upstream_gene_variant</td><td>GPI2</td><td>YPL076W</td></tr>
<tr><td>gi|696447766|gb|JRIS01000055.1|</td><td>31980</td><td>G</td><td>GA</td><td>1</td><td>99</td><td></td><td></td><td>upstream_gene_variant</td><td>MOT1</td><td>YPL082C</td></tr>
<tr><td>gi|696447735|gb|JRIS01000063.1|</td><td>130139</td><td>C</td><td>CTCTTTTTTTTTTTTTTTTTTTTTTTTTT,CTCTTTTTTTTTTT
TTTTTTTTTTTT</td><td>2</td><td>99</td><td>1</td><td>99</td><td>upstream_gene_variant</td><td>PET9</td><td>YBL030C</td></tr>
<tr><td>gi|696447727|gb|JRIS01000065.1|</td><td>653</td><td>G</td><td>C</td><td>1</td><td>64</td><td></td><td></td><td>downstream_gene_variant</td><td>YIL169C</td><td>YIL169C</td></tr>
<tr><td>gi|696447717|gb|JRIS01000068.1|</td><td>62054</td><td>C</td><td>CAACAAAAAAAAAAAAAAAAAAAAAAAAAAAAAAAAG
A,CAACAAAAAAAAAAAAAAAAAAAAAAAAAAAAAAAG
A</td><td>2</td><td>88</td><td>1</td><td>99</td><td>upstream_gene_variant</td><td>MET28</td><td>YIR017C</td></tr>
<tr><td>gi|696447713|gb|JRIS01000069.1|</td><td>149</td><td>G</td><td>A</td><td></td><td></td><td>1</td><td>99</td><td>upstream_gene_variant</td><td>MRH4</td><td>YGL064C</td></tr>
<tr><td>gi|696447713|gb|JRIS01000069.1|</td><td>246160</td><td>T</td><td>A,TA</td><td>2</td><td>88</td><td>1</td><td>31</td><td>upstream_gene_variant</td><td>COX13</td><td>YGL191W</td></tr>
<tr><td>gi|696447699|gb|JRIS01000073.1|</td><td>885</td><td>G</td><td>A</td><td>1</td><td>99</td><td></td><td></td><td>synonymous_variant</td><td>PAU17</td><td>YLL025W</td></tr>
<tr><td>gi|696447699|gb|JRIS01000073.1|</td><td>921</td><td>A</td><td>G</td><td>1</td><td>99</td><td></td><td></td><td>synonymous_variant</td><td>PAU17</td><td>YLL025W</td></tr>
<tr><td>gi|696447699|gb|JRIS01000073.1|</td><td>924</td><td>T</td><td>C</td><td>1</td><td>99</td><td></td><td></td><td>synonymous_variant</td><td>PAU17</td><td>YLL025W</td></tr>
<tr><td>gi|696447699|gb|JRIS01000073.1|</td><td>939</td><td>A</td><td>G,T</td><td>2</td><td>99</td><td>1</td><td>99</td><td>synonymous_variant</td><td>PAU17</td><td>YLL025W</td></tr>
<tr><td>gi|696447699|gb|JRIS01000073.1|</td><td>945</td><td>G</td><td>A</td><td>1</td><td>99</td><td></td><td></td><td>synonymous_variant</td><td>PAU17</td><td>YLL025W</td></tr>
<tr><td>gi|696447699|gb|JRIS01000073.1|</td><td>950</td><td>T</td><td>A</td><td>1</td><td>99</td><td></td><td></td><td>missense_variant</td><td>PAU17</td><td>YLL025W</td></tr>
<tr><td>gi|696447699|gb|JRIS01000073.1|</td><td>951</td><td>A</td><td>G</td><td>1</td><td>99</td><td></td><td></td><td>synonymous_variant</td><td>PAU17</td><td>YLL025W</td></tr>
<tr><td>gi|696447699|gb|JRIS01000073.1|</td><td>959</td><td>C</td><td>G</td><td>1</td><td>99</td><td></td><td></td><td>missense_variant</td><td>PAU17</td><td>YLL025W</td></tr>
<tr><td>gi|696447699|gb|JRIS01000073.1|</td><td>981</td><td>A</td><td>G</td><td>1</td><td>99</td><td></td><td></td><td>synonymous_variant</td><td>PAU17</td><td>YLL025W</td></tr>
<tr><td>gi|696447699|gb|JRIS01000073.1|</td><td>990</td><td>G</td><td>A</td><td>1</td><td>99</td><td></td><td></td><td>synonymous_variant</td><td>PAU17</td><td>YLL025W</td></tr>
<tr><td>gi|696447699|gb|JRIS01000073.1|</td><td>996</td><td>T</td><td>C</td><td>1</td><td>99</td><td></td><td></td><td>synonymous_variant</td><td>PAU17</td><td>YLL025W</td></tr>
<tr><td>gi|696447699|gb|JRIS01000073.1|</td><td>1002</td><td>A</td><td>G</td><td>1</td><td>99</td><td>0</td><td>49</td><td>synonymous_variant</td><td>PAU17</td><td>YLL025W</td></tr>
<tr><td>gi|696447699|gb|JRIS01000073.1|</td><td>1007</td><td>G</td><td>A</td><td>1</td><td>99</td><td></td><td></td><td>synonymous_variant</td><td>PAU17</td><td>YLL025W</td></tr>
<tr><td>gi|696447695|gb|JRIS01000074.1|</td><td>1052</td><td>G</td><td>A</td><td></td><td></td><td>1</td><td>99</td><td>missense_variant</td><td>YNL019C</td><td>YNL019C</td></tr>
<tr><td>gi|696447695|gb|JRIS01000074.1|</td><td>1172</td><td>C</td><td>T</td><td>0</td><td>99</td><td>1</td><td>99</td><td>missense_variant&splice_region_variant</td><td>YNL019C</td><td>YNL019C</td></tr>
<tr><td>gi|696447695|gb|JRIS01000074.1|</td><td>1371</td><td>T</td><td>C</td><td>0</td><td>99</td><td>1</td><td>99</td><td>upstream_gene_variant</td><td>YNL019C</td><td>YNL019C_circ</td></tr>
<tr><td>gi|696447695|gb|JRIS01000074.1|</td><td>1433</td><td>T</td><td>G</td><td>0</td><td>99</td><td>1</td><td>61</td><td>upstream_gene_variant</td><td>YNL019C</td><td>YNL019C_circ</td></tr>
<tr><td>gi|696447695|gb|JRIS01000074.1|</td><td>1624</td><td>A</td><td>G</td><td>1</td><td>99</td><td></td><td></td><td>upstream_gene_variant</td><td>YNL019C</td><td>YNL019C_circ</td></tr>
<tr><td>gi|696447695|gb|JRIS01000074.1|</td><td>1744</td><td>A</td><td>G</td><td>0</td><td>99</td><td>1</td><td>99</td><td>upstream_gene_variant</td><td>YNL019C</td><td>YNL019C_circ</td></tr>
<tr><td>gi|696447695|gb|JRIS01000074.1|</td><td>1797</td><td>G</td><td>A</td><td>1</td><td>99</td><td>0</td><td>99</td><td>upstream_gene_variant</td><td>YNL019C</td><td>YNL019C_circ</td></tr>
<tr><td>gi|696447695|gb|JRIS01000074.1|</td><td>1811</td><td>T</td><td>C</td><td>1</td><td>99</td><td>0</td><td>27</td><td>upstream_gene_variant</td><td>YNL019C</td><td>YNL019C_circ</td></tr>
<tr><td>gi|696447695|gb|JRIS01000074.1|</td><td>1855</td><td>C</td><td>T</td><td>1</td><td>99</td><td>0</td><td>99</td><td>upstream_gene_variant</td><td>YNL019C</td><td>YNL019C_circ</td></tr>
<tr><td>gi|696447695|gb|JRIS01000074.1|</td><td>1977</td><td>T</td><td>G</td><td>0</td><td>65</td><td>1</td><td>99</td><td>upstream_gene_variant</td><td>YNL019C</td><td>YNL019C_circ</td></tr>
<tr><td>gi|696447695|gb|JRIS01000074.1|</td><td>1978</td><td>A</td><td>G</td><td>0</td><td>8</td><td>1</td><td>99</td><td>upstream_gene_variant</td><td>YNL019C</td><td>YNL019C_circ</td></tr>
<tr><td>gi|696447695|gb|JRIS01000074.1|</td><td>1988</td><td>C</td><td>G</td><td>0</td><td>14</td><td>1</td><td>90</td><td>upstream_gene_variant</td><td>YNL019C</td><td>YNL019C_circ</td></tr>
<tr><td>gi|696447695|gb|JRIS01000074.1|</td><td>2004</td><td>A</td><td>G</td><td>0</td><td>95</td><td>1</td><td>45</td><td>upstream_gene_variant</td><td>YNL019C</td><td>YNL019C_circ</td></tr>
<tr><td>gi|696447695|gb|JRIS01000074.1|</td><td>2066</td><td>C</td><td>T</td><td></td><td></td><td>1</td><td>43</td><td>upstream_gene_variant</td><td>YNL019C</td><td>YNL019C</td></tr>
<tr><td>gi|696447692|gb|JRIS01000075.1|</td><td>64342</td><td>C</td><td>T</td><td>1</td><td>99</td><td>0</td><td>99</td><td>upstream_gene_variant</td><td>ALE1</td><td>YOR175C</td></tr>
<tr><td>gi|696447667|gb|JRIS01000082.1|</td><td>24360</td><td>G</td><td>A</td><td>0</td><td>99</td><td>1</td><td>83</td><td>upstream_gene_variant</td><td>YGR111W</td><td>YGR111W</td></tr>
<tr><td>gi|696447667|gb|JRIS01000082.1|</td><td>24364</td><td>C</td><td>T</td><td>0</td><td>99</td><td>1</td><td>80</td><td>upstream_gene_variant</td><td>YGR111W</td><td>YGR111W</td></tr>
<tr><td>gi|696447667|gb|JRIS01000082.1|</td><td>25927</td><td>G</td><td>A</td><td>0</td><td>99</td><td>1</td><td>47</td><td>upstream_gene_variant</td><td>CLD1</td><td>YGR110W</td></tr>
<tr><td>gi|696447667|gb|JRIS01000082.1|</td><td>26011</td><td>G</td><td>A</td><td>1</td><td>99</td><td>0</td><td>93</td><td>upstream_gene_variant</td><td>CLD1</td><td>YGR110W</td></tr>
<tr><td>gi|696447667|gb|JRIS01000082.1|</td><td>26930</td><td>A</td><td>G</td><td>1</td><td>41</td><td>0</td><td>99</td><td>upstream_gene_variant</td><td>CAB2</td><td>YIL083C</td></tr>
<tr><td>gi|696447667|gb|JRIS01000082.1|</td><td>27524</td><td>C</td><td>T</td><td>0</td><td>99</td><td>1</td><td>99</td><td>synonymous_variant</td><td>YIL082W</td><td>YIL082W</td></tr>
<tr><td>gi|696447659|gb|JRIS01000084.1|</td><td>56773</td><td>T</td><td>G</td><td>0</td><td>99</td><td>1</td><td>99</td><td>missense_variant</td><td>TOR2</td><td>YKL203C</td></tr>
<tr><td>gi|696447656|gb|JRIS01000085.1|</td><td>20480</td><td>T</td><td>C</td><td>1</td><td>53</td><td>0</td><td>65</td><td>missense_variant</td><td>CDC4</td><td>YFL009W</td></tr>
<tr><td>gi|696447656|gb|JRIS01000085.1|</td><td>33894</td><td>ATTTTTTTTTTTTTTTTTTTTTTTTTTTTTTTTTTTTTTT</td><td>A</td><td>0</td><td>99</td><td>1</td><td>99</td><td>upstream_gene_variant</td><td>HSP12</td><td>YFL014W</td></tr>
<tr><td>gi|696447656|gb|JRIS01000085.1|</td><td>33952</td><td>T</td><td>A</td><td></td><td></td><td>1</td><td>99</td><td>upstream_gene_variant</td><td>HSP12</td><td>YFL014W</td></tr>
<tr><td>gi|696447656|gb|JRIS01000085.1|</td><td>33956</td><td>T</td><td>A</td><td>0</td><td>99</td><td>1</td><td>99</td><td>upstream_gene_variant</td><td>HSP12</td><td>YFL014W</td></tr>
<tr><td>gi|696447646|gb|JRIS01000088.1|</td><td>46918</td><td>G</td><td>A</td><td>1</td><td>84</td><td>.</td><td>.</td><td>upstream_gene_variant</td><td>CBF2</td><td>YGR140W</td></tr>
<tr><td>gi|696447632|gb|JRIS01000092.1|</td><td>1785</td><td>C</td><td>T</td><td>1</td><td>88</td><td></td><td></td><td>downstream_gene_variant</td><td>YNR066C</td><td>YNR066C</td></tr>
<tr><td>gi|696447632|gb|JRIS01000092.1|</td><td>1806</td><td>C</td><td>T</td><td>1</td><td>87</td><td></td><td></td><td>downstream_gene_variant</td><td>YNR066C</td><td>YNR066C</td></tr>
<tr><td>gi|696447632|gb|JRIS01000092.1|</td><td>1830</td><td>A</td><td>G</td><td>1</td><td>82</td><td></td><td></td><td>downstream_gene_variant</td><td>YNR066C</td><td>YNR066C</td></tr>
<tr><td>gi|696447632|gb|JRIS01000092.1|</td><td>1848</td><td>A</td><td>G</td><td>1</td><td>84</td><td></td><td></td><td>downstream_gene_variant</td><td>YNR066C</td><td>YNR066C</td></tr>
<tr><td>gi|696447629|gb|JRIS01000093.1|</td><td>63035</td><td>C</td><td>CGGGGGGGGG</td><td></td><td></td><td>1</td><td>90</td><td>upstream_gene_variant</td><td>STE24</td><td>YJR117W</td></tr>
<tr><td>gi|696447626|gb|JRIS01000094.1|</td><td>33656</td><td>A</td><td>AGTAGCTTGAACTTGACCATCACCGATTTGAGAAACG
GCAGCAGCGGTGCTCTTTGGGGCTAAAGTAGTGGTG</td><td>0</td><td>99</td><td>1</td><td>99</td><td>disruptive_inframe_insertion</td><td>HSP150</td><td>YJL159W</td></tr>
<tr><td>gi|696447622|gb|JRIS01000095.1|</td><td>13665</td><td>T</td><td>C</td><td></td><td></td><td>1</td><td>99</td><td>upstream_gene_variant</td><td>YNR073C</td><td>YNR073C</td></tr>
<tr><td>gi|696447622|gb|JRIS01000095.1|</td><td>13685</td><td>A</td><td>C</td><td></td><td></td><td>1</td><td>99</td><td>upstream_gene_variant</td><td>YNR073C</td><td>YNR073C</td></tr>
<tr><td>gi|696447622|gb|JRIS01000095.1|</td><td>13690</td><td>T</td><td>TC</td><td></td><td></td><td>1</td><td>99</td><td>upstream_gene_variant</td><td>YNR073C</td><td>YNR073C</td></tr>
<tr><td>gi|696447622|gb|JRIS01000095.1|</td><td>13695</td><td>C</td><td>T</td><td></td><td></td><td>1</td><td>99</td><td>upstream_gene_variant</td><td>YNR073C</td><td>YNR073C</td></tr>
<tr><td>gi|696447622|gb|JRIS01000095.1|</td><td>13703</td><td>G</td><td>A</td><td></td><td></td><td>1</td><td>99</td><td>upstream_gene_variant</td><td>YNR073C</td><td>YNR073C</td></tr>
<tr><td>gi|696447622|gb|JRIS01000095.1|</td><td>13716</td><td>G</td><td>C</td><td></td><td></td><td>1</td><td>99</td><td>upstream_gene_variant</td><td>YNR073C</td><td>YNR073C</td></tr>
<tr><td>gi|696447622|gb|JRIS01000095.1|</td><td>13726</td><td>T</td><td>C</td><td></td><td></td><td>1</td><td>99</td><td>upstream_gene_variant</td><td>YNR073C</td><td>YNR073C</td></tr>
<tr><td>gi|696447622|gb|JRIS01000095.1|</td><td>13745</td><td>C</td><td>T</td><td></td><td></td><td>1</td><td>99</td><td>upstream_gene_variant</td><td>YNR073C</td><td>YNR073C</td></tr>
<tr><td>gi|696447622|gb|JRIS01000095.1|</td><td>13751</td><td>C</td><td>T</td><td></td><td></td><td>1</td><td>99</td><td>upstream_gene_variant</td><td>YNR073C</td><td>YNR073C</td></tr>
<tr><td>gi|696447622|gb|JRIS01000095.1|</td><td>13766</td><td>G</td><td>C</td><td></td><td></td><td>1</td><td>99</td><td>upstream_gene_variant</td><td>YNR073C</td><td>YNR073C</td></tr>
<tr><td>gi|696447622|gb|JRIS01000095.1|</td><td>13769</td><td>T</td><td>G</td><td></td><td></td><td>1</td><td>99</td><td>upstream_gene_variant</td><td>YNR073C</td><td>YNR073C</td></tr>
<tr><td>gi|696447622|gb|JRIS01000095.1|</td><td>13771</td><td>A</td><td>T</td><td></td><td></td><td>1</td><td>99</td><td>upstream_gene_variant</td><td>YNR073C</td><td>YNR073C</td></tr>
<tr><td>gi|696447622|gb|JRIS01000095.1|</td><td>13774</td><td>C</td><td>T</td><td></td><td></td><td>1</td><td>99</td><td>upstream_gene_variant</td><td>YNR073C</td><td>YNR073C</td></tr>
<tr><td>gi|696447622|gb|JRIS01000095.1|</td><td>13788</td><td>C</td><td>T</td><td></td><td></td><td>1</td><td>99</td><td>upstream_gene_variant</td><td>YNR073C</td><td>YNR073C</td></tr>
<tr><td>gi|696447622|gb|JRIS01000095.1|</td><td>13800</td><td>G</td><td>A</td><td></td><td></td><td>1</td><td>99</td><td>upstream_gene_variant</td><td>YNR073C</td><td>YNR073C</td></tr>
<tr><td>gi|696447622|gb|JRIS01000095.1|</td><td>13807</td><td>C</td><td>G</td><td></td><td></td><td>1</td><td>99</td><td>upstream_gene_variant</td><td>YNR073C</td><td>YNR073C</td></tr>
<tr><td>gi|696447622|gb|JRIS01000095.1|</td><td>13808</td><td>T</td><td>G</td><td></td><td></td><td>1</td><td>99</td><td>upstream_gene_variant</td><td>YNR073C</td><td>YNR073C</td></tr>
<tr><td>gi|696447622|gb|JRIS01000095.1|</td><td>13833</td><td>C</td><td>CAA</td><td></td><td></td><td>1</td><td>99</td><td>upstream_gene_variant</td><td>YNR073C</td><td>YNR073C</td></tr>
<tr><td>gi|696447622|gb|JRIS01000095.1|</td><td>14073</td><td>A</td><td>G</td><td></td><td></td><td>1</td><td>99</td><td>synonymous_variant</td><td>YNR073C</td><td>YNR073C</td></tr>
<tr><td>gi|696447622|gb|JRIS01000095.1|</td><td>14172</td><td>T</td><td>C</td><td></td><td></td><td>1</td><td>99</td><td>synonymous_variant</td><td>YNR073C</td><td>YNR073C</td></tr>
<tr><td>gi|696447609|gb|JRIS01000099.1|</td><td>1810</td><td>A</td><td>G</td><td>0</td><td>99</td><td>1</td><td>97</td><td>upstream_gene_variant</td><td>TDA8</td><td>YAL064C-A</td></tr>
<tr><td>gi|696447605|gb|JRIS01000100.1|</td><td>33431</td><td>T</td><td>C</td><td>0</td><td>99</td><td>1</td><td>99</td><td>upstream_gene_variant</td><td>EFT1</td><td>YOR133W</td></tr>
<tr><td>gi|696447592|gb|JRIS01000103.1|</td><td>68377</td><td>T</td><td>A</td><td>0</td><td>99</td><td>1</td><td>99</td><td>upstream_gene_variant</td><td>RTT105</td><td>YER104W</td></tr>
<tr><td>gi|696447592|gb|JRIS01000103.1|</td><td>107515</td><td>C</td><td>T</td><td></td><td></td><td>1</td><td>99</td><td>synonymous_variant</td><td>SER3</td><td>YER081W</td></tr>
<tr><td>gi|696447592|gb|JRIS01000103.1|</td><td>107518</td><td>G</td><td>C</td><td></td><td></td><td>1</td><td>99</td><td>synonymous_variant</td><td>SER3</td><td>YER081W</td></tr>
<tr><td>gi|696447592|gb|JRIS01000103.1|</td><td>107532</td><td>G</td><td>A</td><td></td><td></td><td>1</td><td>99</td><td>synonymous_variant</td><td>SER3</td><td>YER081W</td></tr>
<tr><td>gi|696447592|gb|JRIS01000103.1|</td><td>107563</td><td>G</td><td>A</td><td></td><td></td><td>1</td><td>99</td><td>synonymous_variant</td><td>SER3</td><td>YER081W</td></tr>
<tr><td>gi|696447592|gb|JRIS01000103.1|</td><td>107581</td><td>C</td><td>G</td><td></td><td></td><td>1</td><td>99</td><td>synonymous_variant</td><td>SER3</td><td>YER081W</td></tr>
<tr><td>gi|696447592|gb|JRIS01000103.1|</td><td>107584</td><td>G</td><td>A</td><td></td><td></td><td>1</td><td>99</td><td>synonymous_variant</td><td>SER3</td><td>YER081W</td></tr>
<tr><td>gi|696447592|gb|JRIS01000103.1|</td><td>107599</td><td>G</td><td>A</td><td></td><td></td><td>1</td><td>99</td><td>synonymous_variant</td><td>SER3</td><td>YER081W</td></tr>
<tr><td>gi|696447592|gb|JRIS01000103.1|</td><td>107608</td><td>A</td><td>G</td><td></td><td></td><td>1</td><td>99</td><td>synonymous_variant</td><td>SER3</td><td>YER081W</td></tr>
<tr><td>gi|696447592|gb|JRIS01000103.1|</td><td>168974</td><td>A</td><td>ATTTTTTTTTTTTTTTTTTT,ATTTTTTTTTTTTTTTTTTTT</td><td>1</td><td>81</td><td>2</td><td>99</td><td>upstream_gene_variant</td><td>PIC2</td><td>YER053C</td></tr>
<tr><td>gi|696447592|gb|JRIS01000103.1|</td><td>224570</td><td>C</td><td>T</td><td>1</td><td>99</td><td>0</td><td>99</td><td>missense_variant</td><td>GCD11</td><td>YER025W</td></tr>
</table>

gi|696447583|gb|JRIS01000105.1|	14205	T	C	1	99			upstream_gene_variant	NTC20	YBR188C
gi|696447583|gb|JRIS01000105.1|	14209	A	C	1	99			upstream_gene_variant	NTC20	YBR188C
gi|696447583|gb|JRIS01000105.1|	14211	G	T	1	99			upstream_gene_variant	NTC20	YBR188C
gi|696447583|gb|JRIS01000105.1|	14214	C	T	1	99			upstream_gene_variant	NTC20	YBR188C
gi|696447583|gb|JRIS01000105.1|	14217	C	T	1	99			upstream_gene_variant	NTC20	YBR188C
gi|696447583|gb|JRIS01000105.1|	14227	A	G	1	99			upstream_gene_variant	NTC20	YBR188C
gi|696447574|gb|JRIS01000107.1|	47417	TA	T	1	99	0	99	upstream_gene_variant	YSH1	YLR277C
gi|696447565|gb|JRIS01000110.1|	160312	T	C	0	99	1	59	upstream_gene_variant	YNL115C	YNL115C
gi|696447565|gb|JRIS01000110.1|	161918	T	TC	0	99	1	86	upstream_gene_variant	YNL115C	YNL115C
gi|696447565|gb|JRIS01000110.1|	161945	C	T			1	45	upstream_gene_variant	YNL115C	YNL115C
gi|696447550|gb|JRIS01000114.1|	1163	T	TTATATAAA	1	99			upstream_gene_variant	CDS1	YBR029C
gi|696447536|gb|JRIS01000118.1|	15018	C	A	1	99	0	99	missense_variant	DYN1	YKR054C
gi|696447536|gb|JRIS01000118.1|	32097	GA	G	1	80	0	99	upstream_gene_variant	TRK2	YKR050W
gi|696447526|gb|JRIS01000121.1|	622	T	A	1	99			synonymous_variant	YOL166C	YOL166C
gi|696447478|gb|JRIS01000134.1|	42257	G	GAAAAAAAAAAAAAAAAACACAGAAAAGAATGCAGAAAGATGTCAACTGAAAAAAAAAAAGGTGAACACAGGAAAAAAAAT			1	99	upstream_gene_variant	SSC1	YJR045C
gi|696447464|gb|JRIS01000138.1|	16156	T	TCGAAAAAAAAAAAAAAAAAAAAAAAAGAAAAGAAGC,TCGAAAAAAAAAAAAAAAAAAAAAAAAAAAGAAAAGAAGC	2	99	1	98	upstream_gene_variant	YOR338W	YOR338W
gi|696447464|gb|JRIS01000138.1|	25814	A	AGAAG	1	99	0	99	upstream_gene_variant	MRS2	YOR334W
gi|696447464|gb|JRIS01000138.1|	25825	A	AGAGAC	1	99	0	99	upstream_gene_variant	MRS2	YOR334W
gi|696447456|gb|JRIS01000140.1|	23490	A	T			1	99	downstream_gene_variant	ADD37	YMR184W
gi|696447456|gb|JRIS01000140.1|	23493	G	A			1	99	downstream_gene_variant	ADD37	YMR184W
gi|696447456|gb|JRIS01000140.1|	23499	T	C			1	99	downstream_gene_variant	ADD37	YMR184W
gi|696447456|gb|JRIS01000140.1|	23517	G	A			1	99	downstream_gene_variant	ADD37	YMR184W
gi|696447456|gb|JRIS01000140.1|	23523	G	T			1	99	downstream_gene_variant	ADD37	YMR184W
gi|696447456|gb|JRIS01000140.1|	23526	C	T			1	99	downstream_gene_variant	ADD37	YMR184W
gi|696447456|gb|JRIS01000140.1|	23532	A	T			1	99	downstream_gene_variant	ADD37	YMR184W
gi|696447456|gb|JRIS01000140.1|	23539	T	C			1	99	downstream_gene_variant	ADD37	YMR184W
gi|696447456|gb|JRIS01000140.1|	23540	TAG	T	0	99	1	99	downstream_gene_variant	ADD37	YMR184W
gi|696447456|gb|JRIS01000140.1|	23544	C	CTA			1	99	downstream_gene_variant	ADD37	YMR184W
gi|696447456|gb|JRIS01000140.1|	23559	T	C			1	99	downstream_gene_variant	ADD37	YMR184W
gi|696447456|gb|JRIS01000140.1|	23850	C	T			1	51	downstream_gene_variant	ADD37	YMR184W
gi|696447451|gb|JRIS01000142.1|	39391	GA	G	0	99	1	60	upstream_gene_variant	MRS6	YOR370C
gi|696447449|gb|JRIS01000143.1|	20050	A	T	1	99			upstream_gene_variant	ARP8	YOR141C
gi|696447444|gb|JRIS01000145.1|	308	T	C			1	99	synonymous_variant	YCR102C	YCR102C
gi|696447444|gb|JRIS01000145.1|	318	A	G			1	99	missense_variant	YCR102C	YCR102C
gi|696447444|gb|JRIS01000145.1|	12282	AAT	A	0	99	1	99	upstream_gene_variant	YCR097W	YCR097W
gi|696447433|gb|JRIS01000148.1|	92199	A	AT	1	99			upstream_gene_variant	SHE3	YBR130C
gi|696447433|gb|JRIS01000148.1|	113252	A	G			1	99	downstream_gene_variant	GRS1	YBR121C
gi|696447429|gb|JRIS01000149.1|	47506	A	ATAACT	0	99	1	99	upstream_gene_variant	RTN2	YDL204W
gi|696447429|gb|JRIS01000149.1|	47510	A	AGAT	0	99	1	99	upstream_gene_variant	RTN2	YDL204W
gi|696447429|gb|JRIS01000149.1|	47513	A	AGGAGATAAGG	0	99	1	99	upstream_gene_variant	RTN2	YDL204W
gi|696447394|gb|JRIS01000158.1|	133106	GA	G	1	55	0	99	upstream_gene_variant	MSA1	YOR066W
gi|696447394|gb|JRIS01000158.1|	261800	C	CTT	1	99			upstream_gene_variant	SIN3	YOL004W
gi|696447367|gb|JRIS01000165.1|	96765	G	GA	1	99	0	99	upstream_gene_variant	VMS1	YDR049W
gi|696447367|gb|JRIS01000165.1|	100581	A	T	0	99	1	99	upstream_gene_variant	HEM13	YDR044W
gi|696447353|gb|JRIS01000169.1|	9116	GA	G	1	99			upstream_gene_variant	ERG11	YHR007C
gi|696447326|gb|JRIS01000175.1|	75535	T	A			1	90	missense_variant	ENO1	YGR254W
gi|696447326|gb|JRIS01000175.1|	75536	G	A			1	90	synonymous_variant	ENO1	YGR254W
gi|696447317|gb|JRIS01000178.1|	53039	A	G			1	99	synonymous_variant	RPL4A	YBR031W
gi|696447304|gb|JRIS01000182.1|	123	G	A			1	99	synonymous_variant	SSB1	YDL229W
gi|696447304|gb|JRIS01000182.1|	234	G	A	1	81	0	99	synonymous_variant	SSB1	YDL229W
gi|696447304|gb|JRIS01000182.1|	249	G	A	1	57	0	99	synonymous_variant	SSB1	YDL229W
gi|696447304|gb|JRIS01000182.1|	312	A	G	1	99	0	99	synonymous_variant	SSB1	YDL229W
gi|696447304|gb|JRIS01000182.1|	321	A	G	1	99	0	99	synonymous_variant	SSB1	YDL229W
gi|696447304|gb|JRIS01000182.1|	489	G	A	0	41	1	99	synonymous_variant	SSB1	YDL229W
gi|696447304|gb|JRIS01000182.1|	537	AG	A	1	99	0	99	frameshift_variant	SSB1	YDL229W
gi|696447304|gb|JRIS01000182.1|	539	C	CTG	1	99	0	99	frameshift_variant	SSB1	YDL229W
gi|696447304|gb|JRIS01000182.1|	542	AT	A	1	99	0	99	frameshift_variant	SSB1	YDL229W
gi|696447304|gb|JRIS01000182.1|	546	A	T	1	99	0	99	synonymous_variant	SSB1	YDL229W
gi|696447304|gb|JRIS01000182.1|	549	A	G	1	99	0	99	synonymous_variant	SSB1	YDL229W
gi|696447304|gb|JRIS01000182.1|	579	A	T	1	99	0	99	synonymous_variant	SSB1	YDL229W
gi|696447304|gb|JRIS01000182.1|	591	A	G	1	90	0	99	synonymous_variant	SSB1	YDL229W
gi|696447304|gb|JRIS01000182.1|	693	G	A	1	99	0	99	synonymous_variant	SSB1	YDL229W
gi|696447300|gb|JRIS01000183.1|	5253	A	G	1	99	0	99	missense_variant	PPX1	YHR201C
gi|696447292|gb|JRIS01000185.1|	3263	A	ATC			1	99	upstream_gene_variant	YPS5	YGL259W
gi|696447292|gb|JRIS01000185.1|	3281	G	A			1	99	upstream_gene_variant	YPS5	YGL259W
gi|696447292|gb|JRIS01000185.1|	3292	C	T			1	99	upstream_gene_variant	YPS5	YGL259W
gi|696447289|gb|JRIS01000186.1|	7587	C	T			1	99	synonymous_variant	ENA5	YDR038C
gi|696447289|gb|JRIS01000186.1|	7613	G	T			1	99	missense_variant	ENA5	YDR038C
gi|696447282|gb|JRIS01000188.1|	762	C	T			1	99	upstream_gene_variant	MDM36	YPR083W
gi|696447274|gb|JRIS01000190.1|	519	C	T			1	99	synonymous_variant	LYS20	YDL182W
gi|696447274|gb|JRIS01000190.1|	549	T	C			1	99	synonymous_variant	LYS20	YDL182W
gi|696447274|gb|JRIS01000190.1|	555	C	T			1	99	synonymous_variant	LYS20	YDL182W
gi|696447274|gb|JRIS01000190.1|	86616	A	G	1	99			upstream_gene_variant	RDI1	YDL135C
gi|696447274|gb|JRIS01000190.1|	86628	G	A	1	99			upstream_gene_variant	RDI1	YDL135C
gi|696447270|gb|JRIS01000191.1|	103480	C	CT	1	84			upstream_gene_variant	RRP46	YGR095C
gi|696447265|gb|JRIS01000192.1|	137194	GA	G	1	99	0	99	upstream_gene_variant	BLI1	YKL061W
gi|696447257|gb|JRIS01000194.1|	24773	C	T			1	91	missense_variant	YNR065C	YNR065C
gi|696447257|gb|JRIS01000194.1|	24778	G	C			1	55	synonymous_variant	YNR065C	YNR065C
gi|696447246|gb|JRIS01000197.1|	3999	G	GA	1	56	0	99	upstream_gene_variant	SPC2	YML055W
gi|696447246|gb|JRIS01000197.1|	9995	C	CT			1	99	upstream_gene_variant	CMP2	YML057W
gi|696447242|gb|JRIS01000198.1|	42814	T	C	1	99	0	99	upstream_gene_variant	COX15	YER141W
gi|696447197|gb|JRIS01000210.1|	96	T	C	0	99	1	99	upstream_gene_variant	YHL034C	YHL034C
gi|696447197|gb|JRIS01000210.1|	103	T	G	0	99	1	99	upstream_gene_variant	YHL034C	YHL034C
gi|696447193|gb|JRIS01000211.1|	315	G	A			1	90	upstream_gene_variant	YDL183C	YDL183C
gi|696447193|gb|JRIS01000211.1|	325	A	G			1	90	upstream_gene_variant	YDL183C	YDL183C
gi|696447193|gb|JRIS01000211.1|	328	T	C			1	90	upstream_gene_variant	YDL183C	YDL183C
gi|696447193|gb|JRIS01000211.1|	329	C	T			1	90	upstream_gene_variant	YDL183C	YDL183C
gi|696447193|gb|JRIS01000211.1|	478	G	A			1	45	upstream_gene_variant	YDL183C	YDL183C
gi|696447193|gb|JRIS01000211.1|	484	A	G			1	45	upstream_gene_variant	YDL183C	YDL183C
gi|696447193|gb|JRIS01000211.1|	514	G	A			1	52	upstream_gene_variant	YDL183C	YDL183C
gi|696447174|gb|JRIS01000216.1|	68214	G	T			1	90	upstream_gene_variant	ADH4	YGL256W
gi|696447174|gb|JRIS01000216.1|	68217	T	C			1	90	upstream_gene_variant	ADH4	YGL256W
gi|696447169|gb|JRIS01000217.1|	81	TCCACACCCACACCCACACA	T	1	99	.	.	intergenic_region		
gi|696447165|gb|JRIS01000218.1|	38619	A	AACCGATGAATTCGAGCTCGTTTTCGACACTGGATGGCGGCGTTAGTATCGAATCGACAGCAGTATAGCGACCAGCATTCACATACGATTGACGCATGATATTACTTTCTGCGCACTTAACTTCGCATCTGGGCAGATGATGTCGAG	0	45	1	99	frameshift_variant&stop_gained	CLN3	YAL040C
gi|696447150|gb|JRIS01000222.1|	49905	C	T	1	45	0	13	upstream_gene_variant	ECM29	YHL030W
gi|696447132|gb|JRIS01000227.1|	96460	G	GA			1	99	upstream_gene_variant	TCP1	YDR212W
gi|696447076|gb|JRIS01000242.1|	416	C	G	0	99	1	99	synonymous_variant	YHR214W	YHR214W
gi|696447076|gb|JRIS01000242.1|	1034	G	A	1	99			upstream_gene_variant	YHR214W	YHR214W
gi|696447076|gb|JRIS01000242.1|	1340	A	T	1	99			upstream_gene_variant	YHR214W	YHR214W
gi|696446946|gb|JRIS01000279.1|	2083	G	T	1	99			stop_gained	YJR157W	YJR157W
gi|696446946|gb|JRIS01000279.1|	2145	T	A	1	99	0	99	stop_gained	YJR157W	YJR157W
gi|696446866|gb|JRIS01000302.1|	1153	T	TA			1	48	downstream_gene_variant	HSP32	YPL280W
gi|696446851|gb|JRIS01000306.1|	123	C	T	1	99	0	99	upstream_gene_variant	GEX1	YCL073C
gi|696446851|gb|JRIS01000306.1|	249	A	C	0	99	1	99	upstream_gene_variant	GEX1	YCL073C
gi|696446851|gb|JRIS01000306.1|	1313	A	C			1	99	synonymous_variant	GEX1	YCL073C
gi|696446851|gb|JRIS01000306.1|	1469	T	C			1	99	synonymous_variant	GEX1	YCL073C
gi|696446851|gb|JRIS01000306.1|	1548	G	A	0	99	1	99	missense_variant	GEX1	YCL073C
gi|696446851|gb|JRIS01000306.1|	1769	A	G	0	99	1	99	synonymous_variant	GEX1	YCL073C
gi|696446851|gb|JRIS01000306.1|	2216	T	C	0	99	1	99	synonymous_variant	GEX1	YCL073C
gi|696446851|gb|JRIS01000306.1|	2297	C	T	0	99	1	99	synonymous_variant	GEX1	YCL073C
gi|696446851|gb|JRIS01000306.1|	2468	G	C	1	99	0	99	synonymous_variant	GEX1	YCL073C
gi|696446851|gb|JRIS01000306.1|	2704	T	C	1	99	0	22	missense_variant	GEX1	YCL073C
gi|696446851|gb|JRIS01000306.1|	2738	C	CA	1	99	0	99	downstream_gene_variant	GEX1	YCL073C
gi|696446844|gb|JRIS01000308.1|	641	G	T	0	99	1	99	upstream_gene_variant	DDI3	YNL335W
gi|696446844|gb|JRIS01000308.1|	674	G	A	0	99	1	99	upstream_gene_variant	DDI3	YNL335W
gi|696446844|gb|JRIS01000308.1|	799	T	G	0	95	1	91	upstream_gene_variant	DDI3	YNL335W
gi|696446844|gb|JRIS01000308.1|	918	T	C			1	99	upstream_gene_variant	DDI3	YNL335W
gi|696446844|gb|JRIS01000308.1|	1218	T	C	0	99	1	99	upstream_gene_variant	DDI3	YNL335W
gi|696446844|gb|JRIS01000308.1|	1615	T	C	1	99	0	99	synonymous_variant	DDI3	YNL335W
gi|696446844|gb|JRIS01000308.1|	2349	T	G	0	35	1	99	missense_variant	SNO2	YNL334C
gi|696446844|gb|JRIS01000308.1|	2699	A	G	1	99	0	99	upstream_gene_variant	SNO2	YNL334C
gi|696446844|gb|JRIS01000308.1|	2903	T	G	0	99	1	99	upstream_gene_variant	SNO2	YNL334C
gi|696446844|gb|JRIS01000308.1|	3006	G	A	1	99	0	99	upstream_gene_variant	SNO2	YNL334C
gi|696446844|gb|JRIS01000308.1|	3831	T	C	1	99			synonymous_variant	SNZ2	YNL333W
gi|696446844|gb|JRIS01000308.1|	3843	G	A	1	99			synonymous_variant	SNZ2	YNL333W
gi|696446844|gb|JRIS01000308.1|	3850	A	G	1	99			missense_variant	SNZ2	YNL333W
gi|696446798|gb|JRIS01000321.1|	1578	T	G	1	99	0	99	missense_variant	PHO12	YHR215W
gi|696446798|gb|JRIS01000321.1|	2365	T	C	1	99	0	99	synonymous_variant	PHO12	YHR215W
gi|696446798|gb|JRIS01000321.1|	3266	A	T	0	99	1	99	upstream_gene_variant	PHO12	YHR215W
gi|696446794|gb|JRIS01000322.1|	403	A	G	1	99	0	99	synonymous_variant	YOR390W	YOR390W
gi|696446794|gb|JRIS01000322.1|	1589	G	T	0	99	1	99	upstream_gene_variant	YOR390W	YOR390W
gi|696446794|gb|JRIS01000322.1|	1599	CT	C	0	99	1	99	upstream_gene_variant	YOR390W	YOR390W
gi|696446794|gb|JRIS01000322.1|	1644	T	G	1	99			upstream_gene_variant	YOR390W	YOR390W
gi|696446794|gb|JRIS01000322.1|	1680	T	A	1	79			upstream_gene_variant	YOR390W	YOR390W
gi|696446729|gb|JRIS01000338.1|	477	C	T			1	99	missense_variant	YBL109W	YBL109W
gi|696446729|gb|JRIS01000338.1|	494	CA	C			1	45	frameshift_variant	YBL109W	YBL109W
gi|696446729|gb|JRIS01000338.1|	548	T	C			1	99	missense_variant	YBL109W	YBL109W
gi|696446729|gb|JRIS01000338.1|	561	G	A			1	99	missense_variant	YBL109W	YBL109W
gi|696446729|gb|JRIS01000338.1|	569	T	C			1	99	missense_variant	YBL109W	YBL109W
gi|696446726|gb|JRIS01000339.1|	510	G	A	1	99			synonymous_variant	YMR326C	YMR326C
gi|696446726|gb|JRIS01000339.1|	519	A	C	1	99			missense_variant	YMR326C	YMR326C
gi|696446726|gb|JRIS01000339.1|	547	A	T			1	99	missense_variant	YMR326C	YMR326C
gi|696446726|gb|JRIS01000339.1|	582	T	TTGAAGTATGAGCACTACCATAACGTCATTAACGTAAAAGTTCCTTAATATTCCCATTTGCTTGAACGGATGCCACTTCAGAATATTTCGTACTTACACAGACTTCACATTAGAATATTATGTCATCCCATTGTTGTGACACTCTTTATTCACCGAGCAATAATACGGTAGTGGCTCACACACA	0	99	1	90	stop_gained&disruptive_inframe_insertion	YMR326C	YMR326C
gi|696446714|gb|JRIS01000342.1|	305	A	G	0	40	1	44	upstream_gene_variant	MAL12	YGR292W
gi|696446714|gb|JRIS01000342.1|	329	G	T	0	99	1	44	upstream_gene_variant	MAL12	YGR292W
gi|696446714|gb|JRIS01000342.1|	401	T	C	0	99	1	99	upstream_gene_variant	MAL12	YGR292W
gi|696446714|gb|JRIS01000342.1|	406	C	T	0	99	1	99	upstream_gene_variant	MAL12	YGR292W
gi|696446714|gb|JRIS01000342.1|	464	A	T	1	99	0	67	upstream_gene_variant	MAL12	YGR292W
gi|696446714|gb|JRIS01000342.1|	720	T	C	1	83	0	99	synonymous_variant	MAL12	YGR292W
gi|696446714|gb|JRIS01000342.1|	2238	G	A	1	99	0	99	downstream_gene_variant	MAL12	YGR292W
gi|696446714|gb|JRIS01000342.1|	2272	C	G	1	99	0	99	downstream_gene_variant	MAL12	YGR292W
gi|696446714|gb|JRIS01000342.1|	2327	G	A	1	99	0	99	downstream_gene_variant	MAL12	YGR292W
gi|696446714|gb|JRIS01000342.1|	2388	C	T	1	99	0	99	downstream_gene_variant	MAL12	YGR292W
gi|696446714|gb|JRIS01000342.1|	2446	A	G	0	99	1	99	downstream_gene_variant	MAL12	YGR292W
gi|696446714|gb|JRIS01000342.1|	2623	T	C	1	99	0	99	downstream_gene_variant	MAL12	YGR292W
gi|696446714|gb|JRIS01000342.1|	2626	G	A	1	99	0	99	downstream_gene_variant	MAL12	YGR292W
gi|696446714|gb|JRIS01000342.1|	2711	G	A	0	99	1	99	downstream_gene_variant	MAL12	YGR292W
gi|696446714|gb|JRIS01000342.1|	2735	T	C	0	99	1	99	downstream_gene_variant	MAL12	YGR292W
gi|696446714|gb|JRIS01000342.1|	2878	C	T	1	99	0	52	downstream_gene_variant	MAL12	YGR292W
gi|696446714|gb|JRIS01000342.1|	2898	G	T	1	99			downstream_gene_variant	MAL12	YGR292W
gi|696446714|gb|JRIS01000342.1|	2928	A	T	1	99	0	99	downstream_gene_variant	MAL12	YGR292W
gi|696446714|gb|JRIS01000342.1|	2941	G	A	1	99	0	99	downstream_gene_variant	MAL12	YGR292W
gi|696446714|gb|JRIS01000342.1|	2949	T	C	1	99	0	99	downstream_gene_variant	MAL12	YGR292W
gi|696446714|gb|JRIS01000342.1|	2977	A	C	1	99	0	99	downstream_gene_variant	MAL12	YGR292W
gi|696446714|gb|JRIS01000342.1|	2988	C	T	1	99	0	99	downstream_gene_variant	MAL12	YGR292W
gi|696446714|gb|JRIS01000342.1|	2999	G	T	1	99	0	99	downstream_gene_variant	MAL12	YGR292W
gi|696446714|gb|JRIS01000342.1|	3000	A	G	1	99	0	99	downstream_gene_variant	MAL12	YGR292W
gi|696446714|gb|JRIS01000342.1|	3016	C	G	1	99	0	99	downstream_gene_variant	MAL12	YGR292W
gi|696446714|gb|JRIS01000342.1|	3033	A	G	1	99	0	99	downstream_gene_variant	MAL12	YGR292W
gi|696446714|gb|JRIS01000342.1|	3043	G	T	1	99	0	99	downstream_gene_variant	MAL12	YGR292W
gi|696446714|gb|JRIS01000342.1|	3056	C	T	1	99	0	99	downstream_gene_variant	MAL12	YGR292W
gi|696446714|gb|JRIS01000342.1|	3062	G	A	1	99	0	99	downstream_gene_variant	MAL12	YGR292W

Supplementary Table 9: Samtools variant analysis of contigs against the S288C reference genome. Columns 1 and 2 detail the position of the variant. Columns 3 and 4 note the reference allele (Genotype 0) and the alternative allele (Genotype 1). Columns 5 and 6 denote the called genotype for strains 1691 and 1693 respectively. Columns 7-9 report the location of the variant and its effect. For the sake of brevity, those variants which are shared between 1691 and 1693 are not shown, and for cases where there were five or more discordant variants called for a single gene, those variants are not listed individually (this was the case for many genes such as FLO9 that appear to contain a structural variation). Those variants that were also called when analyzing the trimmed reads (as opposed to the assembled contigs) are shaded. Clear discordand SNPs that could be confirmed by aligning the reads to IGV are shaded dark grey. Each of these was also called when analyzing trimmed reads (Supplemental Tables 5 & 6).

CHROM	POS	Genotype 0	Genotype 1	1691	1693	Annotation	Gene	Gene ID
I	12690	AGAAGTAATTTCCTGACTTGTTGTTGCACTGGTAA<br>CAGGTGGTAATGATGAAGTAATTTCCTGACTTGT<br>TGTTGTACTGGTAACAGGTGGTAATGATGAAG	AGAAGTAATTTCCTGACTTGTTGTTGCACTGGTAACAGGTGGTAATGATGAAGTAATTTCCTGACTTGTTGTTGTACTGGTAACAGGTGGTAATGATGAAGTAATTTC<br>CTGACTTGTTGTTGTACTGGTAACAGGTGGTAATGAAGAAGTAATTTCCTGACTTGTTGTTGCACTGGTAACAGGTGGTAATGATGAAGTAATTTCCTGACTTGTTGT<br>TGTACTGGTAACAGGTGGTAATGAAGAAGTAATTTCCTGACTTGTTGTTGTACTGGTAACAGGTGGTAATGATGAAGTAATTTCCTGACTTGCTGTCGCACTGGTAA<br>CAGGTGGTAATGAAGAAGTAATTTCCTGACTTGTTGTTGCACTGGTAACAGGTGGTAATGATGAAGTAATTTCCTGACTTGTTGTTGCACTGGTAACAGGTGGTAAT<br>GATGAAGTAATTTCCTGACTTGTTGTTGTACTGGTAACAGGTGGTAATGATGAAG	0	1	upstream_gene_variant	SEO1	YAL067C
I			53 SNPs between 26221 and 26799	-	-	missense_variant	FLO9	YAL063C
I	141030	T	A	1	0	missense_variant	YAL004W	YAL004W
I			59 SNPs between 203639 and 212211	-	-	synonymous_variant	FLO1	YAR050W
I	213637	A	T	1	0	upstream_gene_variant	YAR061W	YAR061W
I	214057	C	A	1	0	upstream_gene_variant	YAR061W	YAR061W
I	214061	C	A	1	0	upstream_gene_variant	YAR061W	YAR061W
I	219252	T	C	1	0	upstream_gene_variant	YAR060C	YAR060C
I	220292	G	C	1	0	missense_variant	YAR064W	YAR064W
I	220382	C	A	1	0	missense_variant	YAR064W	YAR064W
I	220499	T	A	1	0	upstream_gene_variant	YAR060C	YAR060C
I	220805	C	T	1	0	upstream_gene_variant	YAR060C	YAR060C
I	222254	T	C	1	0	upstream_gene_variant	YAR060C	YAR060C
II	68282	GTTATTATTATTATTATTATTATTATTATTATTATT<br>ATTATTATTATTATTATTATTATTATTATT	GTTATTATTATTATTATTATTATTATTATTATTATTATTATTATTATTATTATTATTATTATTATTATTATTATTATTATTATTATTATTATTATT	0	1	conservative_inframe_insertion	CDC27	YBL084C
II	221505	C	T	0	1	missense_variant	YBL005W-A	YBL005W-A
II	224777	G	A	1	0	synonymous_variant	YBL005W-B	YBL005W-B
II	466955	A	C	1	0	upstream_gene_variant	CYC8	YBR112C
III			7 SNPs between 206 and 586	-	-	upstream_gene_variant	YCL076W	YCL076W
III	200119	G	T	1	0	synonymous_variant	MATALPHA2	YCR039C
III	200145	G	T	1	0	missense_variant	MATALPHA2	YCR039C
III	200158	T	C	1	0	synonymous_variant	MATALPHA2	YCR039C
III	200166	G	A	1	0	missense_variant	MATALPHA2	YCR039C
III			36 SNPs between 200188 and 200434	-	-	upstream_gene_variant	PHO87	YCR037C
III			42 SNPs between 200444 and 200850	-	-	start_lost	MATALPHA1	YCR040W
III			55 SNPs between 268850 and 759248	-	-	missense_variant	FIG2	YCR089W
IV	827861	G	T	0	1	missense_variant	CDC1	YDR182W
IV	892698	GTTTTTTTTTTTTTTTTTTTTTTTTTTTTTTTTTTTT	GTTTTTTTTTTTTTTTTTTTTTTTTTTTTTTTTTTTTTTTTTTTTTTTTTTTTTTTTTTT	1	0	upstream_gene_variant	AHA1	YDR214W
IV			6 SNPs between 1525363 and 1525431	-	-	missense_variant	YDR544C	YDR544C
V	2084	T	C	1	0	missense_variant	YEL077C	YEL077C
V	2116	T	C	1	0	missense_variant	YEL077C	YEL077C
V	2146	T	C	1	0	missense_variant	YEL077C	YEL077C
V	20824	G	A	0	1	synonymous_variant	DSF1	YEL070W
V	205872	G	A	1	0	missense_variant	GCD11	YER025W
V	362749	A	T	0	1	upstream_gene_variant	PRS2	YER099C
VI	4716	CCCACACCCACACACCACACCCACACCACACCCACAC<br>ACCACACCCACACCACACC	CCCACACCCACACACCACACCCACACCACACC	0	1	upstream_gene_variant	YFL066C	YFL066C
VI	9922	T	C	1	0	synonymous_variant	DDI2	YFL061W
VI	11006	A	G	1	0	upstream_gene_variant	SNO3	YFL060C
VI	11211	G	T	1	0	upstream_gene_variant	SNO3	YFL060C
VI	11314	G	A	1	0	upstream_gene_variant	SNO3	YFL060C
VII	10981	T	A	1	0	upstream_gene_variant	PAU11	YGL261C
VII	364650	C	G	1	0	synonymous_variant	RPL7A	YGL076C
VII	364785	G	A	0	1	synonymous_variant	RPL7A	YGL076C
VII	535885	A	G	0	1	upstream_gene_variant	THG1	YGR024C
VIII			5 SNPs between 4844 and 6105	-	-	upstream_gene_variant	YHL050C	YHL050C
VIII	289318	A	G	0	1	synonymous_variant	AHT1	YHR093W
VIII	340058	TTATATATATATATATATATATATATATATATA	TTATATATATATATATATATATATATATATA	1	0	upstream_gene_variant	YHR112C	YHR112C
VIII	499958	C	T	0	1	missense_variant	PPX1	YHR201C
VIII	511255	C	T	0	1	synonymous_variant	SCH9	YHR205W
VIII	514829	CTATATATATATATATATATATATATATATA	CTATATATATATATATATATATATATATATATATA	1	0	upstream_gene_variant	BAT1	YHR208W
VIII			18 SNPs between 525628 and 529805	-	-	synonymous_variant	FLO5	YHR211W
VIII	549816	T	G	1	0	upstream_gene_variant	YHR214C-B	YHR214C-B
VIII	551421	G	A	1	0	missense_variant	YHR214C-E	YHR214C-E
VIII	551423	A	C	1	0	missense_variant	YHR214C-E	YHR214C-E
VIII	551439	A	T	1	0	missense_variant	YHR214C-E	YHR214C-E
VIII	551468	A	T	1	0	missense_variant	YHR214C-E	YHR214C-E
VIII	552371	G	A	0	1	synonymous_variant	PHO12	YHR215W
IX			6 SNPs between 206802 and 210095	-	-	missense_variant	YIL082W-A	YIL082W-A
IX	210176	C	T	1	0	upstream_gene_variant	tI(AAU)I2	tI(AAU)I2
IX	387143	C	A	0	1	upstream_gene_variant	MET28	YIR017C
IX			21 SNPs between 391339 and 392245	-	-	synonymous_variant	FLO11	YIR019C
IX			5 SNPs between 439814 and 439877	-	-	upstream_gene_variant	YIR042C	YIR042C
X	639694	C	A	1	0	upstream_gene_variant	YJR111C	YJR111C
X	639709	C	A	1	0	upstream_gene_variant	YJR111C	YJR111C
X	652909	G	T	0	1	missense_variant	YJR124C	YJR124C
X	652916	A	T	0	1	synonymous_variant	YJR124C	YJR124C
X			9 SNPs between 715095 and 715141	-	-	missense_variant	DAN4	YJR151C
X	730773	A	T	1	0	stop_gained	YJR157W	YJR157W
X	730835	C	A	1	0	stop_gained	YJR157W	YJR157W
X	731773	C	T	1	0	upstream_gene_variant	THI11	YJR156C
X	733265	G	A	1	0	missense_variant	HXT16	YJR158W
X	733998	C	T	1	0	missense_variant	HXT16	YJR158W
X	734783	G	A	1	0	upstream_gene_variant	SOR1	YJR159W
X			6 SNPs between 740157 and 742235	-	-	upstream_gene_variant	MPH3	YJR160C
X	743001	C	G	1	0	missense_variant	COS5	YJR161C
X	743217	T	G	1	0	synonymous_variant	COS5	YJR161C
X	745680	G	T	1	0	upstream_gene_variant	COS5	YJR161C
XI	28	A	C	1	0	upstream_gene_variant	YKL225W	YKL225W
XI	59567	T	G	0	1	missense_variant	TOR2	YKL203C
XI	164844	CTATATATATATATATATATATATATATATATATAT	CTATATATATATATATATATATATATATATATATATAT	0	1	upstream_gene_variant	RSM22	YKL155C
XI	541138	G	T	1	0	missense_variant	DYN1	YKR054C
XII	12277	C	T	1	0	upstream_gene_variant	YLL066C	YLL066C
XII	13020	C	T	1	0	upstream_gene_variant	YLL066C	YLL066C
XII			6 SNPs between 13122 and 13170	-	-	missense_variant	PAU18	YLL064C
XII	96700	A	G	.	1	synonymous_variant	SSA2	YLL024C
XII	220717	G	A	1	0	upstream_gene_variant	YLR035C-A	YLR035C-A
XII	460291	A	C	1	0	upstream_gene_variant	RDN37-1	RDN37-1
XII	474417	ACC	ACCC	1	0	upstream_gene_variant	ASP3-1	YLR155C
XII	734703	T	A	1	0	upstream_gene_variant	YLR302C	YLR302C
XII			9 SNPs between 1059473 and 1061528	-	-	upstream_gene_variant	NBP1	YLR457C
XIII	6154	A	T	0	1	upstream_gene_variant	YML133C	YML133C
XIII	6933	C	T	1	0	upstream_gene_variant	YML133C	YML133C
XIII	6941	G	A	1	0	upstream_gene_variant	YML133C	YML133C
XIII	812314	A	C	0	1	missense_variant	ZDS1	YMR273C
XIII	924337	T	G	1	0	upstream_gene_variant	YMR324C	YMR324C
XIII	924341	G	T	1	0	upstream_gene_variant	YMR324C	YMR324C
XIV	189585	C	T	1	0	missense_variant	SLA2	YNL243W
XIV	415047	CTTTTTT	CTTTTTTTTTTTTTTTTTTTTTTCGTTTTTTATTTTTTTTTTTTTTTTTTTTTTTATTTATTTTTTTTTTTTTTTTTTATTTTTTTTTTTTTTTTTTTTTTTTTTTTTTTTTTTT	0	1	upstream_gene_variant	YNL115C	YNL115C
XIV	415054	C	T	0	1	upstream_gene_variant	YNL115C	YNL115C
XIV	415088	C	T	1	0	upstream_gene_variant	YNL115C	YNL115C
XIV	415096	C	T	1	0	upstream_gene_variant	YNL115C	YNL115C
XIV	601677	T	C	1	0	missense_variant	YNL018C	YNL018C
XIV	601742	G	A	1	0	synonymous_variant	YNL018C	YNL018C
XV	78	CA	C	0	1	upstream_gene_variant	YOL166W-A	YOL166W-A
XV	97	A	C	1	0	upstream_gene_variant	YOL166W-A	YOL166W-A
XV	98	C	A	1	0	upstream_gene_variant	YOL166W-A	YOL166W-A
XV	108	A	C	1	0	upstream_gene_variant	YOL166W-A	YOL166W-A
XV	2294	G	C	0	1	upstream_gene_variant	YOL166C	YOL166C
XV	2345	G	A	0	1	upstream_gene_variant	YOL166C	YOL166C
XV	2398	T	C	0	1	upstream_gene_variant	YOL166C	YOL166C
XV			9 SNPsbetween 970965 and 971069	-	-	synonymous_variant	YOR343W-A	YOR343W-A
XVI	174252	T	C	1	0	synonymous_variant	RPL7B	YPL198W
XVI	174387	C	G	0	1	missense_variant	YPL197C	YPL197C
XVI	560663	T	A	1	0	missense_variant	YPR002C-A	YPR002C-A
XVI	667520	T	G	0	1	upstream_gene_variant	ATG11	YPR049C
XVI	769498	T	C	1	0	upstream_gene_variant	tK(UUU)P	tK(UUU)P
XVI	769532	G	A	1	0	upstream_gene_variant	tK(UUU)P	tK(UUU)P
XVI	769580	A	G	1	0	upstream_gene_variant	tK(UUU)P	tK(UUU)P
XVI	838970	T	A	1	0	upstream_gene_variant	TPO3	YPR156C
XVI			13 SNPs between 853008-853335	-	-	synonymous_variant	YPR158C-D	YPR158C-D

Supplemetary Table 10: Breakdancer CNV Analysis. The first six columns record the position(s) of the event and the open reading frame(s) detected. If the detected event is not a translocation, columns 1-3 are the same as coumns 4-6. Column 7 and 8 detail the type and size of the event: DEL (deletions), INS (insertion), INV (inversion), ITX (intra-chromosomal translocation), CTX (inter-chromosomal translocation). Columns 8-10 list the confidence score and the number of supporting read pairs for each strain. Events that are located in repetative regions such as telomeres, transposons, and ribosomal RNA genes are shaded light grey. Events which could be visually confirmed by alighning the reads to the S288C

Chr1	Pos1	ORF	Chr2	Pos2	ORF	Type	Size	Score	num_Reads_1691	num_Reads_1693
I	12474	intergenic (downstream of YAL064W-B)	I	12684	intergenic (downstream of YAL064W-B)	ITX	-287	99	23	18
I	26074	YAL063C (FLO9)	I	26510	YAL063C (FLO9)	ITX	-336	71	3	1
I	26891	YAL063C (FLO9)	I	27051	YAL063C (FLO9)	ITX	-304	99	3	7
II	469882	YBR115C (LYS2)	II	474220	YBR115C (LYS2)	DEL	4506	99	25	33
II	469882	YBR115C (LYS2)	II	474469	YBR115C (LYS2)	DEL	4740	70	2	1
II	469882	YBR115C (LYS2)	II	474602	YBR115C (LYS2)	DEL	4862	71	1	2
III	17414	YCL063W	III	17458	YCL063W	INS	-471	99	1	2
III	84509	YCL019W (LEU2)	III	92480	YCL019W (LEU2)	DEL	7917	99	1	4
III	84939	YCL019W (LEU2)	III	92480	YCL019W (LEU2)	DEL	7692	99	24	51
III	268980	YCR089W (known duplication)	III	269334	YCR089W (known duplication)	ITX	-338	94	2	2
III	200940	YCR039C/YCR040W (ALPHA2/ALPHA1	III	294607	YCR098C (GIT1)	ITX	92452	99	23	26
IV	758478	YDR150W	IV	758586	YDR150W	ITX	-334	73	3	1
IV	1525372	telomere	IV	1525452	YDR544C	ITX	-315	99	4	6
IX	25362	telomere	IX	25535	YIL169C	ITX	-293	99	3	4
Mito	6913		Mito	47815		INV	23166	99	13	23
Mito	11215		Mito	47815		INV	1890	99	7	16
Mito	13618		Mito	47815		INV	23840	93	3	9
Mito	47815		Mito	73182		INV	6251	99	17	23
Mito	47815		Mito	80215		INV	6079	99	7	10
Mito	47815		Mito	85980		INV	153	99	5	9
Mito	1201		Mito	83430		INV	81133	93	4	4
Mito	1		Mito	85980		ITX	84733	99	180	350
Mito	6913		Mito	16970		INV	1105	69	2	6
Mito	6913		Mito	31030		INV	25843	99	8	9
Mito	6913		Mito	36806		INV	28862	71	4	6
Mito	6913		Mito	52951		DEL	51683	73	4	9
Mito	6913		Mito	64262		DEL	65288	99	12	11
Mito	6913		Mito	83430		INV	77862	99	21	17
Mito	6913		Mito	85980		INV	80059	99	8	5
Mito	11215		Mito	80027		DEL	71235	99	7	6
Mito	11990		Mito	80027		DEL	70487	60	2	4
Mito	13618		Mito	31030		INV	17056	99	2	7
Mito	13618		Mito	83430		INV	69308	80	3	4
Mito	31030		Mito	60441		INV	24625	99	9	9
Mito	29316		Mito	83430		ITX	51070	58	13	12
Mito	36806		Mito	53145		INV	15660	99	8	7
Mito	36806		Mito	60441		INV	22111	99	14	18
Mito	53145		Mito	73182		INV	19234	53	4	5
Mito	53145		Mito	83430		INV	29092	74	4	5
Mito	60441		Mito	73182		INV	11493	97	6	9
Mito	60441		Mito	83430		INV	26832	99	16	14
Mito	60441		Mito	85980		INV	27581	68	4	5
V	116108	YEL021W (URA3)	V	117045	YEL021W (URA3)	DEL	1135	99	30	33
VII	356826	YGL082W (known duplication)	VII	356858	YGL082W (known duplication)	INS	-490	99	54	111
I	1	telomere	VIII	562821	intergenic (downsteram of YHR219W)	ITX	-427	51	4	4
X	508773	YJR040W	X	508807	YJR040W	INS	-466	99	1	1
X	713026	YJR151C (DAN4)	X	713371	YJR151C (DAN4)	ITX	-305	99	3	2
VIII	556799	telomere	XII	363	telomere	ITX	-355	99	7	4
II	165870	intergenic (upstream of YBL029W)	XII	460787	intergenic (upstream of RDN37-2)	ITX	-346	99	19	23
XII	732412	YLR303W (MET15)	XII	734686	YLR303W (MET15)	DEL	2456	99	31	0
XIII	908139	telomere	XIII	908743	telomere	ITX	-378	99	17	12
XIV	704446	YNR044W (known duplication)	XIV	704473	YNR044W (known duplication)	ITX	-282	67	1	1
IX	23044	telomere	XV	28722	YOL019W (known duplication)	CTX	-510	99	6	15
IX	23880	telomere	XV	28722	YOL019W (known duplication)	CTX	-502	99	17	16

Supplemetary Table 11: CNV.kit Analysis. Only those genome regions with a copy number different than 1 are shown. Events that are located in repetative regions such as telomeres, transposons, and ribosomal RNA genes are shaded light grey. Events which could be visually confirmed by alighning the reads to the S288C reference genome in IGV are shaded dark grey.

1691				
chromosome	start	end	ORF	copy numer
X	472681	483859	YJR026W, YJR027W, YJR029W (transposons)	2
Mito	0	85779	mitochondria	22
VII	535087	543074	YGR027W-A (transposon), YGR027W-B (transposon), tE(UUC)G3, YGR028W	2
XII	450438	469605	RDN25-1, RDN37-1, RDN18-1, RDN5-1, RDN37-2, RDN18-2, RDN5-2	94
XV	116638	123029	YOL104C, YOL103W-B (transposon)	2
XVI	844146	856936	YPR158W-A, YPR158W-B, YPR158C-D, YPR158C-C (transposons)	2

1693				
chromosome	start	end	ORF	copy number
II	469902	474122	YBR115C (lys2)	0
IV	528009	537866	YDR38C (ENA5), YDR39C (ENA2), YDR40C (ENA1)	2
IV	987026	992658	YDR261C-D, YDR261C-C (transposons)	2
Mito	0	85779	mitochondria	29
VII	534912	541950	YGR027W-A (transposon), YGR027W-B (transposon), tE(UUC)G3, YGR028W	2
VIII	212397	216617	RUF5-1, YHR054C (known partial duplication), RUF5-2, YHR056C (known duplicat	15
XII	451820	468711	RDN25-1, RDN37-1, RDN18-1, RDN5-1, RDN37-2, RDN18-2, RDN5-2	94
XII	1068324	1078177	telomere	2

Supplemetary Table 12: CNVnator Analysis, bin size = 20. Events that are located in repetative regions such as telomeres, transposons, and ribosomal RNA genes are shaded light grey. Events which could be visually confirmed by alighning the reads to the S288C reference genome in IGV are shaded dark grey.

1691			
CNV type	coordinates	context/ORF	CNV_size
duplication	I:1-23720	telomere	23720
duplication	I:23761-30780	YAL063C (flo9)	7020
duplication	I:48301-50480	YAL051W (known duplicate)	2180
duplication	I:63561-65760	YAL04W1	2200
deletion	I:65761-67520	YAL040C (cln3)	1760
duplication	I:159741-189920	YAR009C (transposon)	30180
deletion	I:189921-192100	YAR035W	2180
duplication	I:196221-203860	telomere	7640
deletion	I:204601-205760	telomere	1160
duplication	I:206261-210840	telomere	4580
duplication	I:214221-230220	telomere	16000
duplication	II:1-30640	telomere	30640
deletion	II:30641-31860	YBL100W-B (transposon)	1220
duplication	II:31861-54220	YBL100W-B (transposon)	22360
duplication	II:108181-110580	YBL060W, YBL059C-A	2400
duplication	II:222841-226960	YBL005W-B (transposon)	4120
deletion	II:261281-261520	YBR012W-B (transposon)	240
duplication	II:261541-263020	YBR012W-B (transposon)	1480
deletion	II:264141-264400	YBR012W-B (transposon)	260
duplication	II:295201-296940	YBR028C, YBR029C	1740
deletion	II:455401-457500	YBR108W	2100
deletion	II:469701-474240	YBR115C (lys2)	4540
duplication	II:642341-647840	YBR209W, tE(UUC)B, YBR210W (known duplicate) , YBR211C	5500
duplication	II:653161-657600	YBR215W, YBR216C (known duplicate)	4440
duplication	II:751621-813200	telomere	61580
duplication	III:1-12140	telomere	12140
duplication	III:13921-47320	YCL067C (HMLALPHA2), YCL066W (HLMAPHA1)	33400
duplication	III:80141-84780	YCL024W (known duplicate), YCL022C, tE(UUC)C, YCL021W-A	4640
deletion	III:84781-88320	YCL019W (leu2)	3540
deletion	III:90661-92480	YCL019W (leu2)	1820
deletion	III:148601-151720	telomere	3120
duplication	III:168381-170600	telomere	2220
deletion	III:250941-252440	YCR077C	1500
duplication	III:255001-316580	telomere	61580
duplication	IV:1-31360	telomere	31360
duplication	IV:46521-48460	YDL227C	1940
deletion	IV:352881-355380	YDL056W (mbp1)	2500
duplication	IV:514301-515700	YDR034C-D (transposon)	1400
duplication	IV:528741-531500	YDR038C (ENA5)	2760
duplication	IV:531881-537560	YDR039C (ENA2)	5680
duplication	IV:645761-651740	YDR098C-B (transposon)	5980
duplication	IV:875561-883880	YDR210C-D (transposon)	8320
duplication	IV:985221-992800	YDR261C-D (transposon)	7580
duplication	IV:1095961-1102200	YDR316W-B (transposon)	6240
duplication	IV:1206821-1212420	YDR36W-B (transposon)	5600
duplication	IV:1480681-1531940	telomere	51260
duplication	IX:1-2220	telomere	2220
duplication	IX:3861-18480	telomere	14620
deletion	IX:18741-18980	telomere	240
duplication	IX:18981-22000	telomere	3020
duplication	IX:23461-36820	YIL169C, YIL168W, YIL166C, YIL165C	13360
duplication	IX:42761-46520	YIL159W, YIL158W (known duplicate)	3760
duplication	IX:270881-274140	YIL045W (known duplicate), YIL044C	3260
duplication	IX:391661-439800	telomere	48140
duplication	Mito:1-8980	mitochondrial	8980
duplication	Mito:9021-47660	mitochondrial	38640
duplication	Mito:47881-56900	mitochondrial	9020
duplication	Mito:57021-66700	mitochondrial	9680
duplication	Mito:66741-70560	mitochondrial	3820
duplication	Mito:70661-72860	mitochondrial	2200
duplication	Mito:72961-85780	mitochondrial	12820
duplication	V:1-40040	telomere	40040
duplication	V:45001-65300	YEL056W, YEL055C, snR80, YEL053C, YEL052W, YEL050C, snR67, YEL049W	20300
deletion	V:115921-117060	YEL021W (URA3)	1140
duplication	V:443761-449040	YER138C (transposon)	5280
deletion	V:473021-476100	YER152C, YER152W-A, YER153C, YER154W	3080
duplication	V:492601-498740	YER160C (transposon)	6140
duplication	V:523081-544100	YER169W (known duplication), YER170W, YER171W, YER172C, YER172C-A, YER174C (known duplication), YER175W-A, YER176W	21020
duplication	V:545621-576880	telomere	31260
duplication	VI:1-48120	telomere	48120
duplication	VI:84961-88940	YFL025C, YFL024C	3980
duplication	VI:109881-112600	YFL012W-A, YFL012W, YFL011W	2720
duplication	VI:137041-138260	YFL003C, tN(GUU)F	1220
duplication	VI:140721-145400	YFL002W-A (transposon)	4680
duplication	VI:188481-197060	YFR019W, tS(GCU)F, YFR020W, YFR012W (known duplicate)	8580
duplication	VI:199821-201580	YFR023W (known duplicate)	1760
duplication	VI:232061-240040	YFR039C (known duplicate), YFR040W (known duplicate), YFR041C, YFR042W, YFR043C	7980
duplication	VI:242341-270140	telomere	27800
duplication	VII:1-65380	telomere	65380
duplication	VII:69501-71000	YGL227W	1500
duplication	VII:110381-113880	tH(GUG)G1, YGL204C, YGL203C	3500
duplication	VII:147381-148880	YGL189C (known duplication)	1500
deletion	VII:383781-385360	YGL063W (known duplication), YGL063C-A, YGL062W (known duplication)	1580
duplication	VII:496241-498020	YGL001C, YGR001C	1780
duplication	VII:534901-541700	YGR027W-A (transposon)	6800
duplication	VII:562341-569040	YGR038C-B (transposon)	6700
duplication	VII:618041-623060	YGR065C, YGR066C, YGR067C	5020
duplication	VII:711601-713960	YGR109W-B (transposon), YGR110W	2360
duplication	VII:818021-823500	YGR161C-D (transposon)	5480
duplication	VII:909761-912260	YGR205W, YGR206W, YGR207C, YGR208W	2500
duplication	VII:922541-923860	YGR216C	1320
duplication	VII:930881-932000	tG(GCC)G2	1120
duplication	VII:956381-958260	YGR233C	1880
duplication	VII:1007441-1009500	YGR258C	2060
duplication	VII:1037641-1090960	telomere	53320
duplication	VIII:1-48700	telomere	48700
duplication	VIII:71021-72800	YHL017W (known duplication), YHL016C	1780
duplication	VIII:74481-92460	YHL009W-B (transposon)	17980

1693			
CNV type	coordinates	context/ORF	CNV_size
duplication	I:1-25540	telomere	25540
duplication	I:26901-31460	YAL063C (FLO9)	4560
duplication	I:38541-40700	YAL058W, YAL056W (known duplication)	2160
deletion	I:65781-69840	YAL040C (CLN3)	4060
duplication	I:159981-162760	YAR009C (transposon)	2780
duplication	I:165241-166480	YAR010C (transposon), tA(UGC)A	1240
duplication	I:175041-189220	YAR020C, YAR023C, tL(CAA)A, tS(AGA)A, YAR027W, YAR028W,	14180
duplication	I:194561-203780	telomere	9220
deletion	I:204581-205340	telomere	760
duplication	I:205781-211960	telomere	6180
deletion	I:211961-212200	telomere	240
deletion	I:213941-214180	telomere	240
duplication	I:214181-220000	telomere	5820
duplication	I:223341-230220	telomere	6880
duplication	II:1-30200	telomere	30200
deletion	II:30201-31780	YBL100W-B (transposon)	1580
duplication	II:31781-36300	YBL100W-B (transposon)	4520
deletion	II:469701-474240	YBR115C (LYS2)	4540
duplication	III:1-11880	telomere	11880
duplication	III:14041-46920	YCL064C, YCL061C, YCL059C, YCL057W, YCL055W,	32880
deletion	III:84781-88340	YCL019W (LEU2)	3560
deletion	III:90521-92480	YCL019W (LEU2)	1960
deletion	III:148601-151720	YCR018C, tM(CAU)C, tK(CUU)C	3120
duplication	III:293421-314100	telomere	20680
duplication	IV:1-15560	telomere	15560
deletion	IV:302941-304400	YDL085W (known duplication)	1460
deletion	IV:352881-355380	YDL056W (MBP1)	2500
duplication	IV:528561-531500	YDR038C (ENA5)	2940
deletion	IV:531501-531760	YDR039C (ENA2)	260
duplication	IV:532581-537120	YDR40C (ENA1)	4540
duplication	IV:645381-652160	YDR098C-B,A (transposn)	6780
duplication	IV:987561-992660	YDR261C-D,C (transposon)	5100
duplication	IV:1096641-1101680	YDR261C-D,C (transposon)	5040
duplication	IV:1207321-1212260	YDR365W-B (transposon)	4940
deletion	IV:1333441-1334700	YDR435C	1260
duplication	IV:1525881-1531940	telomere	6060
duplication	IX:1-2280	telomere	2280
duplication	IX:7261-18440	telomere	11180
duplication	IX:196041-197840	telomere	1800
duplication	IX:383481-439900	telomere	56420
duplication	Mito:1-2960	mitochondrial	2960
duplication	Mito:3021-34060	mitochondrial	31040
duplication	Mito:34161-52380	mitochondrial	18220
duplication	Mito:52501-70560	mitochondrial	18060
duplication	Mito:70661-72900	mitochondrial	2240
duplication	Mito:73201-77100	mitochondrial	3900
duplication	Mito:77221-81480	mitochondrial	4260
duplication	Mito:81521-85780	mitochondrial	4260
duplication	V:1-39080	telomere	39080
deletion	V:115921-117060	YEL021W (URA3)	1140
duplication	V:443621-448260	YER138C (transposon)	4640
deletion	V:472361-475940	YER152C, YER152W-A, YER153C, YER154W	3580
duplication	V:492601-497520	YER160C (transposon)	4920
duplication	V:561701-576880	telomere	15180
duplication	VI:4981-12860	YFL061W (DDI2), YFL060C (SNO3)	7880
duplication	VI:143381-146340	YFL002W-A (transposon)	2960
duplication	VII:1-52960		52960
deletion	VII:270801-272640	YGL126W, YGL125W	1840
duplication	VII:535521-538220	YGR027W-A (transposon)	2700
duplication	VII:539621-541540	YGR027W-A (transposon)	1920
duplication	VII:562101-568880	YGR038C-B (trasnposon)	6780
duplication	VII:817521-823940	YGR161C-D (transposon)	6420
duplication	VII:1046041-1047900	YGR277C, YGR278W	1860
duplication	VII:1080841-1090960	telomere	10120
duplication	VIII:1-23520	telomere	23520
duplication	VIII:212381-216140	RUF5-1, YHR054C (known partial duplication), RUF5-2, YHR056C (known duplication)	3760
deletion	VIII:390601-391600	YHR146W (known duplication)	1000
duplication	VIII:526301-537700	telomere	11400
duplication	VIII:543621-545680	telomere	2060
duplication	VIII:547241-557160	telomere	9920
deletion	VIII:557721-557960	telomere	240
duplication	VIII:557961-562620	telomere	4660
duplication	X:1-19220	telomere	19220
duplication	X:72701-74820	YJL192C, YJL191W (ribosomal protein)	2120
deletion	X:360221-362040	YJL043W, YJL042W	1820
deletion	X:362081-363520	YJL042W	1440
duplication	X:471541-483840	YJR026W, YJR027W, YJR029W (transposons)	12300
deletion	X:637941-640340	upstream of YJR115W (known duplication)	2400
duplication	X:715481-731580	telomere	16100
deletion	X:731621-731900	telomere	280
deletion	X:732801-734900	telomere	2100
duplication	X:735141-737200	telomere	2060
duplication	X:737241-745760	telomere	8520
duplication	XI:1-12840	telomere	12840
deletion	XI:66581-69500	YKL201C (known duplication), YKL198C (known duplication)	2920
deletion	XI:170201-173780	YKL148C, YKL147C, YKL146W	3580
deletion	XI:525481-527260	YKR047W, YKR048C, YKR049C	1780
duplication	XI:635721-666820	telomere	31100
duplication	XII:5541-20060	telomere	14520
deletion	XII:216321-217240	YLR035C-A (transposon)	920
deletion	XII:329801-330960	YLR095C	1160
duplication	XII:451381-468840	RDN25-1, RDN37-1, YLR154W-F, RND5-1 RDN37-2, RDN18-2	17460
duplication	XII:476221-478220	YLR157C-B (transposon)	2000
deletion	XII:479341-479900	YLR157C-B (transposon)	560
deletion	XII:481721-482140	YLR157C-A (transposon)	420
duplication	XII:490041-490320	upstream of YLR162W-A	280
duplication	XII:593781-598680	YLR227W-B (transposon)	4900
duplication	XII:651481-652880	YLR256W-A (transposon)	1400

duplication	VIII:112881-120180	YHR005C, YHR005C-A, YHR006W (known duplication)	7300
duplication	VIII:212381-216140	RUF5, YHR054C	3760
duplication	VIII:472581-475180	YHR184W, YHR185C	2600
duplication	VIII:525161-548600	telomere	23440
deletion	VIII:548601-548840	telomere	240
duplication	VIII:548841-557720	telomere	8880
deletion	VIII:557721-557960	telomere	240
duplication	VIII:557961-562660	telomere	4700
duplication	X:1-20100	telomere	20100
duplication	X:28221-35320	YJL214W, YJL213W, YJL212C	7100
duplication	X:38601-48180	YJL209W, YJL208C, YJL207C, YJL206C	9580
deletion	X:120421-121800	YJL159W (known duplication)	1380
duplication	X:472521-483960	YJR026W, YJR027W, YJR029W (transposons)	11440
deletion	X:638901-640260	YJR113C (ribosomal protein), YJR115W (known duplication)	1360
duplication	X:721361-731460	telomere	10100
deletion	X:731461-734220	telomere	2760
deletion	X:734641-734900	telomere	260
duplication	X:734901-745760	telomere	10860
duplication	XI:1-51140	telomere	51140
duplication	XI:361501-363400	YKL040C, YKL039W (known duplication)	1900
duplication	XI:373521-378920	YKL034W, YKL033W-A, YKL033W	5400
duplication	XI:430241-431780	YKL006C, snR87	1540
deletion	XI:527861-529340	YKR050W (known duplication)	1480
duplication	XI:602601-606000	YKR086W, YKR087C, YKR088C, YKR089C (known duplication)	3400
duplication	XI:619541-666820	telomere	47280
deletion	XII:441-700	telomere	260
duplication	XII:701-20140	telomere	19440
duplication	XII:20181-59380	telomere	39200
duplication	XII:208741-216400	YLR035C-A (transposon)	7660
deletion	XII:216401-217720	YLR035C-A (transposon)	1320
duplication	XII:217721-244020	YLR035C-A (transposon), YLR036C, YLR038C, YLR039C, YLR040C, YLR043C (known duplication), YLR045C, YLR046C (known duplication), YLR047C, YR048W (known duplication)	26300
duplication	XII:412301-413760	YLR134W, YLR135W	1460
duplication	XII:436201-437460	YLR148W	1260
duplication	XII:451461-469960	RDN25-1, RDN37-1, YLR154W-F, RDN5-1, RDN37-2, RDN18-2, RDN5-2	18500
deletion	XII:474301-474580	intergenic, adjacent to transposon	280
deletion	XII:475841-476060	YLR157W-D (known duplication, adjacent to transposon)	220
duplication	XII:476081-478180	YLR157C-B (transposon)	2100
duplication	XII:480081-481620	YLR157C-A (transposon)	1540
duplication	XII:483721-485860	YLR159W (known duplication), RDN5-5	2140
duplication	XII:487141-489140	YLR160C (known duplication, near rNDA), YLR161W (known duplication, near rDNA)	2000
duplication	XII:490041-490320	intergenic near unknown proteins	280
duplication	XII:593861-599740	YLR227W-B (transposon)	5880
duplication	XII:650901-657860	YLR256W-A (transposon)	6960
deletion	XII:732281-734700	YLR303W (MET15)	2420
duplication	XII:748701-751900	YLR309C	3200
duplication	XII:792421-794180	tD(GUC)L2	1760
duplication	XII:794221-795640	snR61, snR55, snR57, YLR333C (ribosomal)	1420
duplication	XII:944341-947220	YLR410W-B (transposon)	2880
duplication	XII:976761-981600		4840
duplication	XII:1028761-1034320	YLR448W (ribosomal), YLR449W, YLR450W	5560
duplication	XII:1036941-1078180	telomere	41240
duplication	XIII:1-32760	telomere	32760
duplication	XIII:184261-190060	YML045W (transposon)	5800
duplication	XIII:197501-198380	YML039W (transposon)	880
duplication	XIII:198621-202340	YML039W (transposon)	3720
duplication	XIII:356301-363100	YMR045C (transposon)	6800
duplication	XIII:372681-378700	YMR050C (transposon)	6020
deletion	XIII:388021-389960	YMR056C, YMR057C, YMR058W	1940
duplication	XIII:502741-505060	tL(CAA)M	2320
duplication	XIII:648641-650060	YMR129W	1420
duplication	XIII:841081-857240	YMR287C, YMR288W (snRNP), YMR289W, YMR291W, YMR292W, YMR293C	16160
duplication	XIII:863401-924440	telomere	61040
duplication	XIV:6621-43160	telomere	36540
duplication	XIV:96621-100700	YNL284C-B	4080
deletion	XIV:196481-200380	YNL241C, YNL240C	3900
duplication	XIV:520941-525120	YNL054W-B (transposon)	4180
duplication	XIV:561181-563820		2640
duplication	XIV:672441-674320	YNR024W, YNR025C, YNR026C (known duplication)	1880
duplication	XIV:715921-754020	YNR052C, snR191, tL(UAA)N, YNR055C, YNR056C, YNR058W, YNR059W, YNR060W, YNR062C, YNR063W, YNR065C	38100
duplication	XIV:754061-784340	telomere	30280
duplication	XV:1-53600	telomere	53600
duplication	XV:97381-99040	YOL117W	1660
duplication	XV:110821-113620	tG(UCC)O, YOL108C, YOL107W, YOL106W	2800
duplication	XV:117841-123720	YOL103W-B (transposon)	5880
duplication	XV:179641-181920	YOL081W (known duplication), YOL080C, YOL079W, YOL078W	2280
duplication	XV:226641-228760	tG(GCC)O1, tN(GUU)O1, YOL054W	2120
duplication	XV:299281-300620	YOL014W	1340
duplication	XV:436221-439080	YOR058C, tK(UUU)O, YOR059C	2860
deletion	XV:479621-481280	YOR083W (WHI5)	1660
duplication	XV:594301-600620	YDR142W-B (transposon)	6320
deletion	XV:637081-639680	YOR161C, YOR161W-B, YOR161C-C, YOR162C (known duplication)	2600
deletion	XV:676061-677940	YOR181W	1880
duplication	XV:703441-709040	YOR192C-B (transposon)	5600
deletion	XV:722241-722460	YOR202W (HIS3)	220
duplication	XV:972901-976300	YOR343W-B (transposon)	3400
duplication	XV:1065781-1091280	telomere	25500
duplication	XVI:1-62880	telomere	62880
duplication	XVI:435841-440720	YPL060C-A (transposon)	4880
duplication	XVI:804641-810700	YPR137C-B (transposon)	6060
duplication	XVI:844301-855920	YPR158W-A,B, YPR158C-C,D (transposons)	11620
duplication	XVI:881041-883680	YPR170C, YPR169W-A	2640
duplication	XVI:923401-947800	telomere	24400

duplication	XII:654281-656760	YLR256W-A (transposon)	2480
duplication	XII:945781-946980	YLR410W-B (transposon)	1200
duplication	XII:1069281-1078180	telomere	8900
duplication	XIII:1-14980	telomere	14980
deletion	XIII:68021-73040	YML102W, YML101C-A, YML101C (known duplication), YML100W (known dupliction)	5020
duplication	XIII:184041-190120	YML045W (transposon)	6080
duplication	XIII:199181-202340	YML039W (transposon)	3160
duplication	XIII:372781-377660	YMR050C (transposon)	4880
deletion	XIII:489161-490320	YMR109W (known duplication)	1160
deletion	XIII:817581-820100	YMR275C (known duplication), YMR276W	2520
duplication	XIII:908121-924400	telomere	16280
duplication	XIV:1-11520	telomere	11520
duplication	XIV:14741-18160	YNL332W, YNL331C, YNL330C	3420
duplication	XIV:96781-100660	YNL284C-B (transposon)	3880
duplication	XIV:561361-563400	between tT(AGU)N2 and tP(UGG)N2	2040
duplication	XIV:725481-784340	telomere	58860
duplication	XV:1-29400	telomere	29400
duplication	XV:119001-123400	YOL103W-B (transposon)	4400
duplication	XV:226141-228920	tG(GCC)O1, tN(GUU)O1, YOL054W	2780
deletion	XV:480041-482540	YOR083W, YOR084W, YOR085W	2500
deletion	XV:587881-589800	YOR140W	1920
duplication	XV:598561-600640	YDR142W-B (transposon)	2080
deletion	XV:676221-677980	YOR181W (LAS17)	1760
duplication	XV:708001-709100	YOR181W (LAS17)	1100
deletion	XV:921801-922620	YOR323C	820
duplication	XV:974801-978240	YOR343W-B (transposon)	3440
duplication	XV:1084821-1091300	telomere	6480
duplication	XVI:1-12480	telomere	12480
duplication	XVI:59921-63140	YPL257W-B (transposon)	3220
duplication	XVI:805661-808860	YPR137C-B (transposon)	3200
duplication	XVI:847841-855960	YPR158W-A,B, YPR158C-C,D (transposons)	8120

Supplemetary Table 13: CNVnator Analysis, bin size = 100. Events that are located in repetative regions such as telomeres, transposons, and ribosomal RNA genes are shaded light grey. Events which could be visually confirmed by alighning the reads t

1691			
CNV type	coordinates	ORF(s) impacted	CNV_size
duplication	I:1-27500	telomere	27500
duplication	I:192001-230500	telomere	38500
duplication	II:1-30500	telomere	30500
deletion	II:470001-474000	YBR115C (LYS2)	4000
duplication	II:760001-813500	telomere	53500
duplication	III:1-32500	telomere	32500
deletion	III:85001-89000	YCL018W (LEU2)	4000
deletion	III:91001-92500	YCL018W (LEU2)	1500
deletion	III:148501-151500	YCR018C, tM(CAU)C, tK(CUU)C	3000
duplication	III:293501-317000	telomere	23500
duplication	IV:1-14500	telomere	14500
deletion	IV:353001-355500	YDL056W (MBP1)	2500
duplication	IV:874001-885000	YDR210W-B, YDR210C-D, YDR210C-C (transposons)	11000
duplication	IV:1524001-1531500	telomere	7500
duplication	IX:405501-440000	telomere	34500
duplication	VI:1-13000	telomere	13000
duplication	VII:1-28000	telomere	28000
duplication	VIII:1-24000	telomere	24000
duplication	VIII:529001-563000	telomere	34000
duplication	X:1-20000	telomere	20000
duplication	X:472001-484000	YJR026W,YJR027W, YJR029W (transposons)	12000
duplication	X:704501-746000	telomere	41500
duplication	XII:1-54500	telomere	54500
duplication	XII:451501-471500	RDN25-1, RDN37-1, RDN18-1, RDN5-1, RDN37-2, RDN18-2, RDN5-2	20000
deletion	XII:732501-734500	YLR303W (MET17)	2000
duplication	XII:1056501-1078000	telomere	21500
duplication	XIV:754001-784500	telomere	30500
duplication	XV:1-29500	telomere	29500
duplication	XVI:6001-63000	telomere	57000
duplication	XVI:846001-857000	YPR158W-B, YPR158C-D, YPR158C-C (transposons)	11000

1693			
CNV type	coordinates	ORF(s) impacted	CNV_size
duplication	I:1-66000	telomere	66000
deletion	I:66001-67500	YAL040C(CLN3)	1500
duplication	I:194501-230500	telomere	36000
duplication	II:1-30000	telomere	30000
deletion	II:470001-474000	YBR115C (LYS2)	4000
duplication	III:1-29000	telomere	29000
deletion	III:85001-88500	YCL018W (LEU2)	3500
deletion	III:90501-92500	YCL018W (LEU2)	2000
deletion	III:148501-151500	YCR018C, tM(CAU)C, tK(CUU)C	3000
duplication	III:293001-317000	telomere	24000
duplication	IV:1-14500	telomere	14500
deletion	IV:353001-355500	YDL056W (MBP1)	2500
deletion	V:116001-117000	YEL021W (URA3)	1000
duplication	VI:1-13000	telomere	13000
duplication	VII:1-32500	telomere	32500
duplication	VIII:516001-563000	telomere	47000
duplication	X:471501-484000	YJR026W,YJR027W, YJR029W (transposons)	12500
duplication	X:735001-746000	telomere	11000
duplication	XII:1-50500	telomere	50500
duplication	XII:451501-473500	RDN25-1, RDN37-1, RDN18-1, RDN5-1, RDN37-2, RDN18-2, RDN5-2, YLR156W	22000
duplication	XIV:746001-784500	telomere	38500
duplication	XV:1-29500	telomere	29500
duplication	XVI:1-65500	telomere	65500
